# Titanium Dioxide Induced Immobilization of Small Platinum Nanoparticles on Carbon Spherogels: A Strategy Towards Stable and Efficient Electrocatalysts for the Hydrogen Evolution Reaction

**DOI:** 10.1002/smll.202512049

**Published:** 2026-03-26

**Authors:** Philip S. Pein, Ala Alsuhile, Simon Penner, Asghar Mohammadi, Gregor A. Zickler, Aseel Almohammad, Finn Baron, Mathis Kirstein, Can Erkey, Irina Smirnova, Michael S. Elsaesser, Baldur Schroeter

**Affiliations:** ^1^ Institute of Thermal Separation Processes Hamburg University of Technology Hamburg Germany; ^2^ Chemical and Biological Engineering Department Koç University Istanbul Turkey; ^3^ Koç University Hydrogen Technologies Center (KUHyTech) Istanbul Turkey; ^4^ Institute of Physical Chemistry University of Innsbruck Innsbruck Austria; ^5^ Department Chemistry and Physics of Materials University of Salzburg Salzburg Austria; ^6^ United Nations University Hub on Engineering to Face Climate Change at the Hamburg University of Technology United Nations University Institute for Water, Environment and Health (UNU‐INWEH) Hamburg Germany

**Keywords:** carbon spherogels, electrocatalysts, hollow carbon nanospheres, hydrogen evolution reaction, Pt‐nanoparticle immobilization, supercritical deposition

## Abstract

This study introduces a new strategy to electrocatalyst synthesis by immobilizing platinum nanoparticles (Pt‐NPs) on carbon spherogels—nanoporous, monodisperse carbon hollow spheres with diameters of 170–240 nm and surface areas of up to 800 m^2^ g^−1^ with or without incorporated titanium‐dioxide (TiO_2_) sublayers, using supercritical deposition. The resulting materials feature precisely tunable Pt‐loadings (2–11 wt%), narrow Pt‐NP size distributions, low interparticle distances (4.4–8.2 nm), and high Pt‐NP dispersion (Pt‐NP mean diameter 2.2–3.5 nm). The presence of TiO_2_ sublayers enhances both catalytic activity and durability in the hydrogen evolution reaction compared to a commercial Pt/C benchmark with similar Pt content. TiO_2_ containing electrocatalysts exhibit Pt‐NPs in the inner part of spheres and outstanding stability, demonstrated by (a) minimal potential shifts (1–4 mV) after accelerated stability tests, (b) suppression of Pt‐NP growth and detachment, and (c) structural integrity retention after 70 h under harsh conditions. These findings highlight the potential of spherogels as advanced catalyst supports and offer a scalable synthesis route without requirement for hazardous templating agents. Thanks to the tunable support morphology, precise Pt‐NP deposition, and remarkable long‐term performance, this approach emerges as a strong candidate for designing next generation electrocatalysts.

## Graphical abstract

Platinum nanoparticles are immobilized on hollow carbon spherogels using supercritical deposition, enabling precise control over nanoparticle size, loading, and spatial distribution. Incorporation of an internal TiO_2_ sublayer in the hollow sphere support induces enhanced metal–support interactions, markedly improving hydrogen evolution activity and long‐term stability. This scalable, HF‐free strategy establishes spherogels as advanced supports for durable low‐platinum electrocatalysts.

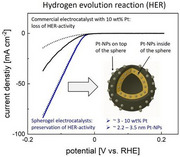

## 1 Introduction

Carbon‐supported platinum (Pt) is among the most widely used electrocatalysts for the hydrogen evolution reaction (HER) in acidic media [1]. As global efforts intensify to decarbonize the energy sector, water electrolysis, particularly when powered by renewable electricity, has become a central technology for producing sustainable “green hydrogen” [2]. Proton exchange membrane (PEM) electrolyzers are expected to expand from today's demonstration‐scale systems to several hundred gigawatts of capacity by 2030, which places increasingly stringent demands on the performance, stability, and material efficiency of HER electrocatalysts [3]. Commercial Pt/C electrocatalysts typically rely on relatively high noble metal loadings to ensure the required catalytic activity [4]. Reducing Pt usage while maintaining or even improving electrochemical performance has therefore become a key challenge, not only scientifically but also economically [5]. In this context, the International Renewable Energy Agency (IRENA) has identified the optimization of electrocatalysts as one of the most effective levers for lowering the overall cost of green hydrogen production [6].

A well‐established route toward lowering Pt loading is to enhance metal dispersion by employing small Pt nanoparticles (Pt‐NPs) [7]. While this improves the accessible Pt‐surface area, such nanoscale particles are thermodynamically unstable owing to their high surface energies and hence tend to agglomerate or sinter under operating conditions [8, 9]. These processes are particularly pronounced when Pt‐NPs are both small and closely spaced, or when surface mobility is high [10]. Maintaining stability therefore depends critically on the metal–support interaction (MSI) [11]. Carbon black, which still dominates commercial catalyst architectures, offers high electrical conductivity and chemical inertness but provides only weak interactions with Pt. Consequently, Pt‐NPs on carbon are susceptible to particle migration, coalescence, and detachment during electrochemical operation [12, 13, 14]. Introducing meso‐ and microporosity can physically confine Pt‐NPs and mitigate their agglomeration [14, 15, 16]. However, non‐activated, conventional carbon blacks exhibit only low microporosity and limited specific surface areas, which restrict their ability to effectively confine Pt NPs [17].

In contrast, metal‐oxide containing supports such as SnO_2_, WO_3_, and CeO_2_ exhibit stronger MSI and higher chemical stability: among these, TiO_2_ has received particular interest due to its broad applicability across electrocatalytic reactions [18]. Strong metal–support interactions (SMSI) at Pt/TiO_2_ interfaces can effectively suppress nanoparticle sintering, though partial encapsulation or charge‐transfer effects may, in some cases, reduce the available active surface [19]. Moreover, despite its stabilizing role, TiO_2_ itself has limited electrical conductivity [18] and an interfacial electronic structure (e.g., Schottky barriers) that may influence catalytic behavior [20]. Hybrid supports combining carbon and TiO_2_ aim to leverage the advantages of both: the conductivity of carbon and the stabilizing MSI of the oxide [21]. Recent studies suggest that decoupling the TiO_2_ layer from Pt by a thin carbon overlayer can yield improved stability without compromising electron transport [22].

Carbon spherogels represent a particularly attractive class of hybridizable and porous carbon supports. These materials consist of monodisperse, hollow carbon spheres (approximately 200 nm in diameter) arranged into a highly porous, electrically conductive aerogel network. Their morphology originates from a soft‐templating route using resorcinol–formaldehyde gels followed by supercritical drying, resulting in materials with tunable shell thickness, hierarchical porosity, and a well‐defined nanoscale geometry [23, 24, 25, 26, 27, 28]. Unlike conventional hollow carbon spheres produced via HF‐assisted hard templating [23], spherogels offer a simpler, HF‐free synthesis with precise control over structural parameters, making them promising supports for electrocatalysts. While they have been used in applications such as supercapacitors [29] and photocatalysis [30], their potential as HER catalyst supports has remained unexplored.

Complementary to advanced support architectures, controlling the deposition of Pt is equally important. Supercritical deposition (SCD) of Pt precursors enables highly uniform precursor adsorption, precise control over Pt loading, and narrow nanoparticle size distributions, even on delicate high‐surface‐area supports [14, 31, 32, 33, 34]. In SCD, a Pt precursor dissolved in supercritical CO_2_ infiltrates the porous support under conditions of very high diffusivity [31], followed by thermal or reductive conversion to yield dispersed Pt‐NPs [32]. Compared with liquid‐phase impregnation, SCD avoids solvent‐induced pore collapse and provides superior homogeneity and size control [31]. Previous studies demonstrated improved ORR stability on SCD‐prepared Pt/C electrocatalysts [31, 32, 33, 34] and the formation of narrowly dispersed Pt‐NPs on carbon aerogels with enhanced electrochemical properties [14, 35].

In this study, we introduce an integrated strategy that combines carbon spherogels with SCD to produce HER electrocatalysts with low Pt loading and high stability. Pure carbon spherogels are first examined to understand how hollow geometry, shell porosity, and nanoparticle dispersion influence HER activity. Hybrid TiO_2_/carbon spherogels are then used to isolate the effect of a TiO_2_ sublayer positioned beneath the carbon shell, allowing us to probe how MSI, electronic interactions, and local confinement jointly govern Pt stability. The resulting Pt/spherogel and Pt/TiO_2_/spherogel electrocatalysts are systematically compared with a wet impregnated sample and with conventional Pt/C of similar Pt loading. Structural characterization, including electron microscopy, XPS, and nitrogen sorption, is combined with electrochemical evaluation under RDE conditions in the 10–100 mA cm^−2^ range.

Finally, in contrast to many silica‐template routes, the soft‐templated spherogel synthesis used here enables HF‐free template removal during carbonization and is compatible with scalable CO_2_‐based drying and deposition processes. By integrating tunable nanoscale architecture with precise Pt loading control, the present work provides a model system for disentangling structure–property relationships in hollow carbon‐based electrocatalyst supports, offering insights relevant for the design of next‐generation HER electrocatalysts.

## 2 Results and Discussion

**FIGURE 1:**
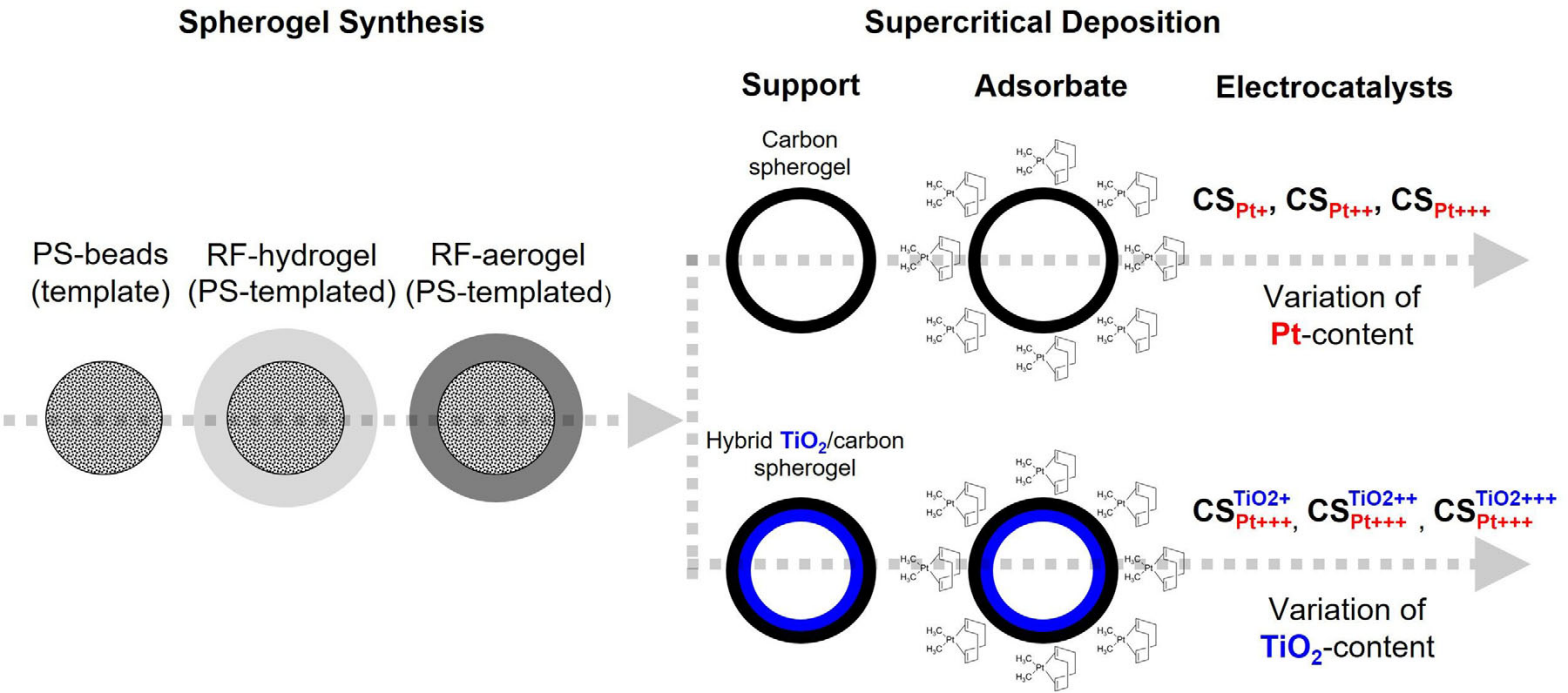
Scheme of stepwise material build‐up from RF‐template to CS and CS^TiO2^ supports followed by electrocatalyst generation via subsequent Pt‐NP decoration via SCD and thermal conversion.

In this study, six different electrocatalysts were synthesized: in a first step pure carbon spherogel (CS) and TiO_2_‐hybrid carbon spherogel (CS^TiO2^) supports were obtained from polystyrene (PS) templated resorcinol‐formaldehyde (RF) gels. In a second step all supports were decorated with Pt‐NPs via supercritical deposition (SCD), resulting in two different classes of electrocatalysts (CS_Pt_ and ${\mathrm{CS}}_{\mathrm{Pt}\;\;}^{\mathrm{TiO}2}$) (Figure 1). The synthesis of all materials is described in detail in the experimental section.

To isolate the effect of overall Pt‐loading and nanoparticle size on the electrocatalysts structure, HER‐performance, and stability, pure carbon spherogels (CS) were loaded with three different amounts of Pt (labeling: CS_Pt +_, CS_Pt + +_, CS_Pt + + +_). Furthermore, three hybrid spherogels with different amounts of the encapsulated TiO_2_ layer (CS^TiO2 +^, CS^TiO2 + +^, CS^TiO2 + + +^) were loaded with a fixed amount of Pt (${\mathrm{CS}}_{\mathrm{Pt}+++}^{\mathrm{TiO}2+}$, ${\mathrm{CS}}_{\mathrm{Pt}+++}^{\mathrm{TiO}2++}$, ${\mathrm{CS}}_{\mathrm{Pt}+++}^{\mathrm{TiO}2+++}$). The first part of this study deals with the (micro‐)structural characterization of all supports and electrocatalysts. In the second part, the electrocatalysts activity and stability in HER are assessed via tests with a rotating disc electrode (RDE) setup and in an acidic environment. The results are compared to a commercial benchmark electrocatalyst based on a carbon black support (C_Pt+++_: 10 wt% Pt on Vulcan XC‐72, by supplier information).

### 2.1 Physical and Chemical Characterization of Spherogel Supports and Electrocatalysts

**FIGURE 2:**
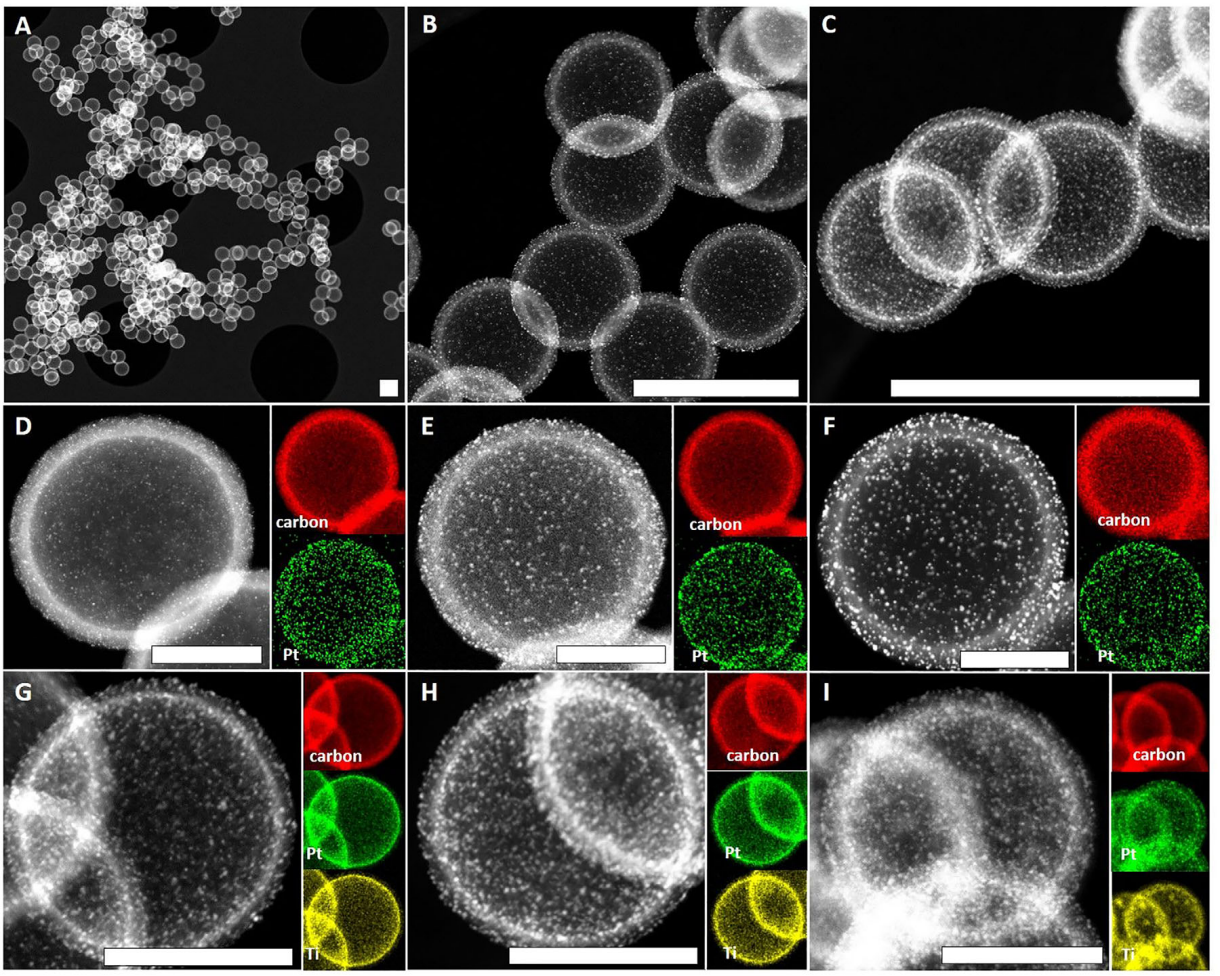
STEM HAADF images and EDX intensity maps of different spherogel‐electrocatalysts. Top, (A—C): Overview images taken at different magnifications, selected sample = **CS**_**Pt**++_. The scale bars in images (A—C) correspond to 300 nm. Middle, (D—F): Single sphere images of **CS**_**Pt**_‐electrocatalysts with different Pt‐loadings. (D) **CS**_**Pt**+_, (E) **CS**_**Pt**++_, (F) **CS**_**Pt**+++_. Bottom, (G—I): Single sphere images of ${\mathrm{CS}}_{\mathrm{Pt}+++}^{\mathrm{TiO}2}$‐electrocatalysts with different TiO_2_ content and constant Pt‐content. (G) ${\mathrm{CS}}_{\mathrm{Pt}+++}^{\mathrm{TiO}2+}$, (H) ${\mathrm{CS}}_{\mathrm{Pt}+++}^{\mathrm{TiO}2++}$, (I) ${\mathrm{CS}}_{\mathrm{Pt}+++}^{\mathrm{TiO}2+++}$. The scale bars in images (D—I) correspond to 100 nm. EDX color code: red = carbon, green = platinum, yellow = titanium.

Carbonization of RF/PS‐based polymer gels at 800°C with a dwell time of 2 h led in accordance with former works to conversion of RF into microporous carbon walls while simultaneously eliminating the inner PS template [25]. Dynamic light scattering (DLS) experiments of the used PS templating spheres showed a distinct monomodal size distribution, with slight batch‐to‐batch variation (Figure S1, 210 nm PS was used for pure carbon spherogels (CS) while 258 nm PS for the titania hybrid carbon spherogels CS^TiO2^). The inner diameter of CS shrank during pyrolysis by 8% to 237 ± 10 nm (initial PS diameter = 258 nm) and in case of hybrid CS^TiO2^ by 15% to 172 ± 5 nm (original PS diameter = 210 nm). All (hybrid)‐spherogel supports and electrocatalysts formed continuous networks of neatly shaped, monodisperse, highly spherical carbon hollow spheres (Figures 2A–C, and S2, S3). This network contained macropores being visible as: 1) larger voids between stacked spheres, 2) the inner part of each hollow sphere, which represents a single macropore. The destruction‐free removal of the PS templates during pyrolysis indicated the presence of wall‐interpenetrating pores, which enabled an effective mass transport (in this case: evaporating PS fragments) between the inner part and the outer surface of spheres through the transforming process. The final shell thickness S_Shell_ was estimated based on STEM images (Figure S3) and showed a significant difference between pure and hybrid spherogels (Figure S4, S_Shell_ ∼20.5 ± 2.4 nm in average; CS and S_Shell_ ∼13.0 ± 2.6 nm in average; CS^TiO2^). The slight differences in carbon shell thickness (excluding the TiO_2_ contribution) are attributed to variations in PS template size, since an equal amount of RF precursor was deposited on slightly different PS surface areas. Accordingly, the addition of the TiO_2_ precursor had no, or only a minor, influence on the shell thickness. Nitrogen physisorption measurements confirmed that the shell of CS‐supports contained mesopores with diameters being mainly in the range of approx. 3.5 to 5.5 nm (Figure S5A), while CS^TiO2^ based supports exhibited also a dominant presence of micropores (Figure S5B). These micro/mesopores offered significant specific surface area (SSA) in the range of 514–799 m^2^ g^−1^ (Table S1). While nitrogen adsorption data indicate microporosity in all samples, as evidenced by a pronounced steep uptake at low relative pressures (p/p_0_< 0.05; Figure S6), it should be noted that N_2_ cannot fully access ultramicropores (<∼0.7 nm). As a result, a portion of the microporous surface area remains “invisible” in N_2_ adsorption isotherms. Therefore additional micropore analysis was carried out via CO_2_‐physisorption, which confirmed the presence of micropores in a size range of ∼0.3–1.0 nm, providing an according overall SSA from 481–1006 m^2^ g^−1^ (Table S1, for micropore size distributions we refer to Figure S7) [36]. Notably, the SSAs derived from CO_2_ physisorption were in some cases higher than those obtained from N_2_‐physisorption. This apparent discrepancy originates from methodological differences: N_2_ at 77 K underestimates micropores due to kinetic restrictions, whereas CO_2_ at 273 K can access these pores more effectively. Thus, the CO_2_‐derived values represent complementary information on microporosity rather than a strict subset of the N_2_‐based total surface area. EDX‐measurements verified, that TiO_2_ formed homogeneously distributed, inner TiO_2_‐layers (Figure 2G–I, for the corresponding overlapped elemental maps we refer to Figure S8) whereas variation of the titanium‐precursor amount led to three distinct overall TiO_2_ loadings in the range of 16.7–26.7 wt% as confirmed by ICP‐OES analysis (Table S1). These results are in line with former works and demonstrate the robustness of the PS sphere soft‐templating approach [25, 28] Loading of the CS‐ and CS^TiO2^‐supports with different amounts of the platinum precursor Pt(cod)me_2_ via SCD and subsequent thermal conversion resulted then in two distinct classes of electrocatalysts (TiO_2_‐free and TiO_2_‐containing) with small Pt‐NPs being deposited and homogeneously distributed over the spherogel‐support (Figure 2B–I, for additional overview images including non‐loaded supports, we refer to: Figures S2, S3). No larger Pt aggregates and solely small Pt‐NPs were formed in all cases. Process and material related influences which led to these results are: a) the comparatively low temperature (400°C) used in the thermal conversion step leading to a rather low mobility of the Pt atoms on the supports surface during Pt‐NP formation (for temperature influence in the range of conversion temperature 200°C–1000°C we refer to [37]) and b) the porous nature of the carbon surface, which probably helped to prevent Pt‐NP migration toward each other during the thermal treatment [37].

**FIGURE 3:**
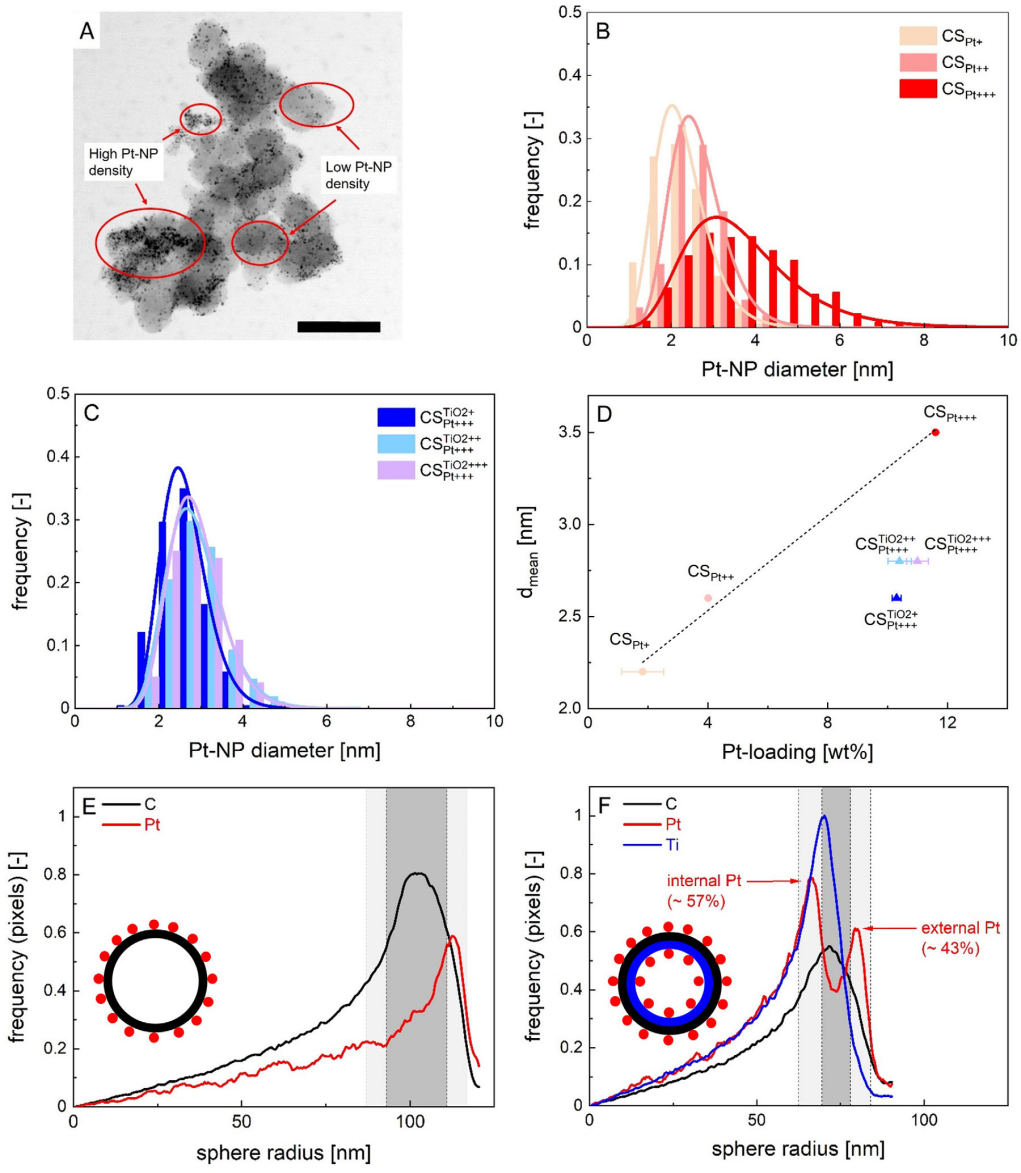
(A) STEM BF image of the commercial electrocatalyst C_+++_. The scale bar corresponds to 100 nm. (B,C) PSDs of carbon spherogel electrocatalysts (D) d_mean_ value of different electrocatalysts in dependence of the Pt‐loading. The dashed line represents a linear fitting of the CS_Pt_‐sample data. X‐error bars correspond to the standard deviation of elemental analysis. (E,F) radial elemental distributions of samples CS_Pt+++_ (E) and all ${\mathrm{CS}}_{\mathrm{Pt}+++}^{\mathrm{TiO}2+++}$(F).The grey areas represent the carbon wall thickness estimated from STEM images. Estimations were based on at least 8 different spheres. The brighter areas represent the standard deviations of averaged mean values (represented as darker areas). Insets represent schematic 2D cross‐sections of the according electrocatalysts.

In contrast to CS‐electrocatalysts, Pt‐NPs were not homogeneously distributed throughout the carbon surface in the commercial electrocatalyst: in particular, areas with increased Pt‐NP density were visible which contained Pt‐NPs in direct contact with each other (Figure 3A). It was therefore not possible to evaluate a representative Pt‐NP size distribution (PSD) for the C_Pt+++_‐sample. STEM‐HAADF images suggested that the largest population of Pt‐NPs was in the size range of approx. 2–5 nm, with Pt‐NPs/aggregates slightly exceeding these values being predominantly located in areas with higher Pt‐NP density. For CS‐electrocatalysts, representative PSDs could be derived from CS‐single sphere images (Figure 3B,C, *n* > 200 counts in each case) due to the homogeneity of the samples. In case of CS_Pt_‐electrocatalysts, the mean particle diameter of Pt‐NPs (*d_mean_*) was linearly related (*R^2^* = 0.98) to the overall Pt‐loading and precisely controllable in the range of d_mean_ = 2.2–3.5 nm by adjustment of the Pt‐precursor content during the SCD (Figure 3D). No significant variations of the PSDs and d_mean_ were observed for ${\mathrm{CS}}_{\mathrm{Pt}\;\;\;\;\;\;}^{\mathrm{TiO}2}$ samples with different TiO_2_ amount and constant Pt loading (Figure 3C,D, average d_mean_ = 2.7 ± 0.1 nm). Notably, Pt‐NPs were smaller and their size more narrowly distributed as compared to the CS_Pt + + +_ ‐electrocatalyst with similar Pt loading, which showed that the type of support influenced the final size of Pt‐NPs. The final Pt‐loading (Pt_load_) in all CS_Pt_‐samples was adjustable by setting the ratio between Pt‐precursor and amount of support during the SCD and covered a wide range (Figure 3D): CS_Pt +_  = 1.8 wt%, CS_Pt + +_ = 4.0 wt%, CS_Pt + + +_ and all ${\mathrm{CS}}_{\mathrm{Pt}+++}^{\mathrm{TiO}2}$ samples = 10.8 wt% (in average). Differences of the sphere radius and textural properties between CS‐ and CS^TiO2^‐supports had no significant effect on the overall Pt‐loading. The experimental error of the Pt loading (± 0.6 wt%, *n* = 4) was therefore calculated from the averaged value derived from all experiments with the highest Pt loading and regardless of the support type. Radial elemental distributions over the spheres diameter derived from EDX images (see Figures 2D–I, and S8) showed, that Pt‐NPs were solely located on top of the hollow carbon spheres external surface in CS_Pt_‐electrocatalysts (Figure 3E). Despite its porosity, no Pt‐NPs were present inside the shell, or in the inner part of the spheres.

By contrast, hybrid ${\mathrm{CS}}_{\mathrm{Pt}\;\;\;\;\;\;}^{\mathrm{TiO}2}$‐samples contained two different fractions of Pt‐NPs. While one fraction was attached to the outer side of the carbon shell surface (Pt‐NP_outs._), another was located on the inner shell surface within the spheres (Pt‐NP_ins._). This result demonstrates that part of the Pt‐precursor migrated through the shell, either during the SCD or thermal conversion steps. The strong affinity between TiO_2_ and Pt likely promoted this process, ultimately leading to the formation of Pt‐NPs in direct proximity of the metal oxide surface. An estimation based on the Pt signal intensity in the radial distribution analysis of elements reveals that a slightly higher fraction of Pt_ins._ (∼57%) was present compared to Pt‐NP_outs._ (∼43%, see Figure 3F). This corresponded to mass fractions of ∼4.6 wt% for Pt‐NP_outs._ and ∼6.2 wt% Pt_ins._. We surmise that the spatial separation of Pt fractions between the inner and outer shell surfaces ultimately resulted in smaller Pt‐NPs as compared to CS_Pt + + +_, as only a part of the total deposited Pt was available for NP formation in the distinct sections of the spherogel support. This hypothesis was corroborated by a theoretical estimation of the mean particle diameters d_mean_ of the Pt_ins._ and Pt‐NP_outs._ fractions, calculated based on their respective weight contributions using the linear correlation depicted in Figure 3D. The predicted d_mean_ values (2.6 nm for Pt‐NP_outs._ at 4.6 wt% and 2.8 nm for Pt‐NP_ins._ at 6.2 wt%) were in good agreement with the experimentally determined sizes observed in the ${\mathrm{CS}}_{\mathrm{Pt}\;\;\;\;\;\;}^{\mathrm{TiO}2}$‐samples.

**FIGURE 4:**
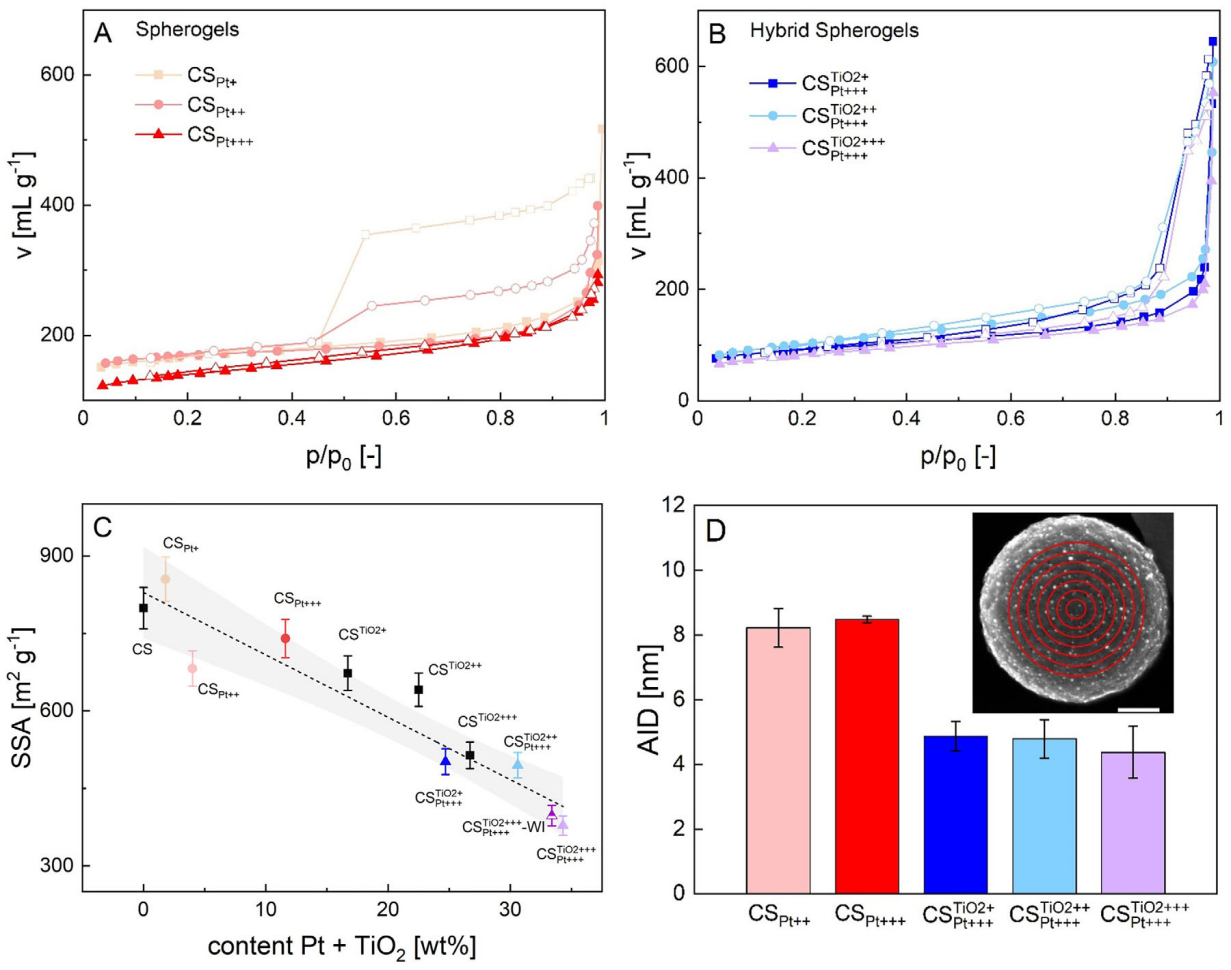
(A,B) Nitrogen sorption isotherms (77 K) of CS_Pt_ electrocatalysts and (B) ${\mathrm{CS}}_{\mathrm{Pt}\;}^{\mathrm{TiO}2}$ electrocatalysts. Adsorption cycle (solid symbols), desorption cycle (open symbols). (C) Specific surface area of all CS‐supports, electrocatalysts and the wet impregnated sample in dependence of the metal/metal oxide weight content. The dashed line represents linear fitting throughout bare supports and SCD loaded samples, the hatched area the 95% confidence interval. The Y‐error corresponds to the standard error of the method. (D) AID‐values of different electrocatalysts. Each column represents the average taken from different annular caps (inset: schematic example), the error bars correspond to the standard deviation (*n* = 7).

Insights about how the presence of TiO_2_ and loading with Pt influenced the pore structure and the specific surface area of the materials were provided by gas physisorption analysis. Nitrogen physisorption revealed type IV isotherms for CS_Pt_‐electrocatalysts, characterized by a pronounced hysteresis (H2a) due to cavitation (for CS_Pt +_ and CS_Pt + +_) during desorption through the micro‐ to mesoporous shell (Figure 4A) [36]. In contrast, a higher Pt content as present in sample CS_Pt + + +_ hindered the mentioned evacuation effect (no hysteresis visible). Similarly, in ${\mathrm{CS}}_{\mathrm{Pt}+++}^{\mathrm{TiO}2}$ samples the presence of titania affected nitrogen adsorption and desorption, leading to reduced micropore uptake and a hindered H2a hysteresis (Figure 4,B).

The SSA showed a decrease with the overall Pt and TiO_2_ content (Figure 4C). From a fundamental perspective, two main effects could explain this result: (1) the additional mass contribution from Pt and TiO_2_, and (2) the partial filling or blockage of carbon pores by the deposited Pt and TiO_2_ phases. Given the linearity of the trend across all materials (R^2^ = 0.9), we suggest that the reduction in SSA is primarily due to the added mass of Pt and TiO_2_, rather than to physical pore blockage or structural pore interactions. In this picture, the pore framework remained structurally intact after SCD and thermal conversion, which is consistent with previous studies of SCD on meso‐ to microporous carbon aerogels [14]. Furthermore, CO_2_‐physisorption measurements confirmed that all samples retained identical micropore size distributions (Figure S7).

Beyond aforementioned properties, the distance of Pt‐NPs between each other is of great practical importance: if Pt‐NPs are already intrinsically in direct contact after electrocatalyst synthesis or meet during electrocatalysis (due to migration), their coalescence—resulting in larger particles with reduced surface area—can occur relatively easy, leading then to a decline in electrocatalytic activity. As a rule of thumb, strong coalescence might be expected for average interparticle distances (AID) below 10 nm [38]. However, an adequate determination of the AID is not necessarily simple. When imaging techniques are used, the structural homogeneity of the support and the uniform distribution of Pt‐NPs are crucial to derive representative results. While these conditions were met for the CS‐electrocatalysts, this was not the case for the commercial sample, which is excluded from the following discussion. Notably, the high apparent particle density observed in STEM‐HAADF images (Figure 2B–I) arises from NPs being distributed across multiple layers within the nanometer‐thick shell, including those positioned at the back of the spheres. As a result, some particles that appear to be in close proximity may, in reality, be separated by significantly larger distances. The AIDs were therefore estimated based on secondary electron imaging (SEICOMP) and we only used the Pt‐NPs on the top surface of the sphere (which were sharp and in focus) for the calculation of the AIDs. (Figure S9). For ${\mathrm{CS}}_{\mathrm{Pt}+++}^{\mathrm{TiO}2}$‐samples, the results accounted only for the Pt‐NP_outs._‐fraction while the Pt‐NP_ins._‐fraction was neglected. In case of the CS_Pt+_ sample, the contrast between Pt‐NPs and the carbon surface was too low to allow for reliable evaluation. To ensure a clear individual identification and representative counting of Pt‐NPs, only the central regions of the spheres and areas without overlap from adjacent spheres were analyzed. To account for the 3D character of the images and the actual curvature of the spherogel surface, the area of each ring was calculated as annular spherical cap. The 2D projections of the half‐spheres were partitioned in ten, equally spaced concentric circles, and Pt‐NPs were counted in the seven inner sections, as particles increasingly overlapped beginning with section eight (example: Figure 4D, inset, for a detailed description of the evaluation and used images we refer to Figures S10–S13, and the according section in the supporting information).

The average interparticle distances were then calculated based on the assumption of a perfect spherical shape of the support and the simplification, that all Pt‐NPs featured the same mean diameter (thus neglecting the actual size distribution of the Pt‐NPs around the mean) according to Equation 1:

(1) $$\mathrm{AI}D_{\sec.}=\sqrt{\frac{4\cdot \eta \cdot A_{\sec.,\;i}}{\pi \cdot n_{i}}}-d_{\mathrm{mean}}$$

with A_sec.,i_ being the area of a concentric annular spherical cap with the particle count n_i_, AID_sec._ being the average interparticle distance of the evaluated section and η being the packing density as defined in Equation S6. No significant fluctuation of AID_sec._‐values were found across the inner seven sections (Figure S11), which verified the homogeneous distribution of Pt‐NPs throughout the supports surface. Individual values of each AID_sec._ were accordingly averaged to represent the overall AID in each sample. All samples featured low AIDs below 10 nm, whereas AIDs (8.4 nm) were higher in CS_Pt_‐electrocatalysts as compared to ${\mathrm{CS}}_{\mathrm{Pt}+++}^{\mathrm{TiO}2}$‐samples (4.4 nm) (Figure 4D). Key results of our analysis are that neither the d_mean_, nor the AID (and therefore the Pt‐NP distribution on the surface) were related to the SSA of the supports. The AID was also not related to the Pt‐loading as reflected by comparison of the CS_Pt + +_ and CS_Pt + + +_ samples. This finding contradicts observations reported in earlier studies: For instance, a rough estimation of the AID_calc._ purely based on geometrical considerations and in dependence of the SSA and Pt‐loading was proposed [38]:

(2) $$\mathrm{AI}D_{\mathrm{calc}.}=\sqrt{\frac{\pi }{3\sqrt{\pi }}}\cdot 10^{3}\cdot \rho_{\mathrm{Pt}}\cdot \left(\frac{100-Pt_{\mathrm{wt}}}{Pt_{\mathrm{wt}}}\right)\cdot \mathrm{SSA}\cdot d_{\mathrm{mean}}^{3}-d_{\mathrm{mean}}$$

where ρ_Pt_ is the density of Pt and Pt_wt_ is the Pt‐loading.

However, the prediction of the AID for our samples using Equation 2 resulted in a significant overestimation in comparison with the experimentally determined values (predicted AID_calc_ *_._* = 53–106 nm, Table S2). This was due to the proposed influence of the specific surface area SSA on the estimated AID: for instance, only low SSAs of merely ∼3–10 m^2^ g^−1^ would lead to the experimentally determined AIDs of 8.2 nm and 4.4 nm at a Pt‐loading of 10.8 wt% (as for samples in this work with max. Pt‐loading) according to Equation 2.

We emphasize that the role of SSA in Pt‐NP dispersion (and AID) must be considered with respect to the nature and accessibility of the surfaces available to the NPs, which are not accounted for in Equation 2. In the spherogel framework, three surface types can be distinguished: (I) the outer shell surface, (II) the inner shell surface facing the cavity, and (iii) the internal pore surfaces within the shell walls. Pt NPs were located on the outer shell surface (I) in CS_Pt_, and on both outer (I) and inner shell surfaces (II) in ${\mathrm{CS}}_{\mathrm{Pt}\;\;\;}^{\mathrm{TiO}2}$, while the pore surfaces (III) — which dominate the SSA — remained largely free of Pt. Consequently, the pore‐related SSA did not significantly contribute to AID or Pt dispersion. The quantitative contribution of the shell surfaces (I, II) to the SSA could however not be determined from the available data.

The primary reason for variations in AID between CS_Pt_ and ${\mathrm{CS}}_{\mathrm{Pt}\;\;\;\;\;\;}^{\mathrm{TiO}2}$‐electrocatalysts observed in our case could accordingly be rooted in the differences of the supports sphere sizes. However, since the supports used in this study differed not only in sphere size but also in composition, more in‐depth investigations—such as a systematic variation of the sphere radius within a single spherogel type—are required to gain a deeper understanding of how the AID can be controlled when SCD is used as a loading method.

In summary, Pt‐loading of spherogel supports via SCD resulted in precise Pt‐NP size control and highly homogeneous Pt‐NP dispersion throughout the individual spheres. However, SCD requires an additional high‐pressure post‐processing step, while standard Pt‐loading methods such as wet impregnation might be considered technically simpler. For direct comparison, we employed wet impregnation using the same support‐to‐Pt‐precursor ratio as in the production of the CS_Pt + + +_ and ${\mathrm{CS}}_{\mathrm{Pt}+++}^{\mathrm{TiO}2}$ electrocatalysts (for a detailed description of the wet impregnation method and results, see the SI and Figures S14 and S15). While wet impregnation likewise resulted in small Pt‐NPs, their dispersion was non‐homogeneous (Figure S15). The mesopore surface area of the resulting sample (${\mathrm{CS}}_{\mathrm{Pt}+++}^{\mathrm{TiO}2+++}$‐WI) was comparable to that of ${\mathrm{CS}}_{\mathrm{Pt}+++}^{\mathrm{TiO}2+++}$, indicating that the mesoporous framework of the spherogel support remained largely preserved during wet impregnation (SSA of ${\mathrm{CS}}_{\mathrm{Pt}+++}^{\mathrm{TiO}2+++}$‐WI = 397 vs 378 m^2^ g^−1^ in ${\mathrm{CS}}_{\mathrm{Pt}+++}^{\mathrm{TiO}2+++}$, Figure 4C). This observation indicates that capillary forces during solvent evaporation did not significantly affect the mesoporous framework of the spherogel support. Such structural robustness is advantageous for potential practical applications, for instance during the preparation of solvent‐based electrocatalyst inks used for electrode or membrane deposition. Summarized, the primary difference between the wet‐impregnated and SCD‐prepared samples lies in the spatial distribution of Pt‐NPs, which was clearly less homogeneous for the wet‐impregnated material. These results highlight the need to balance electrocatalyst quality against methodological complexity.

### 2.2 Electrochemical Performance and Stability of Electrocatalysts

**FIGURE 5:**
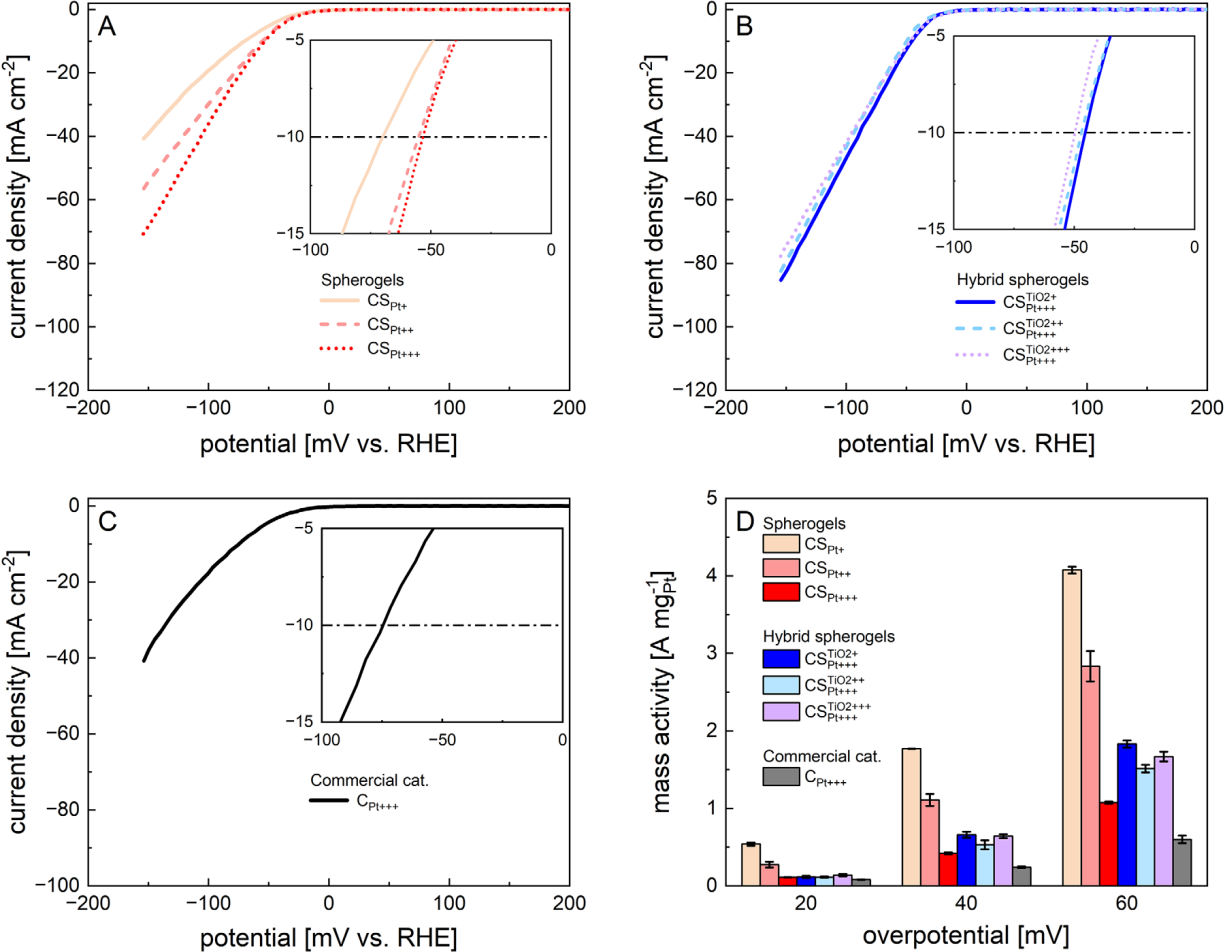
(A–C) Electrochemical characterization of electrocatalysts via LSV. Insets: magnified significant region of LSV analysis. A) CS_Pt_‐electrocatalysts with different Pt‐loading. (B) Hybrid ${\mathrm{CS}}_{\mathrm{Pt}+++}^{\mathrm{TiO}2}$‐electrocatalysts with different TiO_2_‐content and constant Pt‐loading. (C) The commercial CS_Pt+++_‐electrocatalyst. (D) Apparent Pt mass activities of all different electrocatalysts. Error bars correspond to the standard deviation of threefold determinations.

Linear sweep voltammetry (LSV) was performed on all synthesized electrocatalysts produced via SCD and the commercial C_Pt+++ _electrocatalyst as a benchmark (potential range: 152 to 200 mV vs. RHE, scan rate: 2 mV s^−1^) (Figure 5A–C). All self‐synthesized electrocatalysts exceeded the benchmark by demonstrating lower overpotentials at 10–40 mA cm^−2^ as compared to C_Pt+++_ (Table S3). In case of CS_Pt_ electrocatalysts, an increase in Pt‐loading led to a decrease in the overpotential at 10 mA cm^−2^ from 69.8 ± 0.4 to 52.8 ± 0.7 (Figure 5A), which is attributed to an increase in the number of Pt active sites. In order to study the effect of the TiO_2_‐loading on HER performance, the Pt loading was fixed at around 10 wt% in ${\mathrm{CS}}_{\mathrm{Pt}\;\;\;\;\;\;}^{\mathrm{TiO}2}$ samples. However, no significant effect of TiO_2_‐loading on overpotentials was detected (Figure 5B). Overall, the hybrid spherogel electrocatalysts exhibited substantially lower overpotentials across all tested current densities. At 10, 20, and 40 mA cm^−2^, the overpotentials were reduced by ∼13%–15% compared to CS_Pt + + +_, and by ∼34%–40% compared to the commercial C_Pt+++_ benchmark (Table S3).

From a practical point of view it is interesting to compare the specific Pt mass activities of different electrocatalysts. It is however noteworthy, that the build‐up of undissolved H_2_‐bubbles on the electrode surface limits H^+^‐diffusion toward the Pt active sites during the LSV measurements and interferes with the measurement [39, 40]. Due to this interference, we hereafter refer to the specific Pt mass activities as apparent Pt mass activities. CS_Pt_‐samples showed a gradual decrease of the apparent mass activity as the Pt loading increases (Figure 5D): this trend is most likely a result of mass transport limitations, and has been reported several times [16, 39, 41, 42]. Direct comparison of samples with the same Pt‐loading (${\mathrm{CS}}_{\mathrm{Pt}+++}^{\mathrm{TiO}2+}$, CS_Pt + + +_ and C_Pt+++)_ yields 1.6‐fold and threefold higher apparent mass activities of ${\mathrm{CS}}_{\mathrm{Pt}+++}^{\mathrm{TiO}2+}$ than CS_Pt + + +_ and C_Pt+++_, respectively (max. 0.66 A mg^−1^_Pt_ at 40 mV vs. RHE, Figure 5D). For a more rigorous and quantitative comparison of the three electrocatalysts, the potential window was extended to reach current densities above 100 mA cm^−2^, and all polarization curves were corrected for ohmic drop (iR) using the experimentally determined uncompensated resistance (R_s_ = 7.2 Ω) (Figure S16). The resulting iR‐compensated overpotentials at 10 and 100 mA cm^−2^ (η_10_ and η_100_) were 28.5 ± 0.3 and 49.3 ± 1.3 mV for ${\mathrm{CS}}_{\mathrm{Pt}+++}^{\mathrm{TiO}2+}$, 32.3 ± 2.2 and 99.4 ± 9.5 mV for CS_Pt + + +_, and 70.7 ± 4.8 and 167.6 ± 7.1 mV for C_Pt + + +_, respectively. These results clearly demonstrate the superior HER activity of the ${\mathrm{CS}}_{\mathrm{Pt}+++}^{\mathrm{TiO}2+}$ electrocatalyst, which exhibited significantly lower η_10_ and η_100_ values compared to both CS_Pt + + +_ and the commercial C_Pt + + +_ benchmark. Besides, to directly evaluate the influence of the Pt deposition method, a ${\mathrm{CS}}_{\mathrm{Pt}+++}^{\mathrm{TiO}2+++}$ electrocatalyst prepared via wet impregnation was additionally tested under identical HER conditions (Figure S17). This sample had higher overpotentials compared to the ${\mathrm{CS}}_{\mathrm{Pt}+++}^{\mathrm{TiO}2}$ electrocatalysts prepared via SCD (Table S3). For example, after iR correction, η_10_ and η_100_ were 42.0 ± 0.6 and 76.0 ± 0.8 mV, respectively, which are higher than those of the SCD‐prepared ${\mathrm{CS}}_{\mathrm{Pt}+++}^{\mathrm{TiO}2+}$ electrocatalyst (28.5 ± 0.3 and 49.3 ± 1.3 mV). The Tafel slope followed a consistent trend, where a lower Tafel slope indicates higher activity with values of 27, 34, and 41 mV dec^−1^ for ${\mathrm{CS}}_{\mathrm{Pt}+++}^{\mathrm{TiO}2+},\;CS_{\mathrm{Pt}+++}$ and C_Pt+++_, respectively (Table S3, Figure S18). It is important to note that while the Tafel slopes of ≈ 30 mV dec^−1^ observed here are consistent with kinetically controlled HER on Pt surfaces at low current densities, extracting intrinsic kinetic constants in acidic media remains challenging because mass transport limitations can set in rapidly, even at low overpotentials and very low Pt loadings (∼13 ng cm^−2^) [39, 40]. In this sense, the reported Tafel slopes should be regarded as apparent values that are useful for comparison with literature data, but not as absolute kinetic constants. This interpretation also reflects the increasing importance of mass transport effects at higher current densities. While the influence of Pt loading itself on the mass transfer limitation can be excluded in case of similar Pt‐loading, variations in the number of accessible active Pt sites—and consequently also in mass transport—are still to be expected due to differences in Pt‐NP sizes and support morphologies.

Abovementioned results indicated a superior performance of the ${\mathrm{CS}}_{\mathrm{Pt}+++}^{\mathrm{TiO}2}$‐electrocatalysts. Key factors affecting the electrocatalyst performance are linked to several aspects, which we introduce briefly in the following: 1) The number of accessible active sites, which depend on the size and morphology of Pt‐NPs as well as the nanostructure of the support. 2) Modifications in the electronic properties of the catalytic metal, influenced by factors such as variations in Pt‐NP‐support interactions (e.g. between particles in contact with carbon versus TiO_2_) or changes in the interparticle distance of the Pt‐NPs. 3) Mass transport inside the electrocatalysts matrix. In case of CS_Pt_‐electrocatalysts, the SCD method combined with the high surface area of the spherogel support led to a homogeneous distribution of highly dispersed Pt‐NPs on top of the external shell (in contrast to the commercial benchmark). This favorable Pt‐NP distribution and the macroporous spherogel network may create efficient pathways for the reactants and provide a high number of accessible Pt active sites for the HER, ultimately leading to higher current densities for the spherogel electrocatalysts compared to the commercial material. Although the hybrid electrocatalysts (${\mathrm{CS}}_{\mathrm{Pt}+++}^{\mathrm{TiO}2}$) contained approximately one half of their Pt‐NPs in the inner part of the spheres, they exhibited the highest apparent Pt mass activities when compared to electrocatalysts with equal Pt‐loading. Thus, it is indicated that the active sites of the internal Pt fraction were accessible for reactants and that sufficient exchange of educts and products through the porous shell was possible.

This interpretation is consistent with the pore size distributions obtained from N_2_ sorption (Figure S5), which show mean pore diameters of ∼5 nm for CS and ∼3 nm for CS^TiO2^, the latter exhibiting a tail toward ∼10 nm. Such pore dimensions are sufficiently large to avoid any geometric restriction for the transport of small electrochemical species (H_2_O, H^+^, dissolved gases). The relevant quantity for diffusive transport is therefore not the pore diameter but the effective diffusion length across the shell. Using τ ∼L^2^/D (τ =  characteristic diffusion time, L corresponding to the shell thickness of ∼10 – 20 nm, and D in the range of typical aqueous diffusion coefficients, D ∼ 10^−9^ m^2^ s^−1^), τ falls in the sub‐microsecond regime. Considering that HER turnover frequencies on Pt under acidic conditions typically lie in the 10^3^ – 10^5^ s^−1^ range, diffusive transport through the nanoscale spherogel shell is at least one to three orders of magnitude faster and therefore not rate‐limiting.

The direct comparison between CS_Pt + + +_ and ${\mathrm{CS}}_{\mathrm{Pt}+++\mathrm{TiO}2+}$ showed that the latter contained smaller Pt‐NPs, which might contribute to a higher utilization of the Pt‐moieties/increase of active sites. This was clearly evident from the cyclic voltammetry tests (Figures S19 and S20), which showed that ${\mathrm{CS}}_{\mathrm{Pt}+++}^{\mathrm{TiO}2+}$ exhibited discernible hydrogen adsorption features, which pointed toward a higher apparent electrochemical surface area as compared to CS_Pt + + +_ and C_Pt+++_. For the latter two samples, the hydrogen adsorption region could not be reliably identified, indicating that incorporation of TiO_2_ with Pt modified the apparent electrochemical surface area. In this context, a geometry‐based estimation of the accessible Pt surface area (see supporting information and Table S4) showed that Pt‐surface‐area differences were, however, insufficient to rationalize the observed activity and H‐UPD trends. Therefore it is likely, that further enhancement of HER performance was due to TiO_2_ incorporation, as has been reported in various studies, with the effect attributed to changes in the electron configuration of Pt induced by TiO_2_ [43, 44, 45, 46]. In our case, this was evident from the shifts in the binding energies of the Pt^0^ 4f and Pt^2+^ 4f peaks in the XPS analysis of ${\mathrm{CS}}_{\mathrm{Pt}+++}^{\mathrm{TiO}2+}$ (71.8 and 73.7 eV, respectively) compared to CS_Pt + + +_ (71.4 and 73.0 eV) (Figure S21). The Pt^0^ and Pt^2+^ peaks exhibited positive shifts of 0.4 and 0.7 eV, respectively, indicating electron transfer from Pt toward TiO_2_ – a clear sign of SMSI [47, 48, 49, 50]. The corresponding XPS survey spectra of CS, ${\mathrm{CS}}^{\mathrm{TiO}2+}$, CS_Pt + + +_ and ${\mathrm{CS}}_{\mathrm{Pt}+++}^{\mathrm{TiO}2+}$ confirm the presence of C, O, Ti, and Pt while no additional elements were detected (Figure S22A). In parallel, the Ti^4+^ 2p_3/2_ peak shifted slightly from 458.4 eV in ${\mathrm{CS}}^{\mathrm{TiO}2+}$ to 458.5 eV in ${\mathrm{CS}}_{\mathrm{Pt}+++}^{\mathrm{TiO}2+}$ (+0.10 eV; Table S5, Figure S22B). The absence of Ti^3+^ indicated that the Pt‐TiO_2_ interaction was through bridging oxygen species (Pt‐O‐Ti) [51, 52]. In the O 1s spectra, the lattice‐O peak (Ti‐O) remained fixed at ∼530.1 eV for ${\mathrm{CS}}^{\mathrm{TiO}2+}$ and ${\mathrm{CS}}_{\mathrm{Pt}+++}^{\mathrm{TiO}2+}$ (Figure S22C). A low‐intensity lattice‐O peak was observed in the CS sample, which can be considered a satellite peak. In ${\mathrm{CS}}_{\mathrm{Pt}+++}^{\mathrm{TiO}2+}$, the surface‐O and organic‐O (C─O/C═O) peaks shifted by +0.2 eV and +0.6 eV relative to CS^TiO2 +^, respectively, while showing shifts of +0.6 and +0.5 eV compared to CS_Pt + + +_. Interestingly, these peaks shifted by less than 0.1 eV relative to CS, reflecting a cooperative effect of Pt and TiO_2_. Moreover, the relative atomic percentage of surface‐O and lattice‐O within the O 1s spectrum changed significantly upon Pt deposition, from 19% and 54% in ${\mathrm{CS}}^{\mathrm{TiO}2+}$ to 50% and 25% in ${\mathrm{CS}}_{\mathrm{Pt}+++}^{\mathrm{TiO}2+}$, respectively (Table S6), highlighting the interfacial role of oxygen. In the C 1s spectra, the C–C peak at 284.5 eV remained the most intense across all samples, but its atomic percentage decreased upon incorporation of Pt and TiO_2_ (Figure S22D, and Table S6). Peaks at 283.6 and 283.9 eV in CS_Pt + + +_ and ${\mathrm{CS}}_{\mathrm{Pt}+++}^{\mathrm{TiO}2+}$, respectively, indicated Pt–C interactions [53]. Notably, in ${\mathrm{CS}}_{\mathrm{Pt}+++}^{\mathrm{TiO}2+}$, the C─O and C═O peaks shifted to lower binding energy by ∼0.8 and ∼2.4 eV, respectively, compared to CS. These shifts are larger than those observed for ${\mathrm{CS}}^{\mathrm{TiO}2+}$ or CS_Pt + + +_ individually and are consistent with contributions from both TiO_2_ and Pt incorporation, indicating a combined modification of the carbon surface electronic environment. To assess structural changes, Raman spectra were recorded and I_D_/I_G_ ratios extracted from the D and G bands (Figure S23 and Table S7). Both TiO_2_ and Pt individually increased I_D_/I_G_ (2.2 and 2.5, respectively) compared to CS (2.0), consistent with defect formation at the interface [54, 55]. Interestingly, ${\mathrm{CS}}_{\mathrm{Pt}+++}^{\mathrm{TiO}2+}$ showed a decreased I_D_/I_G_ ratio of 1.8, which can be attributed to Pt–TiO_2_ interactions occupying or masking defect sites, thereby lowering the apparent D‐band intensity [56]. Importantly, this trend is fully consistent with the XPS results, which revealed Pt–O–Ti linkages and a positive shift of the Pt 4f binding energies, indicating electronic redistribution at the interface. Together with the unchanged CO_2_ microporosity and intact morphology, these findings confirm that the carbon framework remained structurally preserved, while the local defect landscape is selectively modulated at the Pt–TiO_2_–C interface. Such interfacial electronic effects are expected to tune the Pt d‐band center, thereby optimizing the Gibbs free energy of hydrogen adsorption (ΔG_H_) for HER catalysis.

**FIGURE 6:**
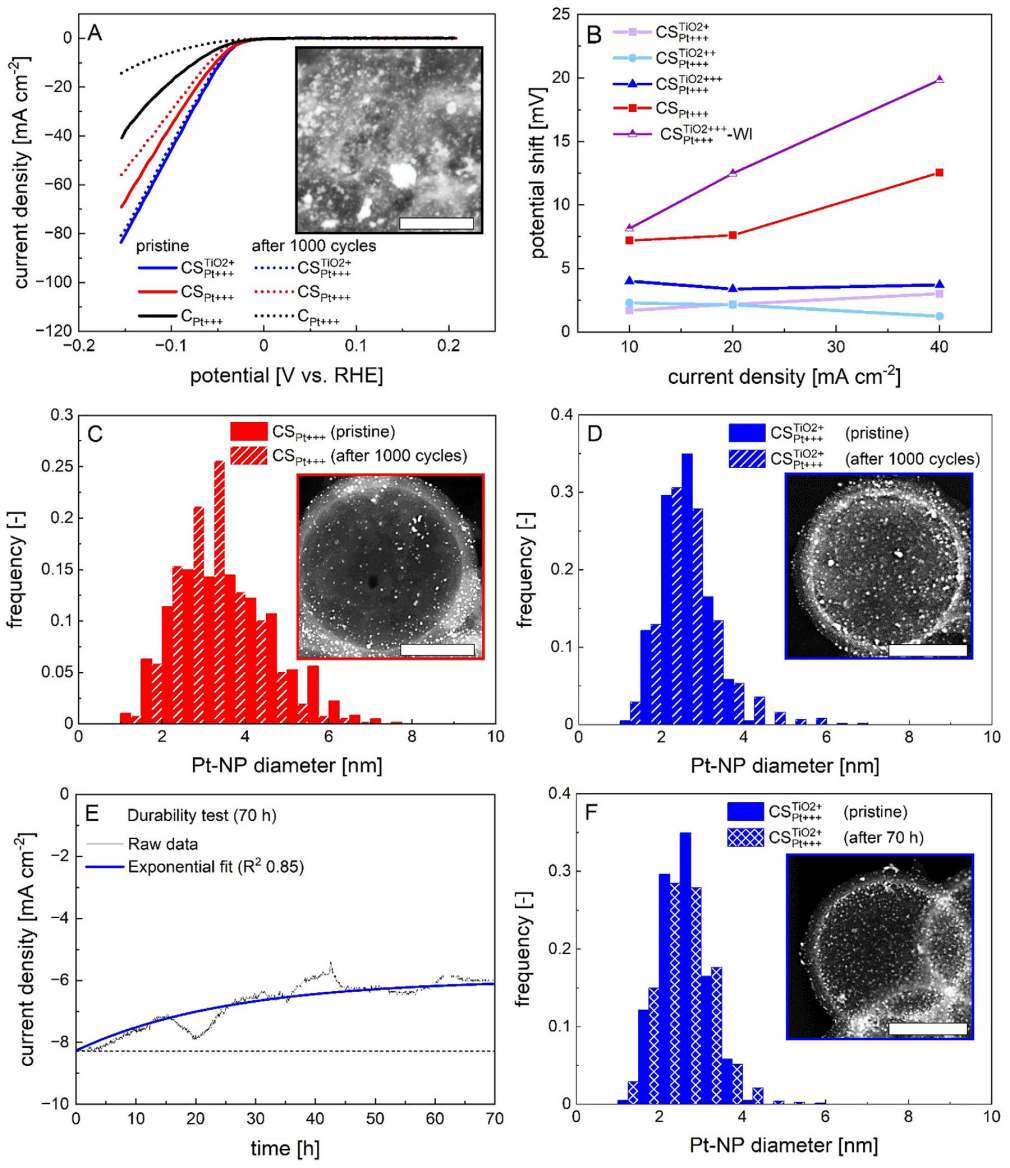
Electrochemical stability and post‐mortem analysis. (A) LSV test before and after ASTs (1000 cycles at 100 mV s^−1^) for two selected samples, (B) Potential shifts obtained after ASTs in dependence of the current density. Lines are drawn to guide the eye. (C) Pt‐NP size distributions after ASTs in comparison to PSDs of pristine samples. Insets: single sphere STEM‐HAADF images of samples after ASTs. The scale bars in (C), (D) and (F) correspond to 100 nm. (E) 70 h durability test of the sample ${\mathrm{CS}}_{\mathrm{Pt}+++}^{\mathrm{TiO}2+}$ via chronoamperometry. (F) Pt‐NP size distribution after the durability test in comparison to the PSD of the pristine sample. Inset: single sphere STEM‐HAADF image of the sample after the durability test.

The aforementioned results proved the overall high electrocatalytic activity of all spherogel‐based electrocatalysts and a positive contribution of TiO_2_. In order to assess the electrocatalysts stability, accelerated stability tests (AST) were carried out with the C_Pt + + +_, CS_Pt + + +_ and ${\mathrm{CS}}_{\mathrm{Pt}+++}^{\mathrm{TiO}2}$ electrocatalysts. These tests involved comparing the LSV of a sample in the initial experiment to the final LSV after 1000 cycles, with an increased scan rate of 100 mV s^−1^ between −50 and 200 mV vs. RHE. Significant deterioration observed for C_Pt + + +_ (52.3 mV at 10 mA cm^−2^, with no signal detected at higher current densities, Figure 6A) and for the CS_Pt + + +_ electrocatalyst (potential shifts of 7.5 to 13 mV at current densities between 10 and 40 mA cm^−2^, Figure 6A,B) pointed toward a substantial loss of electrochemical surface area during the tests.

In contrast, the TiO_2_‐containing spherogel electrocatalysts exhibited significantly lower potential shifts of ∼1–4 mV in the same current density range, demonstrating a substantial increase in stability, while no significant influence of variations in the TiO_2_ content was observed (Figure 6A,B, Figure S24). Despite the presence of TiO_2_, the electrocatalyst prepared via wet impregnation exhibited the lowest stability throughout all electrocatalysts (Figure S24). These findings suggest that the improved stability is generally closely related to the homogeneous Pt‐NPs distribution, which was most pronounced in samples prepared via SCD. Deterioration of the electrocatalyst performance results commonly from a complex interplay of primary and secondary degradation mechanisms occurring either simultaneously or sequentially [13, 38]. Hereby, Pt‐dissolution, Pt‐migration and carbon corrosion may act as primary mechanisms, which can then lead to secondary effects that progressively reduce the electrocatalyst's activity or structural integrity. Dissolution of Pt into the reaction fluid is expected to be especially severe for smaller Pt‐NPs which have a higher surface energy (Gibbs‐Thomson effect) [38]. Secondary effects resulting from Pt‐dissolution might be Ostwald ripening (due to redeposition of dissolved Pt on larger Pt‐NPs) and/or reformation of Pt‐NPs in the ionomer phase (which can therefore not contribute to catalysis) [38]. Besides Pt dissolution also Pt‐NP migration might occur, which leads subsequently to coalescence and an according growth of NPs. Corrosion of the carbon support is another primary degradation effect and results in a weakening between the support and Pt‐NPs. In case of severe carbon corrosion the supports structural integrity is lost, which might result in secondary effects like for example, Pt‐NP migration, agglomeration, coalescence and also in additional mass‐transport limitations for reactants [38]. Local corrosion of carbon in direct vicinity of Pt‐NPs promotes their migration and also detachment of whole Pt‐NPs [12, 38]. A strong indication for particle detachment from the carbon support is a reduction of the Pt‐NP density per area accompanied by only slight changes in the corresponding particle size distribution [13].

To evaluate possible reasons for the observed potential‐shifts, samples were collected from the tip of the glassy carbon disc after the ASTs and subjected to STEM post‐mortem analysis. Overview images, taken at lower magnification suggest that no severe carbon corrosion occurred, since the support's structure remained largely intact and no significant shrinkage/deformation of spheres was observed (Figure S25). This was expected, as 1) the electrolyte was oxygen free during electrochemical tests (due to purging with N_2_ for 30 min and maintaining the solution under an N_2_ blanket) and 2) carbon oxidation occurs generally at higher potentials [1 to 1.5 V vs RHE] [59] which are beyond the electrochemical test range applied in this study. The presence of some individual broken spheres (Figure S26) was most probably a result of the sample treatment in the recovery process/STEM sample preparation (e.g. mechanical stress). Images of single spheres taken at higher magnifications were used to determine post‐mortem Pt‐NP size distributions (Figure 6C,D). Although it is notable that the Pt‐NP distributions of pristine and post‐mortem electrocatalysts were taken from different individual samples, we assume that a sufficient comparability of results is given due to the high homogeneity of the Pt‐NP distribution throughout individual spheres and the defined geometrical features of the spherogel supports. Disappearance of the larger Pt‐NPs with sizes of approx. ≥ 5.0 nm was observed in case of the titania‐free CS_Pt + + +_ sample which led to a slight shift of the d_mean_ from 3.5 nm to 3.1 nm (Figure 6C). Furthermore, it was evident from STEM‐images that the number of Pt‐NPs per area decreased significantly after the AST (compare Figures 2F and 6C, inset). We exclude dissolution and Ostwald ripening as primary reasons for this result, since these processes would result in profound changes of the Pt‐NP size distribution, being reflected in a loss of smaller Pt‐NPs and—as opposed to our results—a corresponding shift toward a higher d_mean_. A plausible explanation for the disappearance of larger Pt‐NPs is their detachment from the outer surface of the carbon shell. Larger particles being more prone to detachment is a consequence of their lower adhesion to the carbon surface, since they exhibit a lower specific contact area to carbon as compared to smaller Pt‐NPs. While the confinement of Pt‐NPs in pores might principally help to prevent their detachment (and also their migration and coalescence [60]), a favorable size ratio between pores and Pt‐NPs is required in order to induce an effective stabilization: in case of too small pores, an efficient intrusion by Pt‐NPs is not possible, while too large pores provide no sufficient confinement. For instance, an optimum pore size ratio of ∼5 nm (pores) to 0.8 nm (Pt‐clusters) was reported to prevent coalescence [16]. However, our results showed that Pt‐NPs were not located in the inner porous part of the shell.

Only small changes of the Pt‐NP size distribution and no changes of the Pt‐NP‐density were detected for the ${\mathrm{CS}}_{\mathrm{Pt}+++}^{\mathrm{TiO}2+}$‐electrocatalyst after the AST (Figure 6D). In contrast to the CS_Pt + + +_ sample, the formation of some larger Pt‐NPs in the size range of 4.3–6.0 nm was observed. However, due to their low quantity, the d_mean_ remained unaffected. These results pointed toward the absence of Pt‐NP detachment and only minor Pt‐NP migration and coalescence, which is consistent with the low potential shift observed in the ASTs. Overall, the interaction between Pt and TiO_2_ (confirmed by XPS) significantly improved the stability of Pt‐NPs [48]. This interaction reduced the tendency of Pt‐NPs to migrate compared to TiO_2_‐free electrocatalyst, as evidenced by the PSDs obtained after the ASTs. Moreover, we surmise that both Pt‐NP fractions (Pt‐NP_outs._ and Pt‐NP_ins._) were stabilized by the presence of TiO_2_ and that this stabilization was sufficient to prevent the detachment of Pt‐NPs located on the outer shell surface. Considering the overall small Pt‐NP sizes and low AIDs of 4.4 nm, it is remarkable that the PSDs remained narrow and monodisperse in the ${\mathrm{CS}}_{\mathrm{Pt}+++}^{\mathrm{TiO}2+}$‐electrocatalyst even after the relatively harsh treatment.

In contrast to the CS‐based electrocatalysts, large Pt‐agglomerates (up to ∼250 nm in size) were observed in post‐mortem STEM images of the commercial material C_Pt+++_ (Figure 6A, inset, Figure S27). These findings showed that the Pt‐NPs experienced extensive coalescence which is consistent with the observed loss in catalytic activity. This coalescence was likely promoted by: (1) areas, where Pt‐NPs were in initial, direct contact with each other and (2) by the low surface (micro‐)porosity of the support which resulted in a higher mobility of Pt‐NPs on the carbon surface.

In order to assess the stability of the best performing electrocatalyst over a longer time period, a 70 h chronoamperometry test was carried out with the ${\mathrm{CS}}_{\mathrm{Pt}+++}^{\mathrm{TiO}2+}$ sample by tracking the loss of current density at a fixed potential (−40 mV vs. RHE). It should be noted that these measurements are highly sensitive to individual preparation steps [61], and a decrease in current density does not necessarily indicate catalyst degradation: various factors—such as changes in electrolyte composition, bubble accumulation, minor contaminations, or measurement‐related effects like sample detachment from the electrode surface—can significantly influence the results [61, 62, 63]. Apart from minor fluctuations, the course of the current density decreased slightly, following an exponential trend (Figure 6E). The absence of irreversible steps throughout the measurement indicated that no sudden electrocatalyst detachment (e.g. initiated by mechanical forces or significant carbon corrosion) occurred. CV measurements conducted before and after the stability and durability tests (Figure S28) showed nearly identical behavior, which indicated the absence of Pt dissolution or severe agglomeration, while EDX imaging of the used electrocatalyst confirmed the retention of the original materials (Figure S29). A final value of ∼6.0 mA cm^−2^ was reached after approx. 45 h, after which the system seemed to run in a steady state with a total loss of 2.2 mA cm^−2^ (overall 27%) in the current density. The PSD after 70 h showed no significant differences as compared to the PSD obtained before the AST (Figure 6F), which verified the long‐term stability of the system. Observed changes in the current density during the durability test can therefore most likely be attributed to other factors rather than to significant alterations in the (nano)‐structure of the electrocatalyst. Abovementioned results underscore the profound stability of the ${\mathrm{CS}}_{\mathrm{Pt}+++}^{\mathrm{TiO}2}$‐system over an extended period of 70 h.

### 2.3 Comparison of Spherogel Electrocatalysts With State of the Art

To place the performance of the spherogel‐based electrocatalysts into a broader context, we compared them along three dimensions: (1.) electrochemical stability, (2.) HER activity relative to commercial Pt/C benchmarks, and (3.) synthesis strategies of carbon hollow‐sphere‐based supports.

(1) A direct quantitative comparison of RDE stability data across studies is challenging, since Pt loading and experimental conditions vary. Nevertheless, potential shifts after 1000 cycles reported for different Pt‐based electrocatalysts span a range of 2–43 mV. Within this framework, the ${\mathrm{CS}}_{\mathrm{Pt}}^{\mathrm{TiO}2}$‐electrocatalysts produced here exhibit exceptionally low potential shifts, positioning them at the high end of stability compared to reported Pt‐based systems [14]. This includes outperforming carbon aerogels with higher surface areas up to ∼2500 m^2^ g^−1^, which showed significantly higher shifts (∼9.0 – 9.5 mV) and stronger Pt‐NP growth under identical experimental conditions [14].
(2) To benchmark HER activity, we compiled 14 recently reported Pt/carbon‐ and Pt/TiO_2_‐based electrocatalysts together with our best‐performing sample (Table S8). Since direct cross‐study comparisons are complicated by differences in electrode design and loading, each self‐synthesized electrocatalyst was evaluated relative to the respective commercial Pt/C benchmark reported in the same study (reflected by the difference in overpotentials). Within this consistent framework, ${\mathrm{CS}}_{\mathrm{Pt}+++}^{\mathrm{TiO}2+}$ combines competitive activity with high stability.
(3) Finally, Table 1 summarizes the production methods and structural features of carbon hollow‐sphere‐based supports. Compared to hard‐templating routes that often require HF etching and yield less homogeneous products, our soft‐templated approach integrates TiO_2_ incorporation in a one‐pot step and avoids hazardous etchants. The resulting uniform hollow spheres provide a robust platform for stabilizing ultrasmall Pt‐NPs.

**TABLE 1:** Properties and production methods of various carbon hollow‐sphere‐based electrocatalysts.

Material^a)^	Source	Pt_load_	d_mean_	SSA	Production	Application
		(wt%)	(nm)	(m^2^ g^−1^)	method	
CS_Pt + + +_	This work	11.6	3.5	799	Soft templating on PS‐spheres	HER
${\mathrm{CS}}_{\mathrm{Pt}+++}^{\mathrm{TiO}2+}$	This work	10.3	2.6	673	Soft templating on PS‐spheres	HER
Pt_5_/HMCS	[16]	5.08	∼0.8	1534^b)^	Hard templating, via HF etching	HER
Pt@HGS	[38]	20	1.0–4.0	∼1200^b)^	Hard templating, via HF etching	Fuel Cells
HGS	[15]	20	3.0–4.0	∼1200^b)^	Hard templating, via HF etching	Fuel Cells
Pt/rGO/TiO_2_	[22]	20	∼4.8	∼40^b)^	Soft templating on PS‐Spheres	Fuel Cells

^a)^ Abbreviations are given according to the sample assignment in the respective studies.

^b)^ examined via nitrogen physisorption, BET method.

One important quality criterion of the catalyst support is its morphological uniformity. In this work, we consistently obtained well‐defined, intact Pt‐loaded hollow spheres, as confirmed by comprehensive STEM studies, whereas several previous reports only showed isolated examples of intact structures [15, 38]. Moreover, TiO_2_ was homogeneously encapsulated in the carbon matrix, yielding highly uniform hybrid spheres. For comparison, Pt/rGO/TiO_2_ featured a much lower degree of morphological control and structural integrity [22]. By adjusting the template size and concentration, the hollow sphere diameter and shell thickness can be precisely tuned [25]. Post‐synthetic Pt deposition via SCD further ensured high control over Pt loading and avoided precursor‐derived residues (for instance sulfur in Pt_5_/HMCS) [16]. In contrast to our materials, Pt‐NPs were directly incorporated in the mesoporous network inside the shell in other works [15, 16, 38]. In our case, SCD resulted, however, in the major population of Pt‐NPs being located at the external surfaces: hereby, the presence of TiO_2_ as a sublayer seemed to be sufficient to provide pronounced stabilization for both Pt‐NP fractions (Pt‐NP_outs._ and Pt‐NP_ins._).

Taken together, these comparisons highlight that ${\mathrm{CS}}_{\mathrm{Pt}}^{\mathrm{TiO}2}$‐electrocatalysts combine high activity, high stability, and a scalable, etching‐free synthesis.

## 3 Conclusions

In this work, we present a HF‐free synthesis route for the development of highly active and stable HER electrocatalysts by combining the unique properties of carbon spherogels for the first time with the supercritical deposition technique. This combination enables precise control over (a) the morphology of the support and (b) the Pt loading, as well as the Pt‐NP size. The homogeneous distribution of small Pt‐NPs on the hollow sphere surfaces resulted in significantly enhanced apparent mass activities compared to a commercial reference with a similar Pt loading (∼10 wt%). One key finding of this study is that the incorporation of TiO_2_ as a sublayer significantly improved the electrocatalyst stability, effectively preventing Pt detachment and growth—despite the small Pt‐NP sizes and low interparticle distances. From a synthesis perspective, our approach for producing hollow carbon spheres offers the advantage of a straightforward and scalable, thermally triggered template removal during the carbonization step, eliminating the need for hydrofluoric acid which is typically required in conventional silica‐templating methods. Furthermore, a comparative experiment using wet impregnation showed that the mesoporous framework of the spherogel support remains largely preserved during solvent‐based processing, indicating a high structural robustness of the material. This demonstrates that the superior stability of the SCD‐prepared samples mainly arises from the more homogeneous Pt‐NP dispersion rather than differences in the pore structure. Taken together, the combination of structural robustness, high durability, and relatively simple synthesis suggests that such systems could contribute to improving the cost efficiency of electrochemical green hydrogen production.

Due to their well‐defined geometrical shape and high structural homogeneity, spherogel electrocatalysts are particularly well‐suited as model systems for the systematic investigation of structure‐property relationships. For example, the overall spherogel diameter can be varied while keeping all other parameters constant, allowing for the quantification of macroporosity effects and mass transport throughout the sphere network. Other tunable properties include the shell thickness, the pore‐to‐Pt‐NP size ratio, and interparticle distance variations. Due to high intrinsic activity of spherogel electrocatalysts under standard RDE benchmarks, it should be noted that high‐current durability (>100 mA cm^−2^) could not be meaningfully assessed in the RDE due to mass‐transport and bubble limitations: Such testing requires Membrane Electrode Assembly (MEA) or Gas Diffusion Electrode (GDE) platforms and will be the subject of future investigations. Accordingly, the limitations of the current dataset should be noted: the present study does not yet include high current‐density stress testing, which should be explicitly addressed in future work. Beyond this, the transferability of the developed spherogel electrocatalysts to practical electrolytic cell environments (e.g., PEM or AEM electrolyzers) will require dedicated MEA or GDE studies assessing performance, stability, mass‐transport behavior, and catalyst utilization under device‐relevant conditions, which is beyond the scope of this study. These conditions differ fundamentally from the present RDE setup in terms of reactant supply, operating pressure, membrane‐coupled transport, and local current density distribution.

At this stage, we believe that the materials presented in this work can contribute to the rational design of next‐generation electrocatalysts with reduced Pt‐loading. Furthermore the SCD approach employed here may in principle be extended to the deposition of various metals, offering opportunities to explore spherogel‐supported catalysts beyond Pt including Cu, Co, Ni, Mo, Pd, and Rh. While we focused on HER in this study, ${\mathrm{CS}}_{\mathrm{Pt}}^{\mathrm{TiO}2}$‐systems also appear promising for other applications, such as the oxygen reduction reaction or selective dehydrogenation as supported by supplementary proof‐of‐principle experiments (see the corresponding section in the supporting information and Figure S30).

## 4 Experimental Section

### 4.1 Chemicals

Technical grade acetone (>99%) was purchased from VWR. Styrene (≥99%), titanium(IV)bis(ammonium lactate) dihydroxide solution (50 wt% in H_2_O), polyvinylpyrrolidone (average molar weight 40 000), resorcinol (99%), formaldehyde solution (37% stabilized with 10% methanol), sodium carbonate, (1,5‐cyclooctadiene)dimethylplatinum(II) (Pt(cod)me_2_), (>97%) and sulfuric acid (95%–98%) were acquired from Sigma Aldrich. Potassium persulfate was supplied by Honeywell Fluka. Carbon dioxide 5.0 and nitrogen 5.0 were procured from Westfalen AG. Nafion117 solution at a 5% concentration was sourced from Quin‐tech. All chemicals were used without further purification. A commercial Pt/C electrocatalyst *C‐PT‐10* containing nominally 10 wt% Pt supported on Vulcan XC‐72 was acquired from Sigma Aldrich. Distilled and deionized water with a resistance of 18.2 MΩ was used in the experiments.

### 4.2 Synthesis of Pure and Hybrid Carbon Spherogels

**FIGURE 7:**
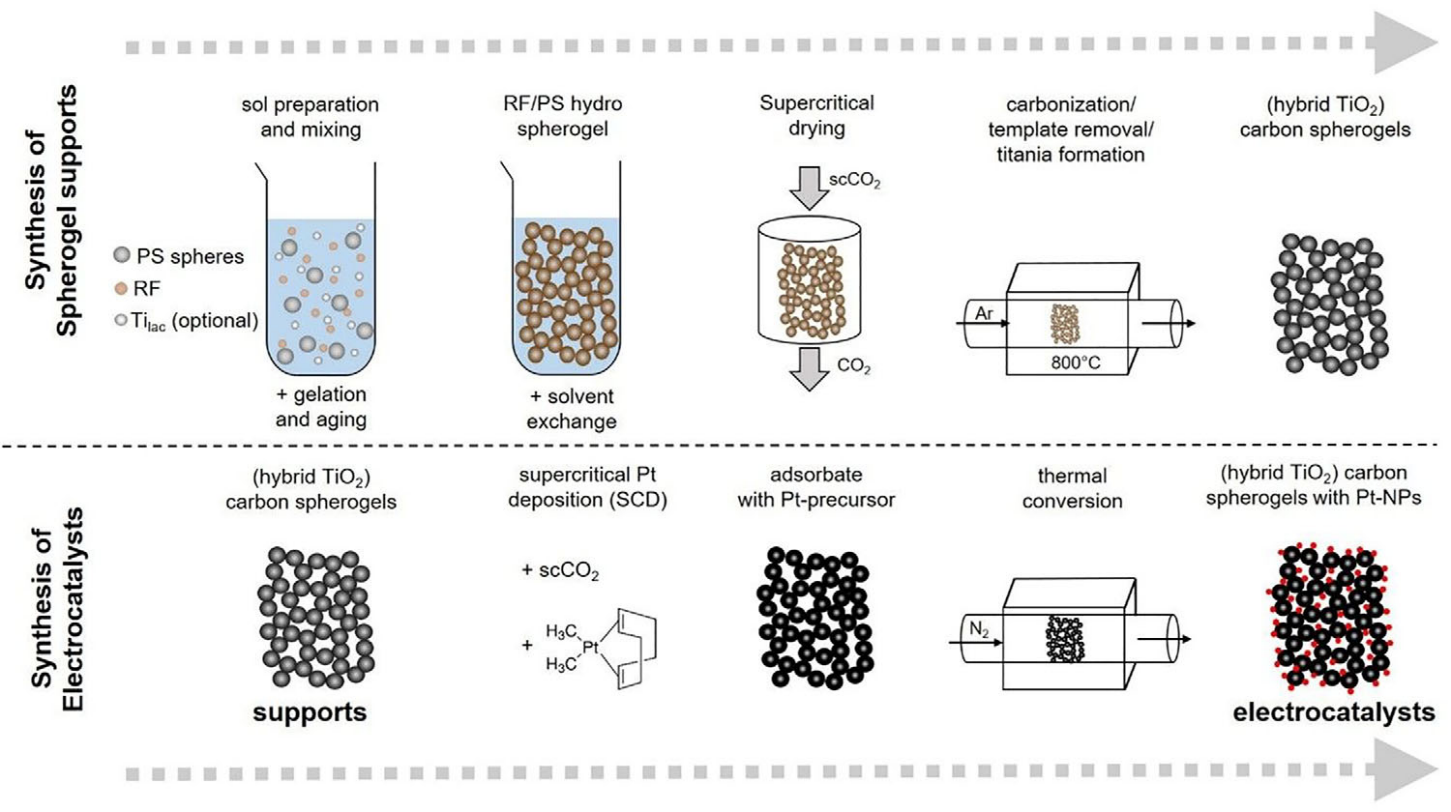
Production scheme for synthesis of spherogel supports via soft‐templating of RF‐gels (top) and electrocatalysts via supercritical deposition of Pt(cod)me_2_ (bottom).

An overview of carbon and hybrid carbon spherogel generation is provided in Figure 7, top part. Spherical templates in form of polystyrene (PS) beads were synthesized by emulsion polymerization of styrene with potassium persulfate as initiator and polyvinylpyrrolidone as stabilizer as described elsewhere [64]. The PS sphere solution was centrifuged, washed three times with water and finally diluted to a concentration of 9 wt%. For each synthesis approach 25 g of solution were mixed with 1.25 g resorcinol (R) under mild magnetic stirring at room temperature for 10 min. For the case of hybrid carbon/TiO_2_ spherogels, the titanium precursor titanium(IV)bis(ammonium lactate)‐dihydroxide (Ti_lac_) was added dropwise to the solution under stirring for an additional 10 min. To tailor hybrid spherogels with varying TiO_2_‐content, three different amounts of Ti_lac_ (1.2 g, 2.5 g, and 3.7 g) were used in individual syntheses. The crosslinking reaction was initiated via addition of formaldehyde (F) (molar ratio of F/R = 1.5) and 0.024 g NaCO_3_. The pH value was adjusted to 3 by the addition of 2M HNO_3_ and the solution was stirred for an additional hour. The final sols were transferred to cylindrical glass vials, which were sealed and placed in an oven for gel formation and aging at 80°C for 7 days.

After aging, the original solvent (H_2_O) was extracted by immersing the gels in acetone. To remove any unreacted species and by‐products, fresh acetone (100 mL) was added every 24 h and replaced three times. The wet gels were transferred to an autoclave and dried via supercritical extraction with CO_2_ at 60°C and 110 bar. Finally, the obtained PS‐templated, organic aerogels were carbonized in a tube furnace (alumina tube) under controlled Ar atmosphere (75 NL h^−1^) at 800°C with a heating ramp of 60°C h^−1^, and a dwell time of 2 h. Upon cooling at room temperature, monolithic carbon spherogels samples were obtained.

### 4.3 Platinum‐Nanoparticle Decoration

The supercritical deposition method (SCD, Figure 7, bottom part) was chosen over conventional wet impregnation for Pt loading of the pure carbon and hybrid spherogel supports, as it has consistently been reported to yield narrower Pt particle size distributions and higher dispersion [50, 51, 52, 53]. First, samples were degassed under vacuum (<7 mbar) at 60°C for at least 24 h to remove moisture. Then 140 mg of sample was filled in a cellulose teabag, which was enclosed and then carefully placed inside a cylindrical, high‐pressure stainless‐steel autoclave (45 mL volume). The teabag rested on a stainless‐steel grid, holding it in place above a magnetic stirrer. Finally, 15, 30 or 60 mg of the precursor Pt(cod)me_2_ were added to the autoclave before sealing it. A syringe pump (Teledyne Isco, 550 D) was used to fill the autoclave with CO_2_ until a total pressure of 160 bar was reached. The pump was supplied with cooled CO_2_ at 0°C (refrigeration circulator: Haake, C35P) and cooled itself, to allow a pressurization of the autoclave to 160 bar by a single stroke of the pump. Before entering the autoclave, the CO_2_ was reheated to 80°C (circulating thermostat: Haake, N3). The mixture was stirred for 72 h at 80°C. The temperature in the autoclave was controlled by an external electric heating mantle coupled to an internal Pt100 sensor. After the impregnation phase, the autoclave was depressurized by gradually opening a manual check valve at a rate of 7 bar min^−1^ to prevent crystallization of Pt(cod)me_2_. After depressurization, the adsorbate (spherogel with adsorbed Pt(cod)me_2_) was retrieved from the teabag and transferred to a thermogravimetric scale (Linseis, STA PT1600) for thermal conversion of Pt(cod)me_2_. Adsorbed Pt(cod)me_2_ was converted to Pt‐NPs at 400°C for 4 h under constant N_2_ stream (100 cm^3^ min^−1^).

### 4.4 Materials Characterization

Dynamic light scattering experiments of the initial (template) PS colloidal solutions were recorded on a Malvern Zetasizer instrument using a light‐backscattering angle of 173°. One measurement consisted of three times thirty separate sub‐measurements. Textural characterization of supports and electrocatalysts was carried out via gas physisorption using nitrogen (quality 5.0) at 77 K (micro/mesopores) and carbon dioxide (quality 5.0) at 273.15 K (micropores). Constant temperatures during measurements were ensured by submerging the samples in liquid nitrogen (N_2_ physisorption, 77 K) or water crushed‐ice mixtures (CO_2_ physisorption, 273 K). An overall sample mass of approx. 20 mg was used in each run and all samples were degassed at 100°C under vacuum (<7 mbar) for at least 6 h prior to each analysis. The respective isotherms were determined with a gas sorption analyzer (Nova 4200e, Quantachrome) by a manometric method from 5 mbar to ambient pressure. The specific surface area, pore volume, and pore size distributions of the materials were derived by quenched‐solid density function theory (QSDFT) methods (kernel: equilibrium isotherm, cylindrical pores) for N_2_ isotherms and non‐local density function theory (NLDFT) for CO_2_ isotherms. All gas sorption experiments were carried out as single determinations with an estimated relative measurement error of 5%.

For the determination and quantification of the elements an inductively coupled optical emission spectrometer (ICP‐OES, Optima 8300 DV, Perkin Elmer) was used. Samples were first converted to a fine aerosol and then brought to an excited state via a superheated plasma of 6000 to 10 000 K. For quantification, the detected intensities of the elements of interest were compared to those of standards with given concentrations.

Surface chemistry analysis was performed using X‐ray photoelectron spectroscopy (XPS) with a Thermo‐Scientific K‐Alpha spectrometer with an Al anode (Al Kα = 1468.3 eV). The electron take‐off angle was maintained at 90° relative to the sample surface and the analyzer lens axis. Calibration of all binding energies was carried out using the C 1s peak at 284.5 eV. XPS peak fitting was performed using XPS Avantage 5.9 software. An Avantes AvaSpec‐ULS‐TEC‐EVO system was a wavelength of 785 nm was used for characterization of ID/IG ratios of both the pristine and Pt loaded supports. Scanning transmission electron microscopy (STEM) data was acquired using a JEOL JEM‐F200 transmission electron microscope operating at 200 kV equipped with a cold field emission electron source and a large windowless JEOL Centurio EDX (Energy Dispersive X‐ray emission) detector (100 mm^2^, 0.97 srad, energy resolution < 133 eV @ MnKα) for local composition analysis. Bright field (BF) and high‐angle annular dark‐field (HAADF) images showing z‐contrast and secondary electron images (SEI) showing the surface topology as well as EDX intensity maps were acquired using a beam current of 0.1 nA and a beam diameter of 0.16 nm. The radial distribution of the elements was done using the Pasad plug in [65] in Digital Micrograph.

### 4.5 Electrochemical Characterization

Electrochemical characterization of the samples was performed by employing a conventional three‐electrode cell connected to a potentiostat, (Pine Instrument Company Model AFCBP1). The working electrode, provided by Pine Instruments, consisted of a glassy carbon disk with an internal diameter of 0.5 cm (surface area of 0.1963 cm^2^). An Ag/AgCl reference electrode from Gamry Instruments was filled with a saturated KCl solution and a Pt wire was used as the counter electrode. The electrolyte was a 0.5 M H_2_SO_4_ solution. Nitrogen was used to purge the solution and for blanketing during the measurements. A catalyst ink was prepared by dispersing 8 mg of the catalyst with 1 mL of 1,2‐propanol, 3 mL of distilled water, and 10 µL of a 20 wt% Nafion solution from Ion Solutions Inc. The mixture was sonicated in an ultrasonic water bath for 1 h before deposition. Subsequently, 10 µL of the prepared ink was deposited onto the glassy carbon disk, for a electrocatalyst loading of about 100 µg cm^−2^ and allowed to air dry at ambient temperature and pressure overnight. We conducted linear scanning voltammetry (LSV) at a scan rate of 2 mV s^−1^, and all reported potentials were corrected for RHE (Reversible Hydrogen Electrode) according to Equation 3. iR compensation was applied where indicated for selected electrocatalysts in order to enable a more rigorous comparison of HER activity.

(3) $$E_{\mathrm{vs}.\;\mathrm{RHE}}=E_{\mathrm{vs}.\;\mathrm{Ag}/\mathrm{AgCl}}+E_{\mathrm{vs}.\;\mathrm{Ag}/\mathrm{AgCl}}^{\theta }+0.059\cdot \mathrm{pH}$$

## Author Contributions

The manuscript was written through contributions of all authors. All authors have given approval to the final version of the manuscript.

## Conflicts of Interest

The authors declare no conflict of interest.

## Supporting information




**Supporting File**: smll73187‐sup‐0001‐SuppMat.docx.

## Data Availability

The data that support the findings of this study are available in the supplementary material of this article
