# Supplementary material for: Titanium Dioxide Induced Immobilization of Small Platinum Nanoparticles on Carbon Spherogels: A Strategy Towards Stable and Efficient Electrocatalysts for the Hydrogen Evolution Reaction
Source: Small. 2026 Mar 26;22(28):e12049. doi: 10.1002/smll.202512049 (PMC13181522; doi:10.1002/smll.202512049)
Supplement: Supplementary file 1 — Supporting File: smll73187‐sup‐0001‐SuppMat.docx. [file SMLL-22-e12049-s001.docx]

*Supporting Information for:*

Titanium dioxide induced immobilization of small platinum nanoparticles on carbon spherogels: a strategy towards stable and efficient electrocatalysts for the hydrogen evolution reaction

Philip S. Pein ^1^, Ala Alsuhile ^2,3^, Simon Penner ^4^, Asghar Mohammadi ^4^, Gregor A. Zickler ^5^, Aseel Almohammad ^5^, Finn Baron ^1^_‚_ Mathis Kirstein ^1,6^, Can Erkey ^2,3^, Irina Smirnova ^1,6^, Michael S. Elsaesser ^5^ *, Baldur Schroeter ^1,6^ *

*^1^ Institute of Thermal Separation Processes, Hamburg University of Technology, 21073, Hamburg, Germany*

^2^ *Chemical and Biological Engineering Department, Koç University, 34450, Istanbul, Turkey*

*^3^ Koç University Hydrogen Technologies Center (KUHyTech), Istanbul, Turkey*

*^4^ Institute of Physical Chemistry, University of Innsbruck, 6020 Innsbruck, Austria*

*^5^ Department Chemistry and Physics of Materials, University of Salzburg, 5020 Salzburg, Austria*

*^6^ United Nations University Hub on Engineering to Face Climate Change at the Hamburg University of Technology, United Nations University Institute for Water, Environment and Health (UNU-INWEH), Hamburg, Germany*

* Corresponding authors: michael.elsaesser@plus.ac.at (ME), baldur.schroeter@tuhh.de (BS)


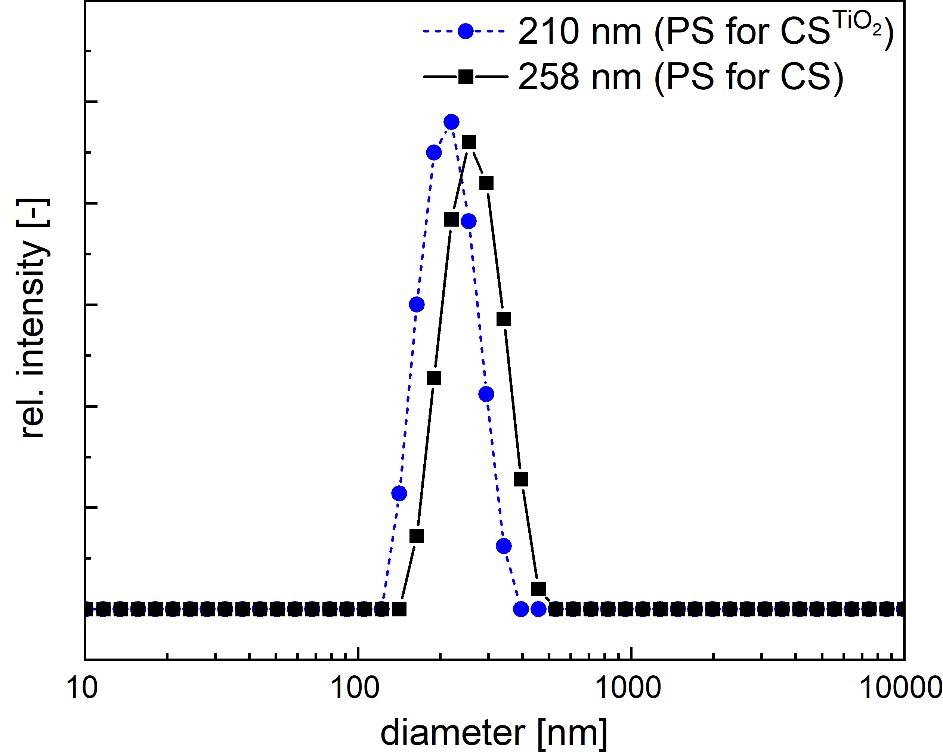


**Figure S1** Size distributions of polystyrene spheres used for templating of CS (solid squares, black) and $\mathrm{CS}^{TiO2}$ (solid circles, blue), determined via dynamic light scattering.


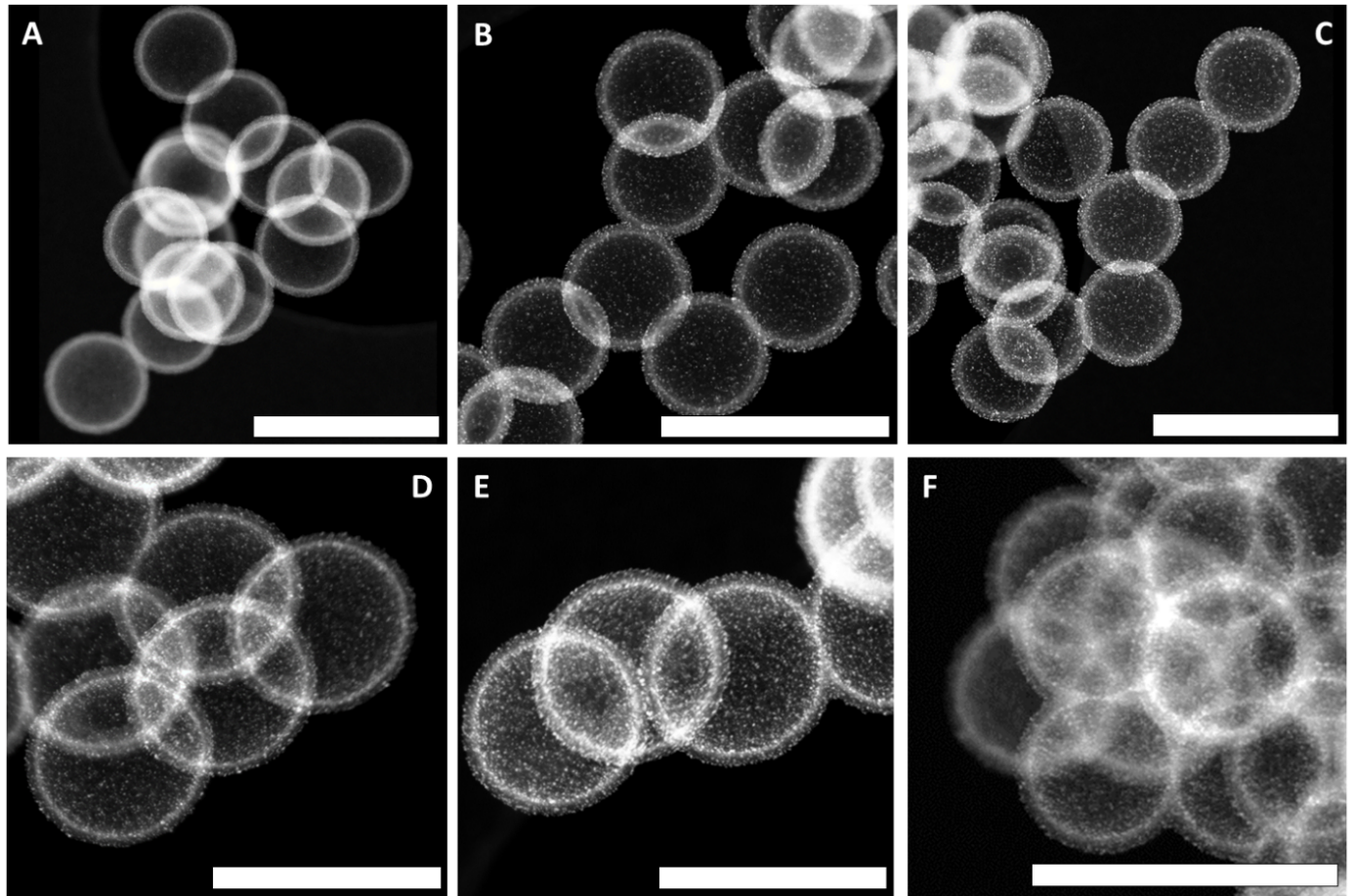


Figure S2 STEM HAADF images of CS-electrocatalysts. A) $\mathbf{CS}_{\mathbf{Pt+}}$, B) $\mathbf{CS}_{\mathbf{Pt++}}$,C) $\mathbf{CS}_{\mathbf{Pt+++}}$, D) $\mathbf{CS}_{\mathbf{Pt +++}}^{\mathbf{TiO2+}}$, E) $\mathbf{CS}_{\mathbf{Pt +++}}^{\mathbf{TiO2++}}$, F) $\mathbf{CS}_{\mathbf{Pt +++}}^{\mathbf{TiO2+++}}$. The scale bars correspond to 400 nm.


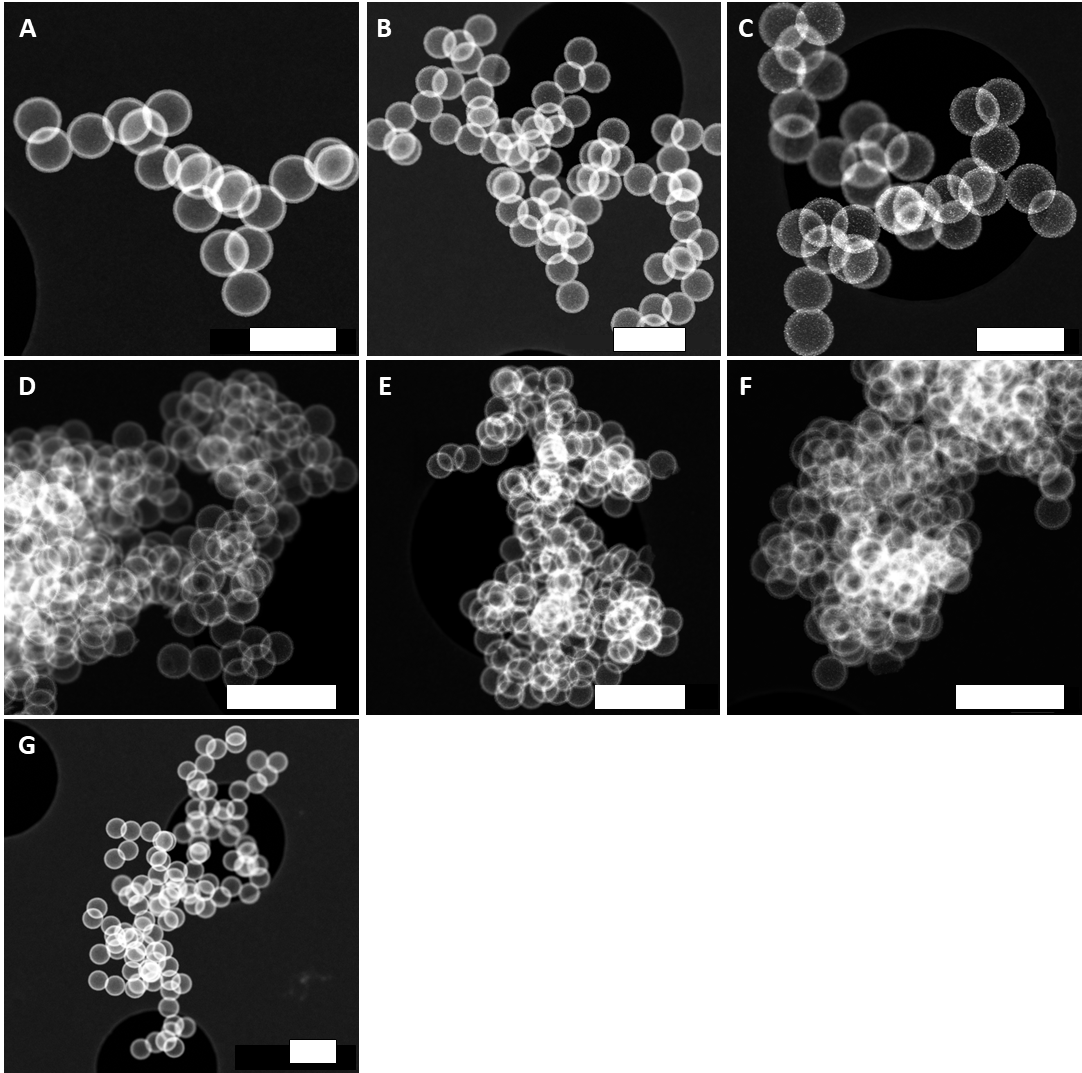


Figure S3 STEM HAADF images of CS-electrocatalysts and bare supports. A) $\mathbf{CS}_{\mathbf{Pt+}}$, B) $\mathbf{CS}_{\mathbf{Pt++}}$,C) $\mathbf{CS}_{\mathbf{Pt+++}}$, D) $\mathbf{CS}_{\mathbf{Pt +++}}^{\mathbf{TiO2+}}$, E) $\mathbf{CS}_{\mathbf{Pt +++}}^{\mathbf{TiO2++}}$, F) $\mathbf{CS}_{\mathbf{Pt +++}}^{\mathbf{TiO2+++}}$, G) unloaded CS. The scale bars correspond to 400 nm.


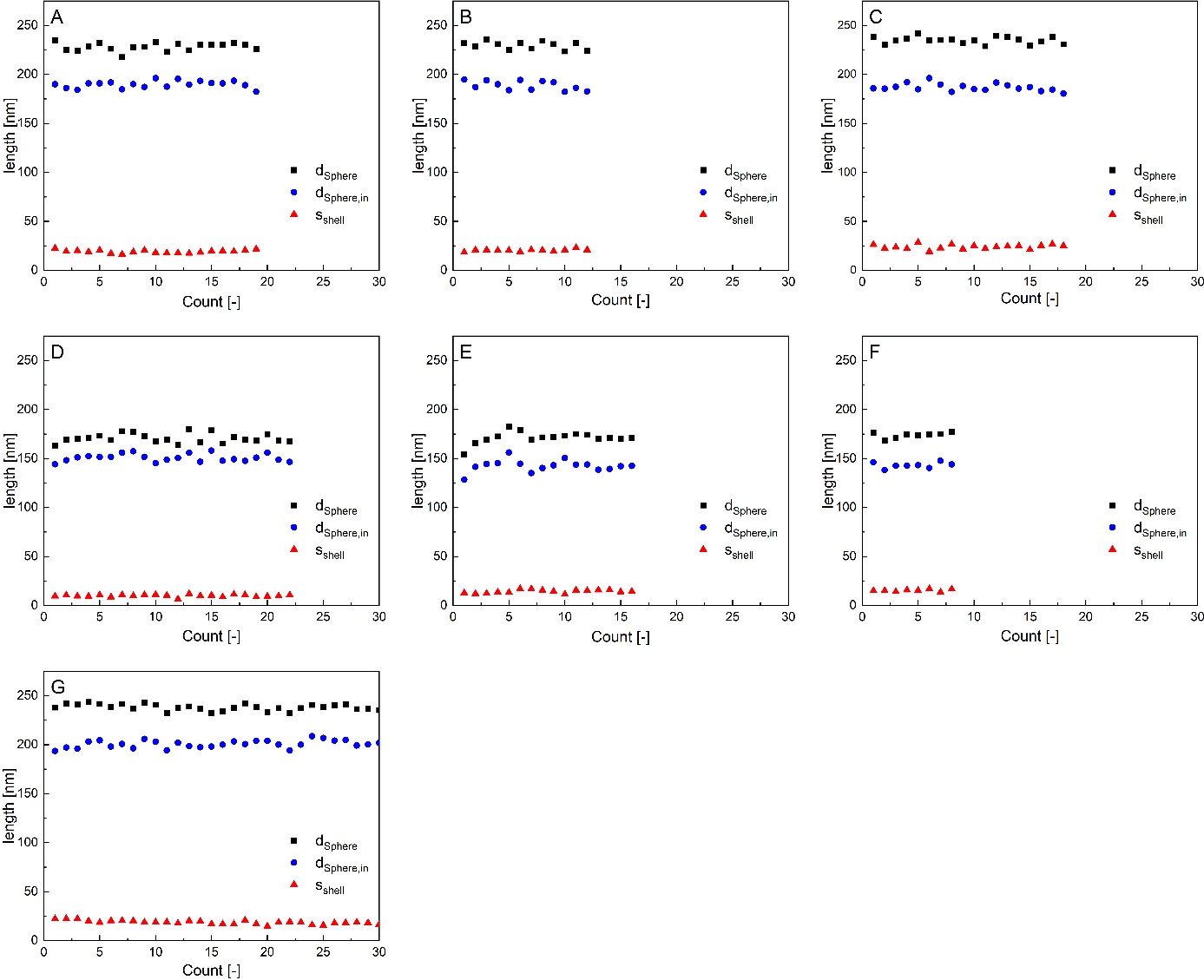


Figure S4 Outer and inner sphere diameters d_Sphere_ and d_Sphere,in_ and shell thickness s_Shell_ for A) $\mathbf{CS}_{\mathbf{Pt+}}$, B) $\mathbf{CS}_{\mathbf{Pt++}}$,C) $\mathbf{CS}_{\mathbf{Pt+++}}$, D) $\mathbf{CS}_{\mathbf{Pt +++}}^{\mathbf{TiO2+}}$, E) $\mathbf{CS}_{\mathbf{Pt +++}}^{\mathbf{TiO2++}}$, F) $\mathbf{CS}_{\mathbf{Pt +++}}^{\mathbf{TiO2+++}}$, G) non-loaded CS, derived from images shown in Figure S3. s_Shell_ was calculated from the TEM-derived outer and inner diameters as s_Shell_ = (d_Sphere_ – d_Sphere,in_)/2.

Table S1 Compositional, morphological and textural properties of supports and electrocatalysts.

| Sample | TiO_2_ | Pt_load_ | d_mean_^a)^ | d_Sphere_ | S_Shell_ | SSA | Pore volume | SSA | Pore volume |
| --- | --- | --- | --- | --- | --- | --- | --- | --- | --- |
|  | **[wt%]** | **[wt%]** | **[nm]** | **[nm]** | **[nm]** | **[m^2^ g^-1^]** | **[cm^3^ g^-1^]** | **[m^2^ g^-1^]** | **[cm^3^ g^-1^]** |
|  | **ICP-OES** | **ICP-OES** | **STEM** | **STEM** | **STEM** | **(N_2_ physisorption)** | **(N_2_ physisorption)** | **(CO_2_ physisorption)** | **(CO_2_ physisorption)** |
| CS | - | - | - | 238 ± 3 (n = 30) | 19 ± 2 (n = 30) | 799 ± 40 | 0.714 ± 0.04 | 974 ± 49 | 0.288 ± 0.01 |
| $\mathrm{CS}^{TiO2+}$ | 16.7 ± 0.4 | - | - | - | - | 673 ± 34 | 0.786 ± 0.04 | 1006 ± 50 | 0.353 ± 0.02 |
| $\mathrm{CS}^{TiO2++}$ | 22.5 ± 1.0 | - | - | - | - | 641 ± 32 | 0.777 ± 0.04 | 592 ± 30 | 0.194 ± 0.01 |
| $\mathrm{CS}^{TiO2+++}$ | 26.7 ± 0.3 | - | - | - | - | 514 ± 26 | 0.620 ± 0.03 | 481 ± 24 | 0.153 ± 0.01 |
| $\mathrm{CS}_{Pt+}$ | - | 1.8 ± 0.7 | 2.2 ± 1.3 | 228 ± 4 (n = 19) | 19 ± 2 (n = 19) | 855 ± 43 | 0.654 ± 0.03 | 944 ± 47 | 0.277 ± 0.01 |
| $\mathrm{CS}_{Pt++}$ | - | 4.01 ± 0.02 | 2.6 ± 1.3 | 230 ± 4 (n = 12) | 20 ± 1 (n = 12) | 682 ± 34 | 0.410 ± 0.02 | 916 ± 46 | 0.274 ± 0.01 |
| $\mathrm{CS}_{Pt+++}$ | - | 11.6 ± 0.01 | 3.5 ± 1.4 | 235 ± 3 (n = 18) | 24 ± 2 (n = 18) | 740 ± 37 | 0.370 ± 0.02 | 442 ± 22 | 0.160 ± 0.01 |
| $\mathrm{CS}_{Pt+++}$(AST) | - | - | 3.1 ± 1.3 | - | - | - | - | - | - |
| $\mathrm{CS}_{Pt +++}^{TiO2+}$ | 14.4 ± 0.2 | 10.3 ± 0.2 | 2.6 ± 1.2 | 171 ± 5 (n = 22) | 10 ± 1 (n = 22) | 502 ± 25 | 0.821 ± 0.04 | 502 ± 25 | 0.160 ± 0.01 |
| $\mathrm{CS}_{Pt +++}^{TiO2++}$ | 19.6 ± 1.1 | 11.0 ± 0.4 | 2.8 ± 1.3 | 171 ± 5 (n = 16) | 14 ± 2 (n = 16) | 495 ± 25 | 0.771 ± 0.04 | 415 ± 21 | 0.137 ± 0.01 |
| $\mathrm{CS}_{Pt +++}^{TiO2+++}$ | 23.9 ± 1.2 | 10.4 ± 0.4 | 2.8 ± 1.2 | 174 ± 3 (n = 8) | 15 ± 1 (n = 8) | 378 ± 19 | 0.739 ± 0.04 | 412 ± 21 | 0.132 ± 0.01 |
| $\mathrm{CS}_{Pt +++}^{TiO2+}$(AST) | - | - | 2.6 ± 1.3 | - | - | - | - | - | - |
| $\mathrm{CS}_{Pt +++}^{TiO2+}$(DUR) | - | - | 2.5 ± 1.3 | - | - | - | - | - | - |

a) From lognormal Pt particle size distributions. Uncertainty: Standard deviation of the lognormal distribution.


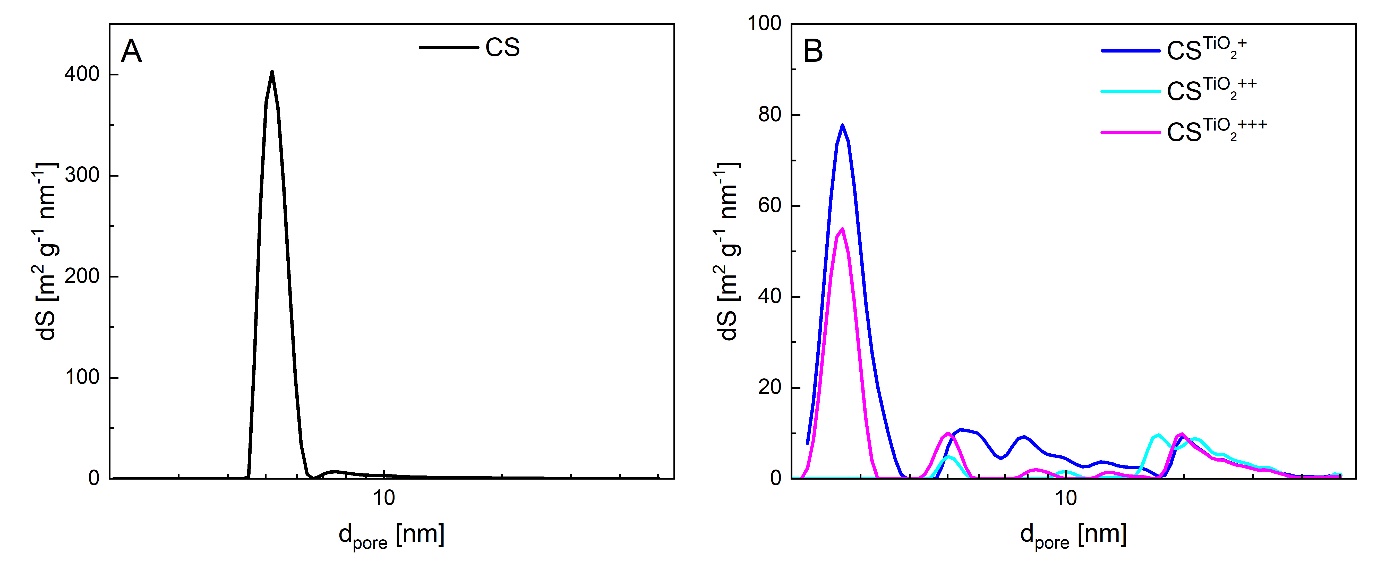


Figure S5 Areal pore size distributions, derived from nitrogen sorption isotherms (QSDFT, cyl., N_2_ on carbon) of the CS-support (A) and CS^TiO2^-supports (B).


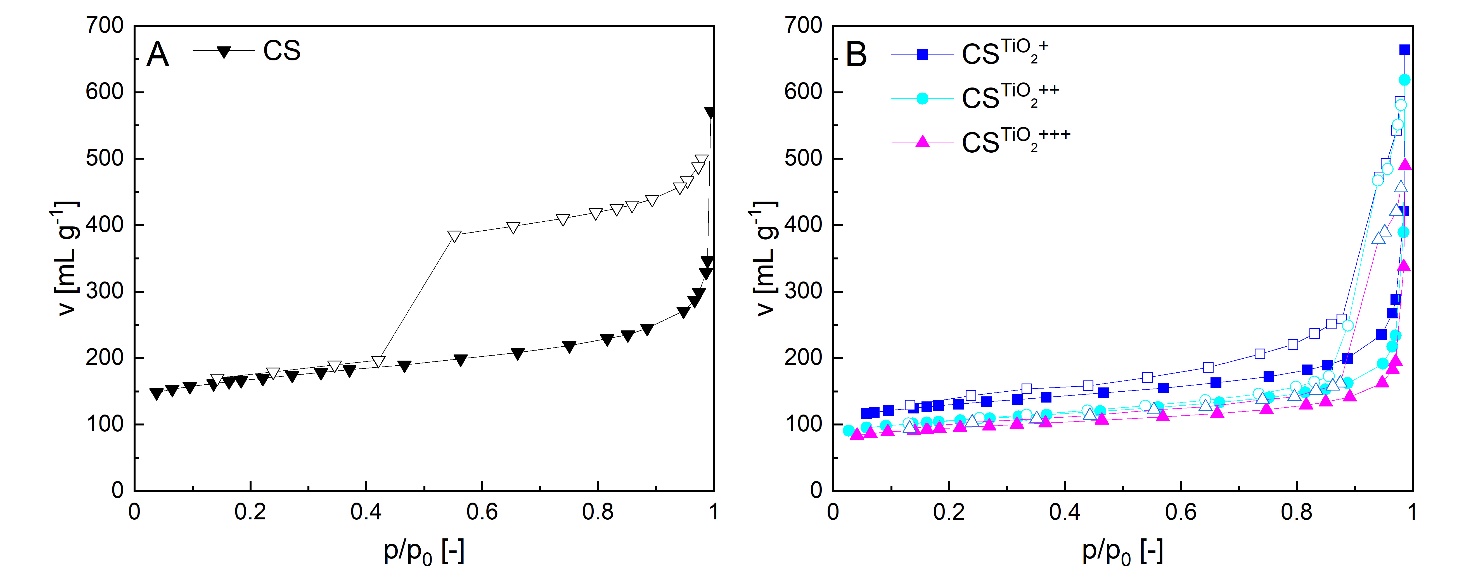


Figure S6 Nitrogen sorption isotherms (77 K) of the CS-support (A) and CS^TiO2^-supports (B). Adsorption cycle (solid symbols), desorption cycle (open symbols).

**
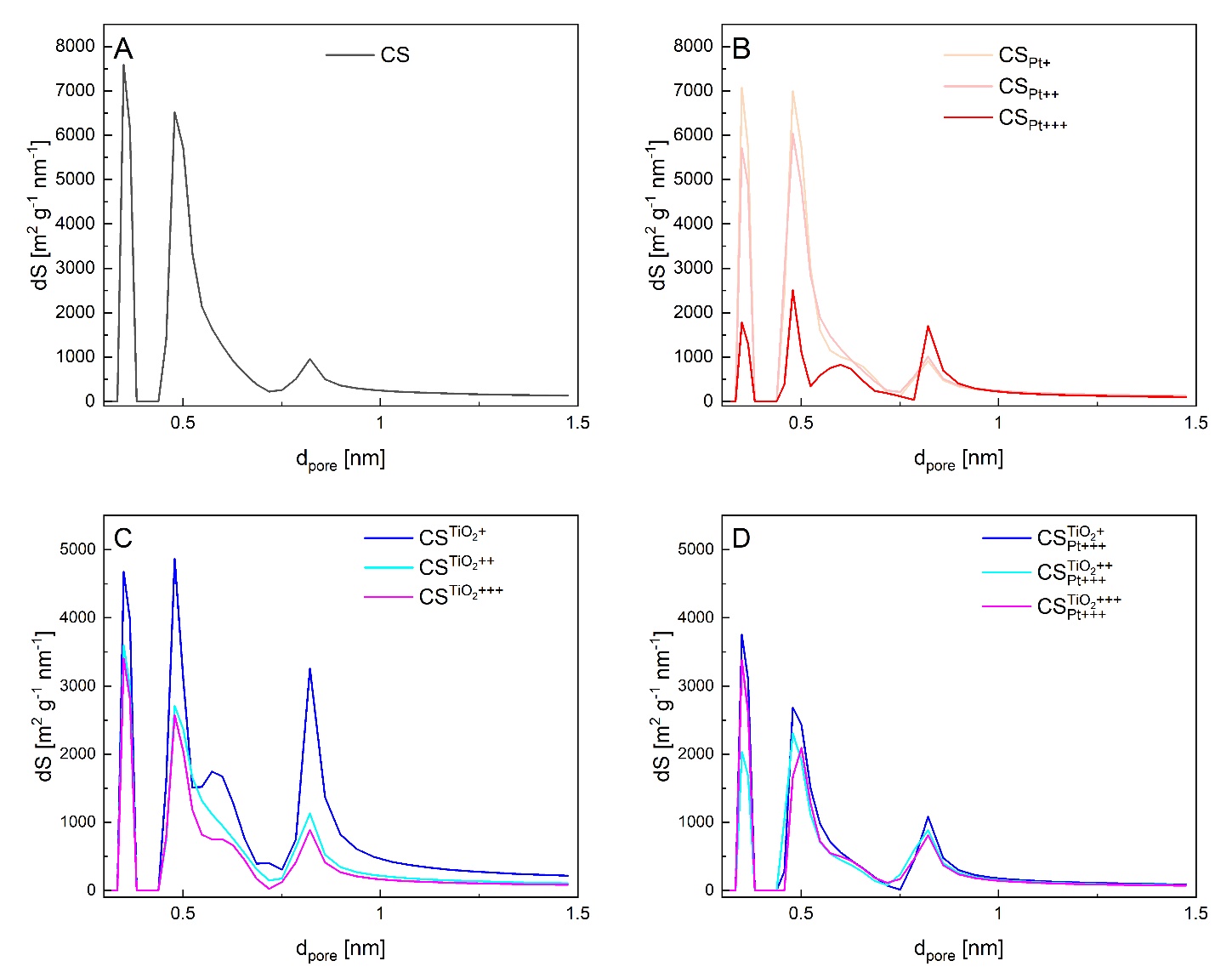
**

Figure S7 Micropore size distributions of supports and electrocatalysts derived from CO_2_ physisorption at 273.15 K.


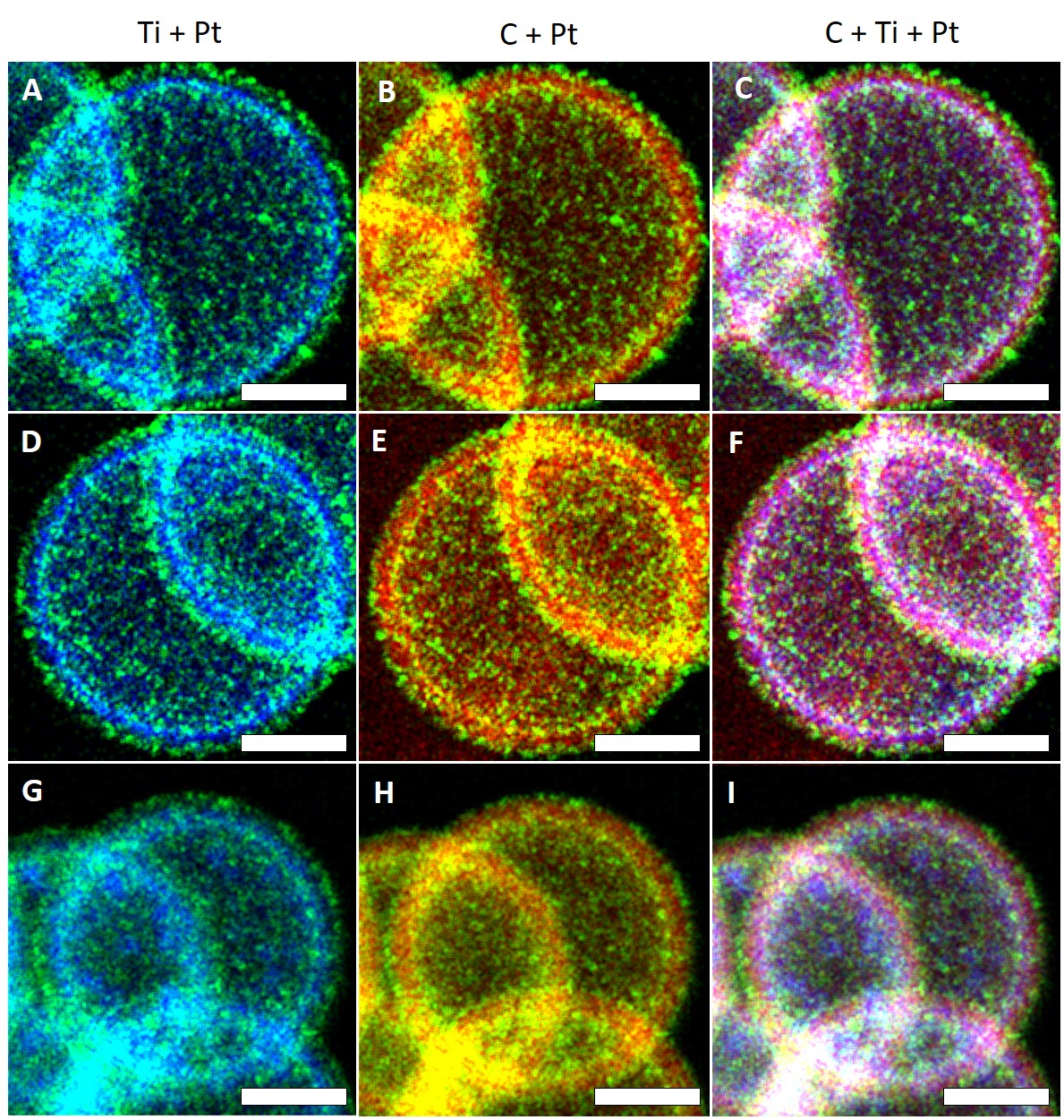


Figure S8 EDX overlapped elemental maps of different electrocatalysts. A – C) $\mathbf{CS}_{\mathbf{Pt +++}}^{\mathbf{TiO2+}}$, D – F) $\mathbf{CS}_{\mathbf{Pt +++}}^{\mathbf{TiO2++}}$, G – I) $\mathbf{CS}_{\mathbf{Pt +++}}^{\mathbf{TiO2+++}}$. Color code: red = C, blue = Ti, green = Pt. The scale bars correspond to 50 nm.

**
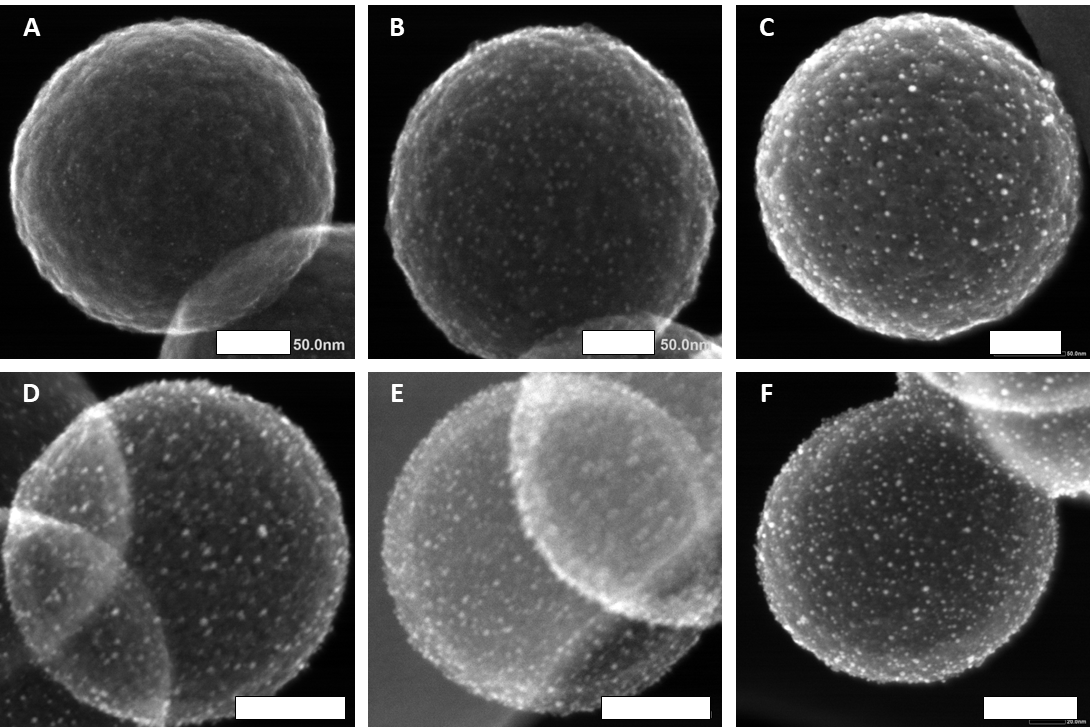
**

**Figure S9** STEM SEI images of A) $\mathrm{CS}_{Pt+}$, B) $\mathrm{CS}_{Pt++}$,C) $\mathrm{CS}_{Pt+++}$, D) $\mathrm{CS}_{Pt+++}^{TiO2+}$, E) $\mathrm{CS}_{Pt+++}^{TiO2++}$, F) $\mathrm{CS}_{Pt+++}^{TiO2+++}$. The scale bars correspond to 50 nm.

**
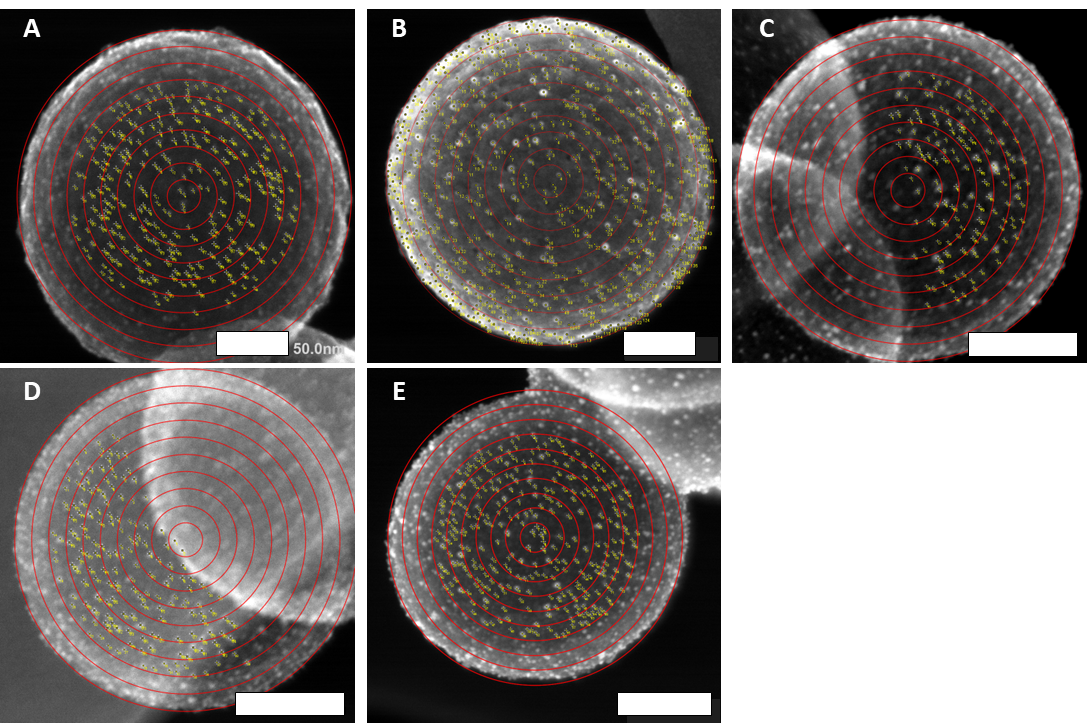
**

**Figure S10** STEM SEI images partitioned and with marked Pt particle counts of A) $\mathrm{CS}_{Pt++}$,B) $\mathrm{CS}_{Pt+++}$, C) $\mathrm{CS}_{Pt+++}^{TiO2+}$, D) $\mathrm{CS}_{Pt+++}^{TiO2++}$, E) $\mathrm{CS}_{Pt+++}^{TiO2+++}$. The scale bars correspond to 50 nm.

**
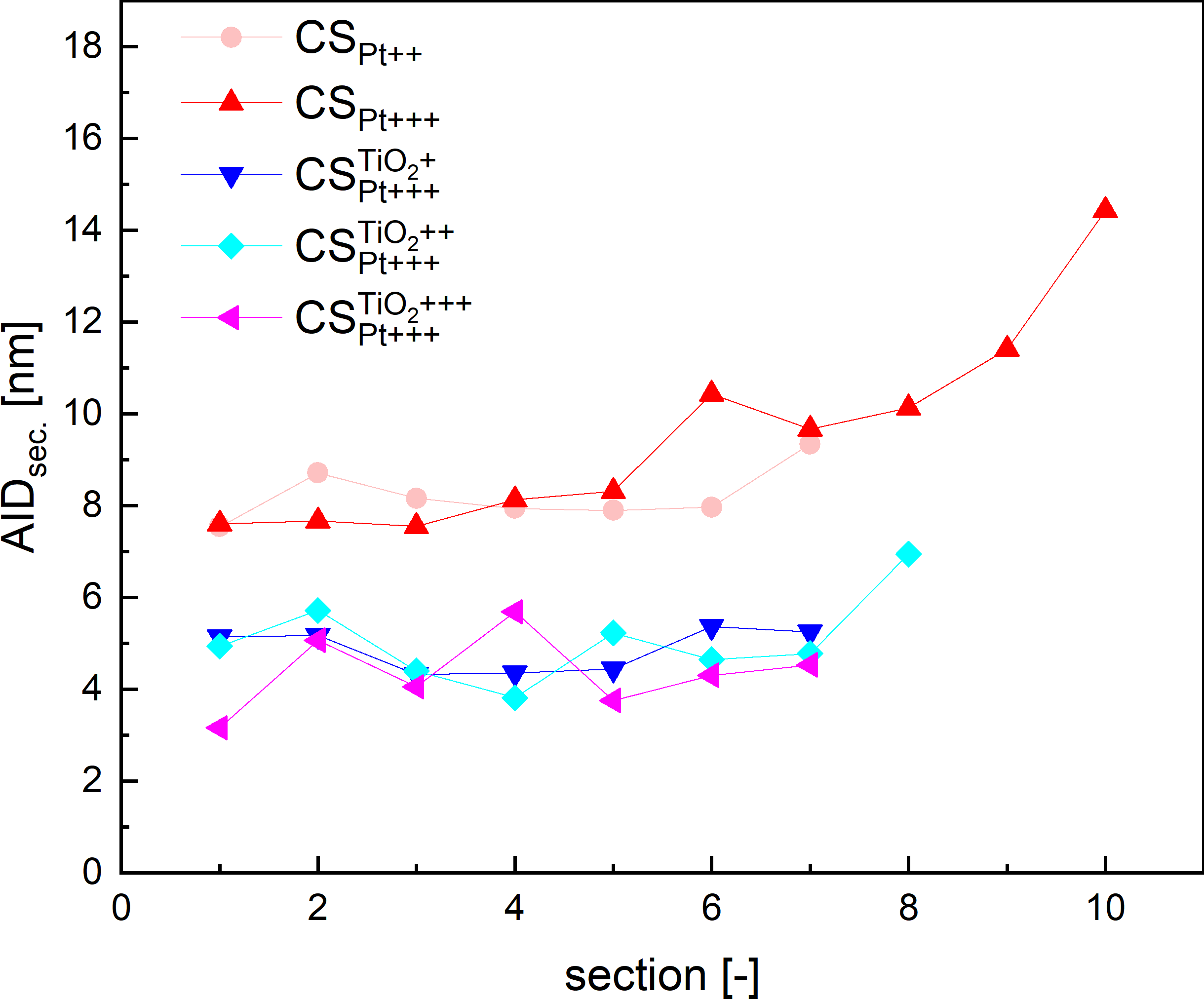
**

**Figure S11** Average interparticle distances AID_sec._ of different sections shown in **Figure S9**. The overall average interparticle distance AID of each electrocatalyst was calculated from the average of AID_sec._ values from sections 1 – 7 respectively.

**Evaluation of the average interparticle distance (AID)**

To obtain a measure for the average interparticle distance AID of supported Pt-NPs, their counts per area were evaluated with HAADF STEM images of single spherogels.

To ensure representative counts, only the sections 1-7 and areas without overlap from other spheres were evaluated to ensure good individual identifiability of Pt-NPs. For this purpose, the two-dimensional projection of the half-spheres were partitioned in ten, equally spaced concentric circles, starting from a manually placed circumference of the spherogel, also used as measurement for the outer sphere diameter D.

Pt-NPs were counted for each ring. To account for the actual curvature of the spherogel surface the area of each ring was calculated as annular spherical cap.

The partitioning of the evaluated surface areas in annular spherical caps allows for a quantification of the homogeneity of Pt-NP distribution on the observed spherogels.

Assumptions:

- Perfect spherical surface of spherogel
- All Pt-NPs show the same mean size *d_mean_* (taken from mean of log-normal distributions)
- All Pt-NPs present within the evaluated areas were counted
- Only Pt-NPs on the outer spherogel surface are seen on the pictures
- Perfect homogeneous distribution of Pt-NPs as densest circle packing (*η* = 0.907)

The area of the spherical cap *A_i_* with the radius *r_i_* of section i, with sphere diameter *R* = *r_10_* is given by:

| $A_{i}=2\cdot\pi\cdot R\cdot\left( R-\sqrt{R^{2}-r_{i}^{2}} \right)$ | **Eq. S1** |
| --- | --- |


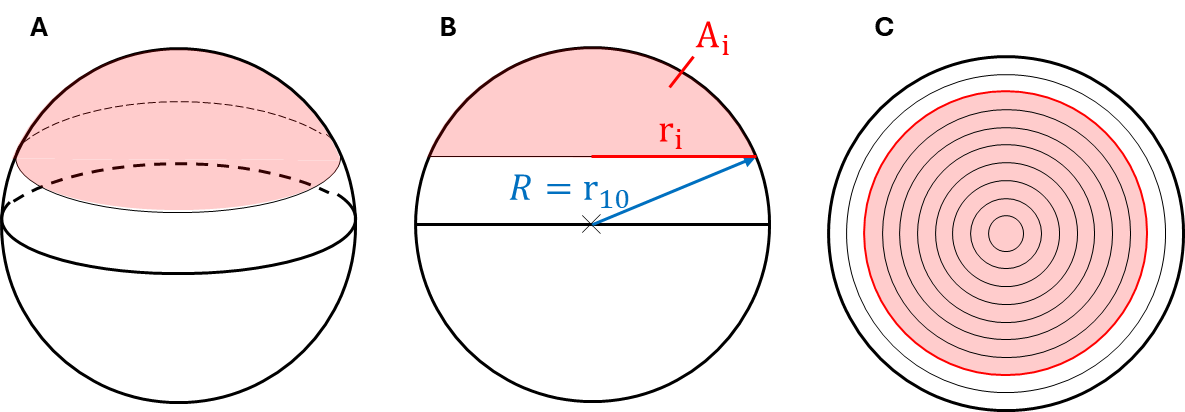


Figure S12 Sketch of spherical cap: Isometric (A), side projection (B), top projection with ten equal concentric partitions (C).

The area $A_{sec., i}$ of the i^th^ annular partition of a sphere are calculated by subtracting the area of the spherical cap i *A_i_* by the area of the next smaller one i-1 A_i-1_.

| $A_{sec., i}=A_{i}-A_{i-1} \left( with A_{0}=0m^{2} \right)$ | **Eq. S2** |
| --- | --- |

To derive the mean interparticle distance from the particle count per area the particles were assumed to be evenly distributed across the support’s surface via densest circle packing. The circle diameter *D* represents the distance in between the particle centres.


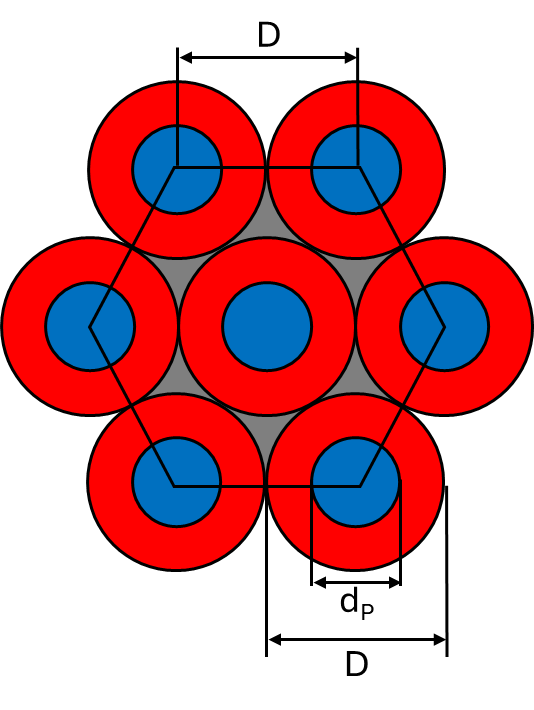


Figure S13 Sketch of spherical particles with diameter d_P_ distributed via densest circle packing at interparticle distance *D*.

Area of hexagon with side length *D*:

| $A_{H}=\frac{3\cdot\surd3}{2}\cdot D^{2}$ | **Eq. S3** |
| --- | --- |

Area of circle with diameter *D*:

| $A_{C}=\frac{\pi}{4}\cdot D^{2}$ | **Eq. S4** |
| --- | --- |

Area within hexagon covered with circles:

| $A_{HC}=3\cdot A_{C}=\frac{3\cdot\pi}{4}D^{2}$ | **Eq. S5** |
| --- | --- |

Packing density:

| $\eta=\frac{A_{HC}}{A_{H}}\approx0.907$ | **Eq. S6** |
| --- | --- |

Assuming a count of *n_C_* particles covering an area *A* via densest circle packing we obtain:

| $\eta\approx\frac{A_{C}\cdot n_{C}}{A} =\frac{\frac{\pi}{4}\cdot D^{2}\cdot n_{C}}{A}$ | **Eq. S7** |
| --- | --- |

With the resulting shortest distance between circle centres *D*:

| $D=\sqrt{\frac{4\cdot\eta\cdot A}{\pi\cdot n_{C}}}$ | **Eq. S8** |
| --- | --- |

Subtracting the mean particle diameter *d_mean_*, the mean interparticle distance *AID_mean_* can be calculated:

| ${AID}_{mean}=D-d_{P}=\sqrt{\frac{4\cdot\eta\cdot A}{\pi\cdot n_{C}}}-d_{mean}$ | **Eq. S9** |
| --- | --- |

Applying this formula to the area *A_sec.,i_* of a concentric annular spherical cap with the particle count *n_i_* the average interparticle distance per section (*AID_sec._*) is obtained:

| ${AID}_{sec.}=\sqrt{\frac{4\cdot\eta\cdot A_{sec., i}}{\pi\cdot n_{i}}}-d_{mean}$ | **Eq. S10** |
| --- | --- |

Table S2 Average interparticle distances derived from micrographs (AID*)* and from calculation via Eq.3 (AID_calc._*).* Assumption of specific surface area for the calculation: SSA (N_2_) (see Table S1).

| Sample | AID_calc._ | AID |
| --- | --- | --- |
|  | **[nm]** | **[nm]** |
| $\mathrm{CS}_{Pt+}$ | 139 | - |
| $\mathrm{CS}_{Pt++}$ | 106 | 8.2 ± 0.4 |
| $\mathrm{CS}_{Pt+++}$ | 97 | 8.5 ± 1.0 |
| $\mathrm{CS}_{Pt+++}^{TiO2+}$ | 55 | 4.9 ± 0.4 |
| $\mathrm{CS}_{Pt+++}^{TiO2++}$ | 58 | 4.8 ± 0.6 |
| $\mathrm{CS}_{Pt +++}^{TiO2+++}$ | 53 | 4.4 ± 0.8 |

**Alternative Pt loading via wet impregnation**

Wet impregnation was employed solely as a qualitative reference method. Toluene was selected as the solvent, as the Pt precursor Pt(cod)me₂ is known to be readily soluble in aromatic hydrocarbons. To enable a direct comparison with supercritical deposition (SCD), the same support-to-precursor mass ratio was used as in the SCD experiments for production of $\mathrm{CS}_{Pt+++}$ / $\mathrm{CS}_{Pt+++}^{TiO2}$samples. Therefore, 39.99 mg of CS^TiO2+++^ were placed in a 25 mL volumetric flask, and 12.86 mL of toluene were added. The spherogels were gently dispersed by manually swirling the flask until a homogeneous suspension was obtained. Subsequently, 17.14 mg of the Pt precursor were added to the suspension. The mixture was stirred at room temperature for 48 h. After impregnation, the solvent was removed by heating the suspension to 80 °C until complete evaporation of toluene. The resulting precursor-loaded sample was finally subjected to thermal conversion under identical conditions as applied for the SCD-prepared samples.The wet impregnated sample $\mathrm{CS}_{Pt+++}^{TiO2+++}-WI$contained a similar Pt loading (10.7 ± 0.6 wt%) as compared to the average of $\mathrm{CS}_{Pt+++}$ / $\mathrm{CS}_{Pt+++}^{TiO2}$ samples. This showed that the amount of adsorbed Pt was independent of the loading method and depended solely on the support to Pt precursor ratio.


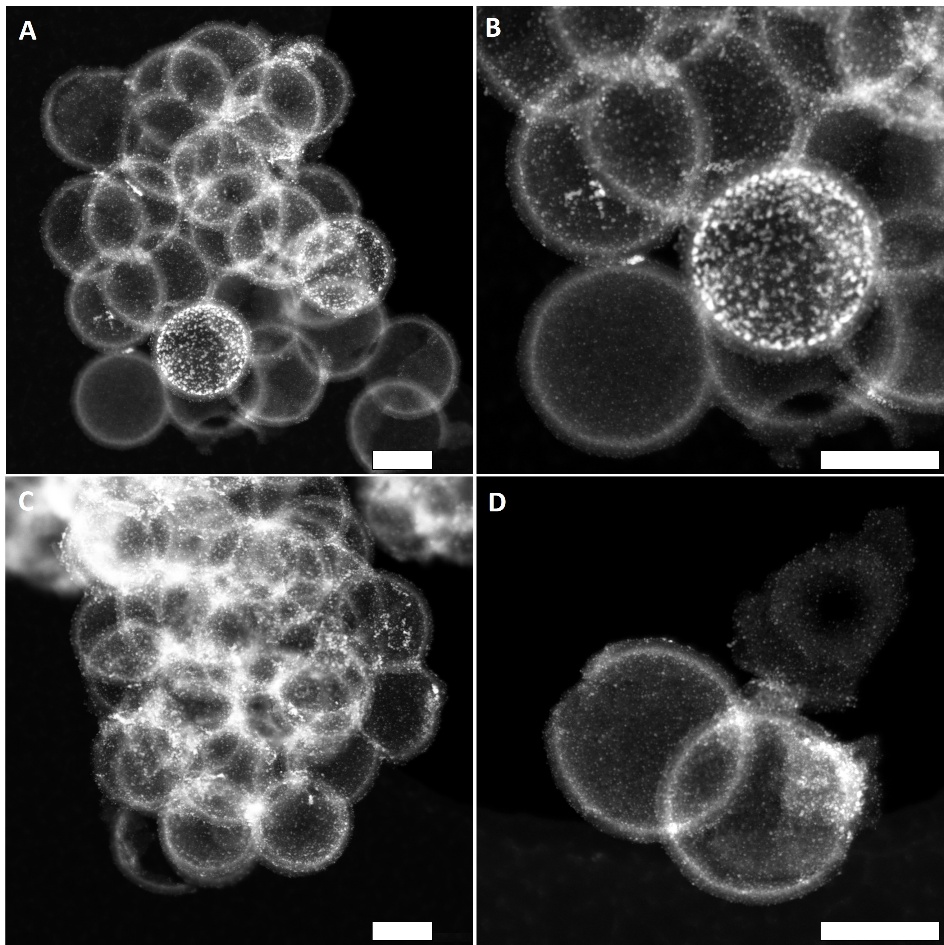


Figure S14 STEM HAADF images of $\mathbf{CS}_{\mathbf{Pt+++}}^{\mathbf{TiO2+++}}\mathbf{-WI}$. The scale bars correspond to 100 nm.

STEM-HAADF imaging revealed that PT-NPs were successfully formed, however they showed varying sizes on individual spheres (**Figure S14 A, B**) and were unevenly distributed (**Figure S14 C, D**). Nitrogen physisorption analysis confirmed that the sample contained mesopores (**Figure S15, A**). The micropore size distribution (**Figure S15, B**) was similar to those of the non-loaded CS^TiO2^ supports and SCD loaded samples (**Figure S7**) and the according micropore SSA (430 ± 22 m^2^g^-1^) comparable to the SCD-prepared samples, showing that the choice of the Pt loading method did not affect the meso- and microporosity of the support.

**
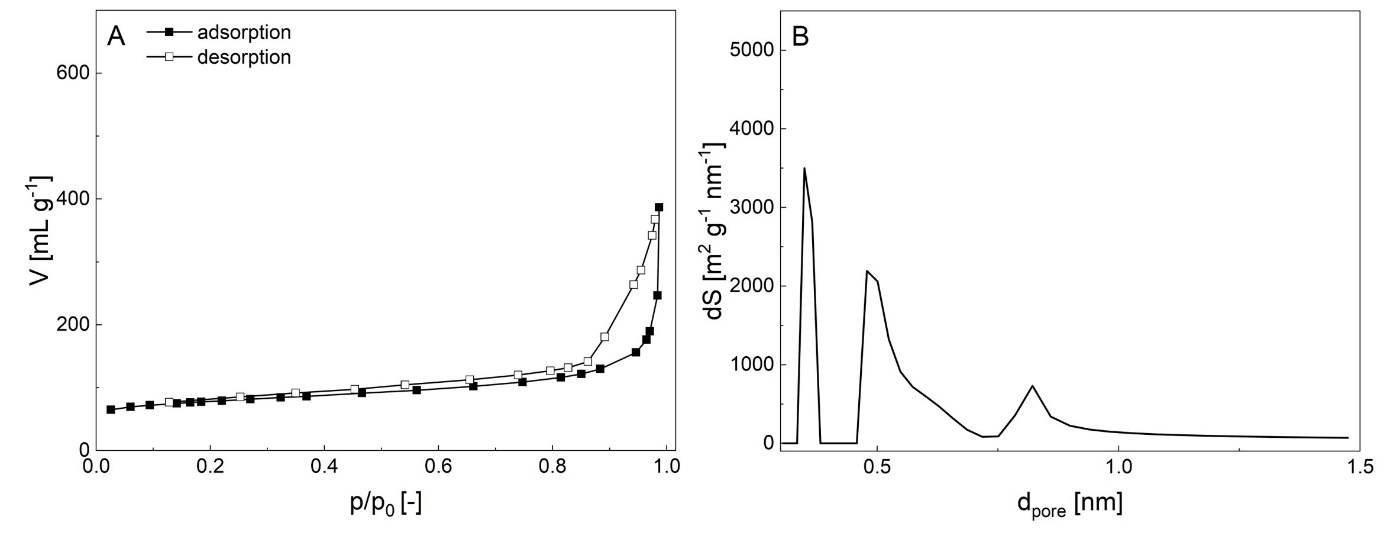
**

**Figure S15** B) (A) Nitrogen sorption isotherm (77 K) and (B) micropore size distribution (derived from CO_2_ physisorption at 273.15 K) of $\mathrm{CS}_{Pt+++}^{TiO2+++}-WI$.


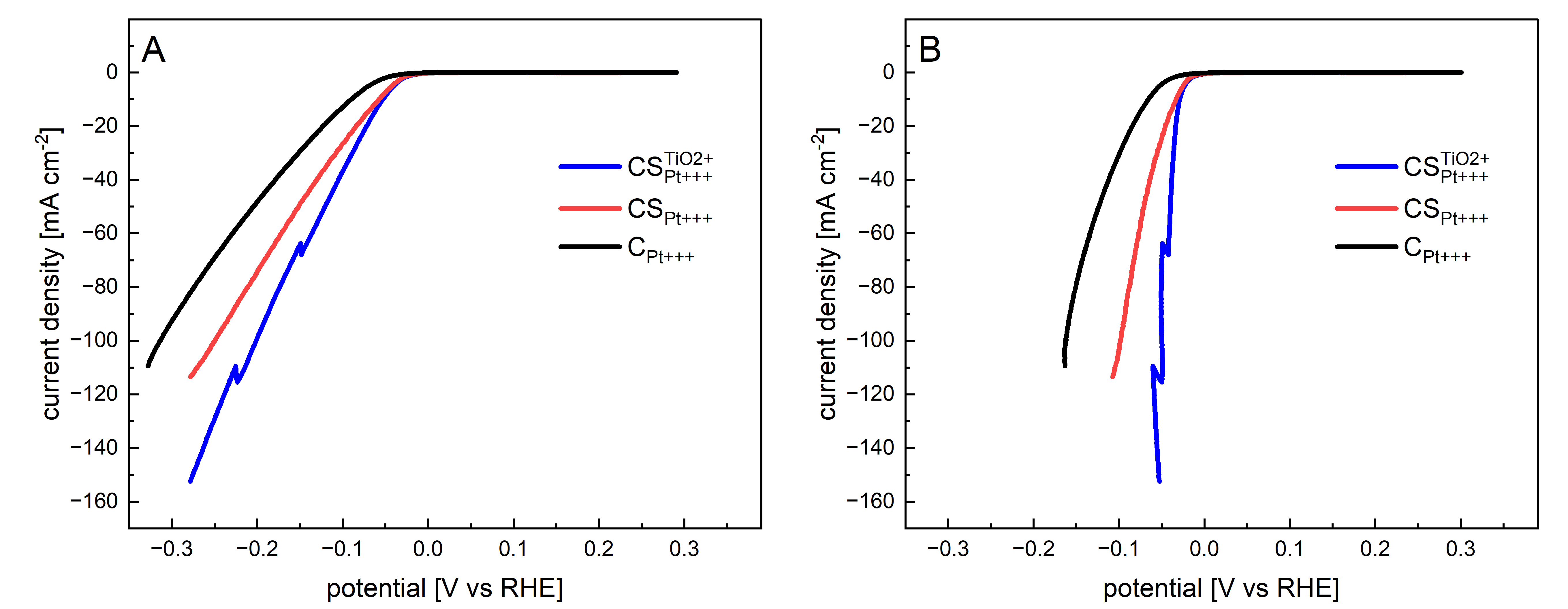


**Figure S16** LSV curves of SCD-prepared electrocatalysts in N_2_-saturated 0.5 M H_2_SO_4_. (A) Original polarization curves and (B) corresponding iR-corrected curves using R_s_ = 7.2 Ω.


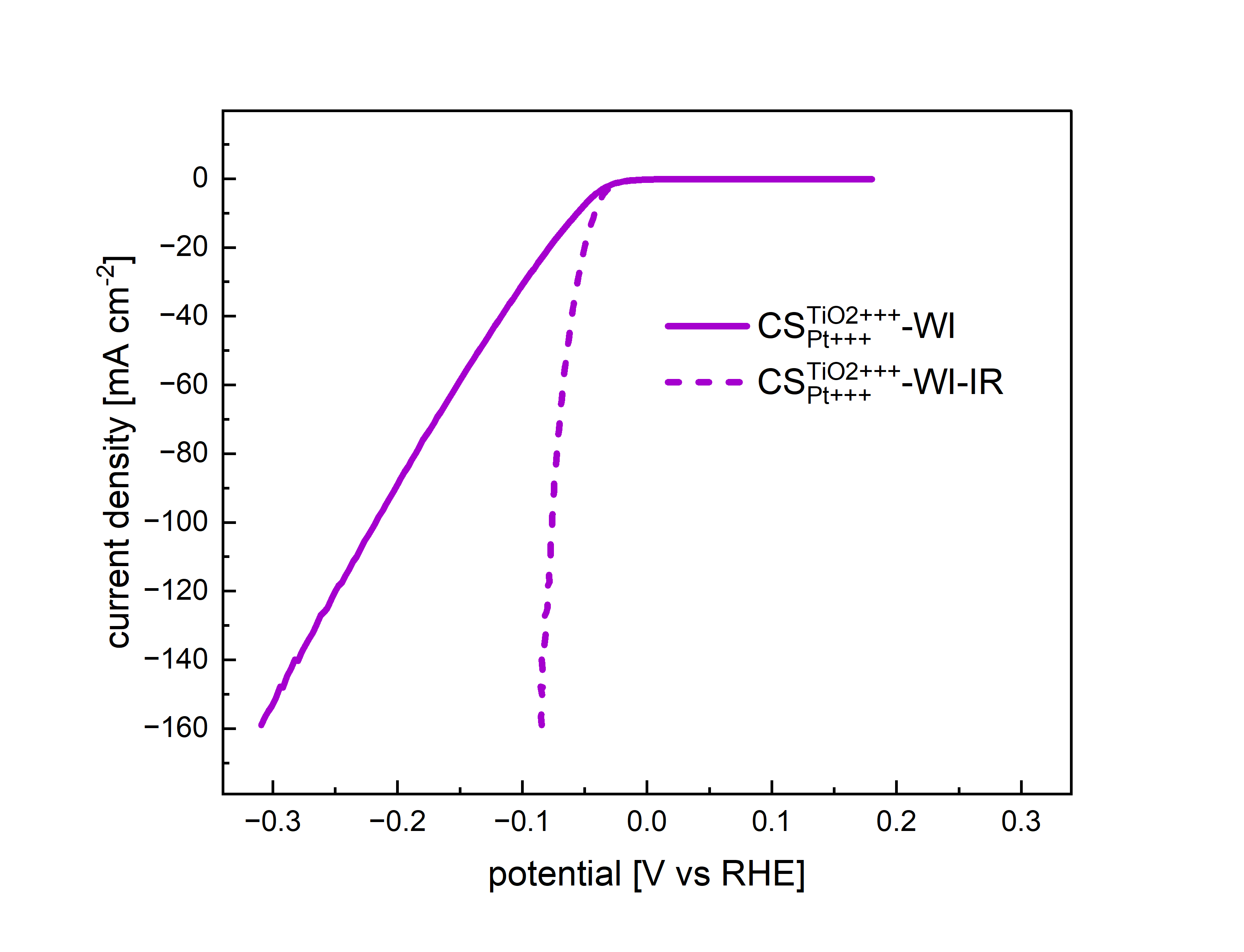


**Figure S17**  LSV curves of the $CS_{Pt+++}^{TiO2+++}-WI$ electrocatalyst before and after iR correction (WI-IR).

Table S3 Electrochemical properties of different electrocatalysts.

| Sample | Tafel slope | Overpotential  [mV] | | | Apparent mass activity  @ 40 mV |
| --- | --- | --- | --- | --- | --- |
|  | **[mV dec^-1^]** | **@ 10 mA cm^-2^** | **@ 20 mA cm^-2^** | **@ 40 mA cm^-2^** | **[A mg^-1^_Pt_]** |
| $\mathrm{CS}_{Pt+}$ | 37 ± 0 | 69.8 ± 0.4 | 101.2 ± 1.8 | 150.3 ± 3.8 | 1.77 ± 0.01 |
| $\mathrm{CS}_{Pt++}$ | 32 ± 3 | 56.7 ± 2.0 | 80.8 ± 3.0 | 123.4 ± 4.1 | 1.11 ± 0.08 |
| $\mathrm{CS}_{Pt+++}$ | 34 ± 0.4 | 52.8 ± 0.7 | 72.9 ± 0.1 | 106.6 ± 0.8 | 0.42 ± 0.01 |
| $\mathrm{CS}_{Pt +++}^{TiO2+}$ | 27 ± 1 | 46.0 ± 0.5 | 61.9 ± 0.8 | 90.6 ± 1.1 | 0.66 ± 0.04 |
| $\mathrm{CS}_{Pt+++}^{TiO2++}$ | 30 ± 1 | 48.8 ± 0.9 | 65.4 ± 0.6 | 94.9 ± 0.8 | 0.53 ± 0.06 |
| $\mathrm{CS}_{Pt +++}^{TiO2+++}$ | 30 ± 2 | 47.0 ± 0.8 | 64.4 ± 1.2 | 95.6 ± 2.2 | 0.64 ± 0.03 |
| $\mathrm{CS}_{Pt+++}^{TiO2+++}-WI$ | 30 ± 0.5 | 56.1 ± 0.6 | 78.4 ± 0.6 | 116.9 ± 0.7 | 0.42 ± 0.02 |
| $C_{Pt+++}$ | 41 ± 0.9 | 76.9 ± 3.2 | 110.3 ± 3.2 | 129.9 ± 32.3 | 0.24 ± 0.01 |


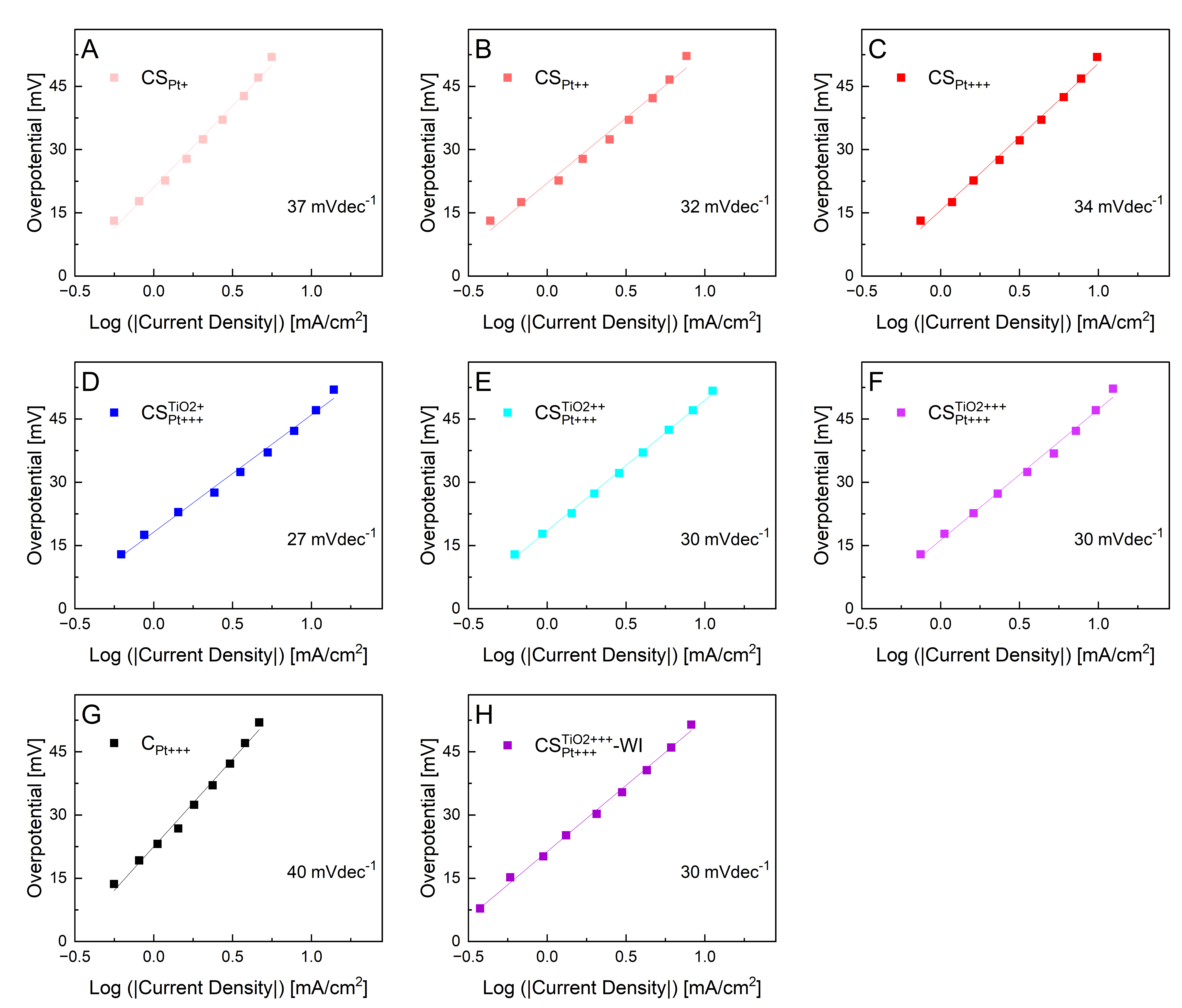


Figure S18 Tafel plots and according slopes of all electrocatalysts.

**Cyclic voltammetry (CV)**

CV measurements were performed at a scan rate of 50 mV s^-1^ over a potential range of 0.05 to 1.1 V vs RHE. The electrolyte (0.5 M H_2_SO_4_) was purged with N_2_ for 30 minutes before conducting the CV measurements. The electrochemical surface area (A_ESA_, in m^2^ g Pt^-1^) can generally be determined using equation S11, based on the charge associated with hydrogen adsorption in the negative current region of the cyclic voltammetry (CV) data. The parameter Q (A · m^-2^ · mV) corresponds to the integrated area under the hydrogen adsorption peak, while A (m^2^) represents the geometric area of the electrode. S (V·s^-1^) denotes the scan rate, and q is the charge required for the formation of a hydrogen monolayer taken as 2.1 C·m^-2^.^[S1]^ The A_ESA_ is thus calculated by normalizing the adsorption charge with respect to the scan rate and the Pt mass.

| $A_{\mathrm{ESA}}=\frac{Q \cdot A}{S \cdot m_{\mathrm{Pt}}\cdot q}$ | **Eq. S11** |
| --- | --- |

Since the catalyst ink deposition resulted in a total catalyst loading of approximately L_cat_≈ 100 µg cm^-2^, the Pt mass loading on the electrode m_Pt_ was calculated as:

$m_{\mathrm{Pt}}\approx L_{\mathrm{cat}} \frac{\mathrm{Pt}_{\mathrm{load}}}{100}$ **Eq. S12**

which simplifies to m_Pt_ [µg cm^-2^] ≈ Pt_load_ [wt%] under the present experimental conditions. Accurate estimation of A_ESA_ becomes challenging for electrocatalysts with a limited number of adsorption sites, either due to low Pt loading or the presence of large Pt nanoparticles. This results in a diminished adsorption peak area and difficulty in defining the adsorption region. As illustrated in **Figure S15, S16**, the hydrogen adsorption region is clearly defined for
$\mathrm{CS}_{Pt +++}^{TiO2+}$compared to $CS_{Pt+++}$ and $C_{Pt+++}$. For reference, CVs of the bare CS and $CS^{TiO2+}$supports were additionally recorded (**Figure S15**): as expected, no hydrogen adsorption features were observed, confirming that was originated exclusively from Pt-containing interfaces. The bare CS support shows a slightly higher apparent capacitive background current than $CS^{TiO2+}$, which is attributed to its higher SSA, consistent with N_2_ and CO_2_ physisorption results, and to the lower electronic conductivity of TiO_2_ compared to carbon. While a quantitative comparison of samples was therefore not possible, the discernible hydrogen adsorption features observed for $\mathrm{CS}_{Pt +++}^{TiO2+}$indicate an increased apparent availability of hydrogen adsorption sites compared to $CS_{Pt+++}$ and $C_{Pt+++}$. As discussed in the next section, this effect cannot be attributed solely to differences in geometric surface area and likely reflects additional Pt–TiO₂ interfacial and electronic contributions, which may contribute to the enhanced HER performance of $\mathrm{CS}_{Pt +++}^{TiO2+}$.


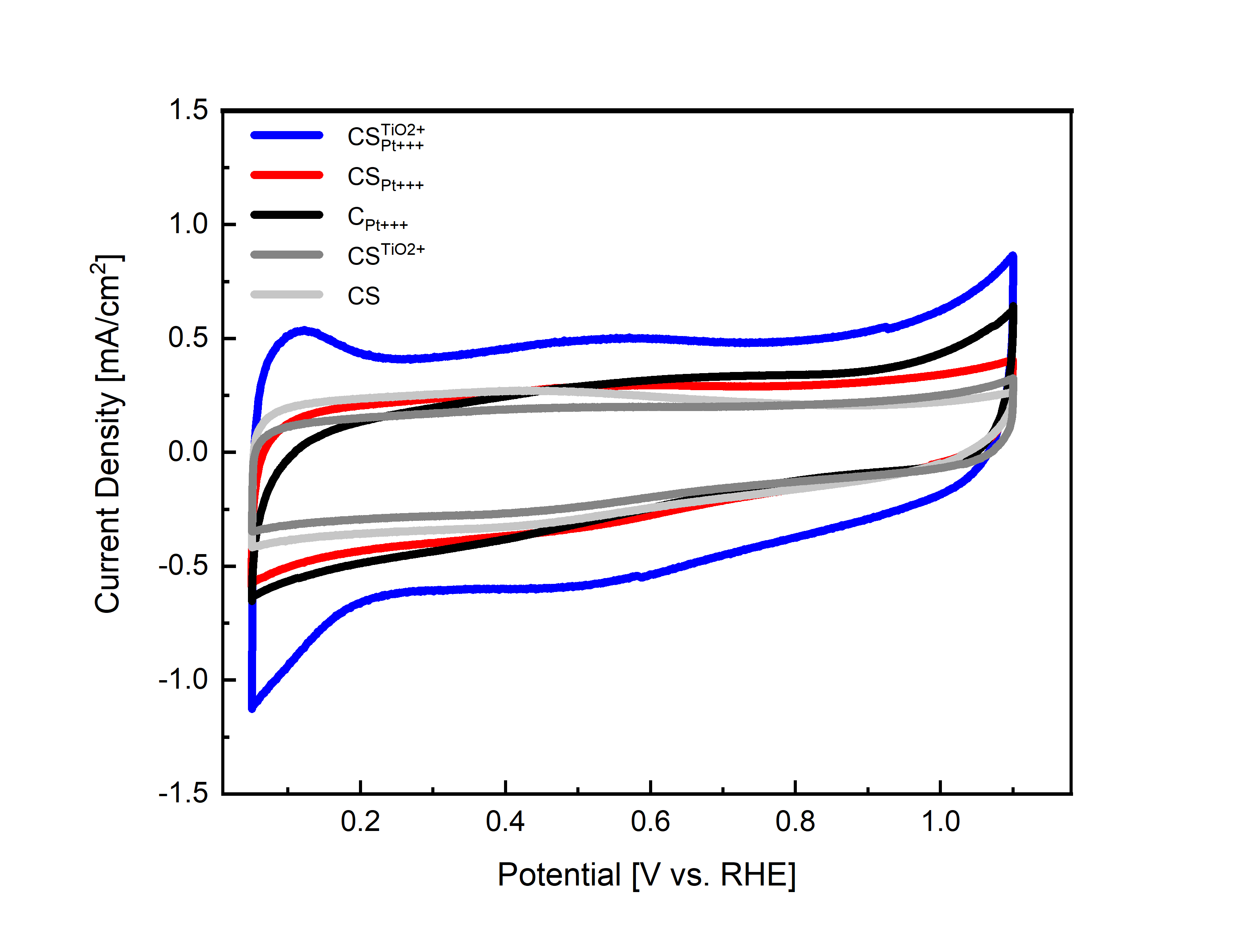


Figure S19 Cyclic voltammetry analysis of selected supports and electrocatalysts.


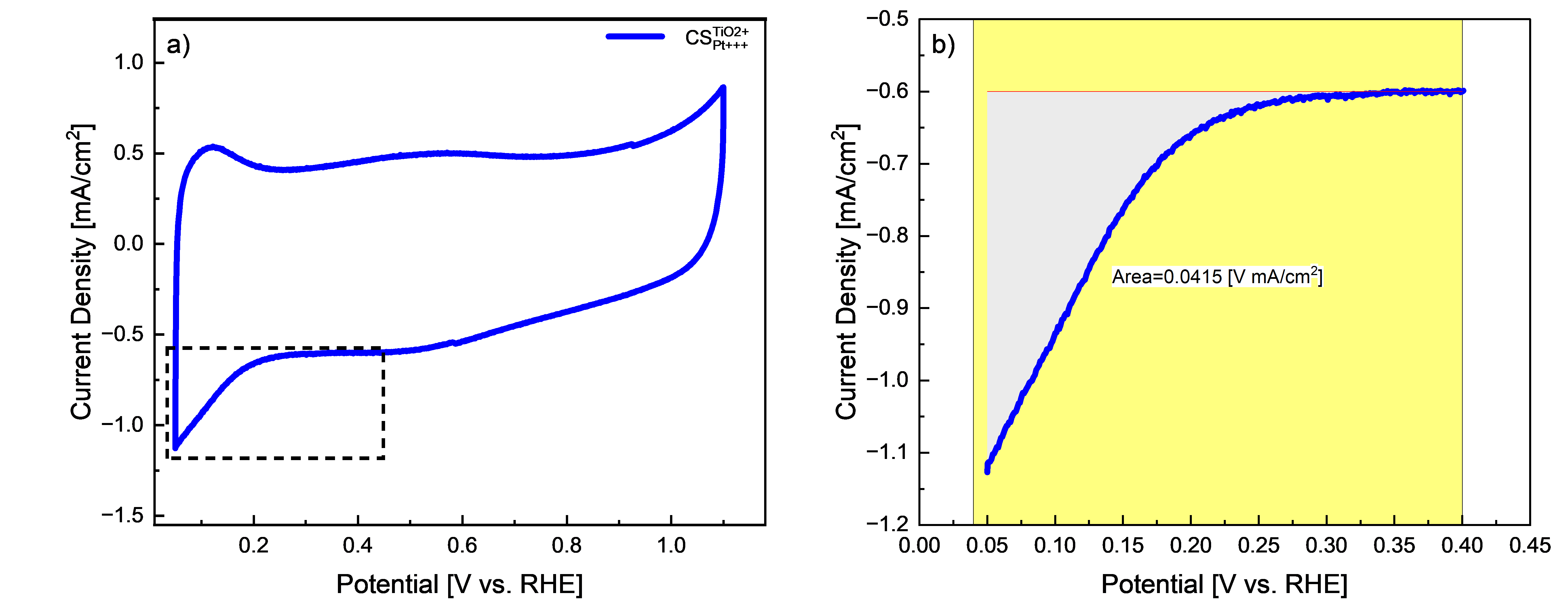


**Figure S20** (a) Cyclic voltammetry analysis of $CS_{Pt+++}^{TiO2+++}$ (b) the hydrogen adsorption area with respect to the base line at -0.6 mA/cm^2^.

**Geometric estimation of the accessible Pt surface area**

Since the quantitative determination A_ESA_ was not possible for samples via CV due to the absence of well-defined hydrogen adsorption features, we additionally provide a geometry-based estimation of the accessible Pt surface area derived from STEM particle size distributions, as described in the following. Such geometry-based surface area estimates are commonly used as complementary reference values when electrochemical surface area determination is ambiguous for supported Pt-NPs, and should be interpreted as theoretical upper bounds rather than the absolute A_ESA_*.*^[S2, S3]^ In our case, this approach serves as an independent, quantitative estimate of the accessible Pt surface area for those materials for which statistically meaningful particle size distributions could be obtained from STEM–HAADF imaging, namely the $CS_{\mathrm{Pt}}$ and $CS_{\mathrm{Pt}}^{TiO2}$electrocatalysts. The applicability of this geometric approach is supported by the well-defined nanoparticle morphology and the homogeneous particle size distributions observed within the spherogel-based electrocatalysts, which enable a robust statistical evaluation of particle sizes and justify the use of a geometry-based surface-area estimate. The commercial $C_{Pt+++}$ reference material was excluded from this analysis, as its apparent particle size distribution was non-representative due to pronounced local inhomogeneities. Assuming spherical Pt-NPs with diameter d and the bulk density of platinum ρ_Pt_ = 21.45 g cm^-3^, the specific Pt surface area (SSA_Pt_, surface area per Pt mass) is given by:

$\mathrm{SSA}_{\mathrm{Pt}}=\frac{6}{\rho_{\mathrm{Pt}}\cdot d}$ **Eq. S13**

Using d in nm and converting the surface area from cm^2^ g^-1^ to m^2^ g^-1^ yields the approximation:

$\mathrm{SSA}_{\mathrm{Pt}}\approx\frac{280}{d_{\mathrm{nm}}}[m^{2} g^{-1}]\text{ }$ **Eq. S14**

The numerical prefactor (280) results from inserting the bulk density of Pt and the corresponding unit conversions. As the experimentally observed particle size distributions are not strictly monodisperse, the surface-area-relevant Sauter mean diameter $d_{3,2}=\sum d_{i}^{3}/\sum d_{i}^{2}$ was calculated directly from the STEM-derived individual particle diameters and used as d for the geometric surface-area estimation (**Table S4**).

Table S4 Geometric parameters derived from STEM Pt-NP size distributions.

| Sample | d_3,2_ | SSA_Pt_ | ECSA_geom_ |
| --- | --- | --- | --- |
|  | **[nm]** | **[m^2^ g^-1^]** | **[cm^2^_Pt_ cm^-2^_geom_]** |
|  |  |  |  |
| $\mathrm{CS}_{Pt+}$ | 2.6 | 109 | 2.0 |
| $\mathrm{CS}_{Pt++}$ | 2.9 | 96 | 3.9 |
| $\mathrm{CS}_{Pt+++}$ | 4.5 | 63 | 7.3 |
| $\mathrm{CS}_{Pt +++}^{TiO2+}$ | 2.8 | 99 | 10.2 |
| $\mathrm{CS}_{Pt +++}^{TiO2++}$ | 3.2 | 88 | 9.7 |
| $\mathrm{CS}_{Pt +++}^{TiO2+++}$ | 3.1 | 91 | 9.4 |

To relate these values to the electrode configuration, the geometric electrochemically accessible surface area per geometric electrode area ECSA_geom_, was estimated using the Pt mass fractions (Pt_load_, wt%) determined by ICP-OES (**Table S1**) and the total electrocatalyst mass loading on the glassy-carbon disk. Here, cm^2^_geom_ refers to the geometric surface area of the glassy-carbon electrode (0.196 cm² for the 5 mm disk used in this work). ECSA_geom_ was then calculated according to

$\mathrm{ECS}A_{\mathrm{geom}}=\mathrm{SSA}_{\mathrm{Pt}}\cdot m_{\mathrm{Pt}}\cdot{10}^{4}$ **Eq. S15**

resulting in geometric ECSAs in the range of approximately 2 - 10 cm^2^_Pt_ cm^-2^_geom_ (**Table S4**). These values represent theoretical upper bounds of the electrochemically accessible Pt surface area, as they assume ideal spherical particles and full surface accessibility. When comparing electrocatalysts with similar Pt mass fractions ($CS_{Pt+++}$ and $\mathrm{CS}_{Pt +++}^{TiO2+}$) the geometric ECSA differs by up to ~40%. While such differences may contribute to the observed variations in H-UPD features, they are insufficient to account for the pronounced qualitative changes in the H-UPD profiles, indicating that Pt–support interfacial and electronic effects play a significant additional role. Taken together, the CV analysis reveals qualitative changes in hydrogen adsorption behavior, while the geometry-based ECSA estimation demonstrates that these changes cannot be rationalized solely by differences in Pt surface area.


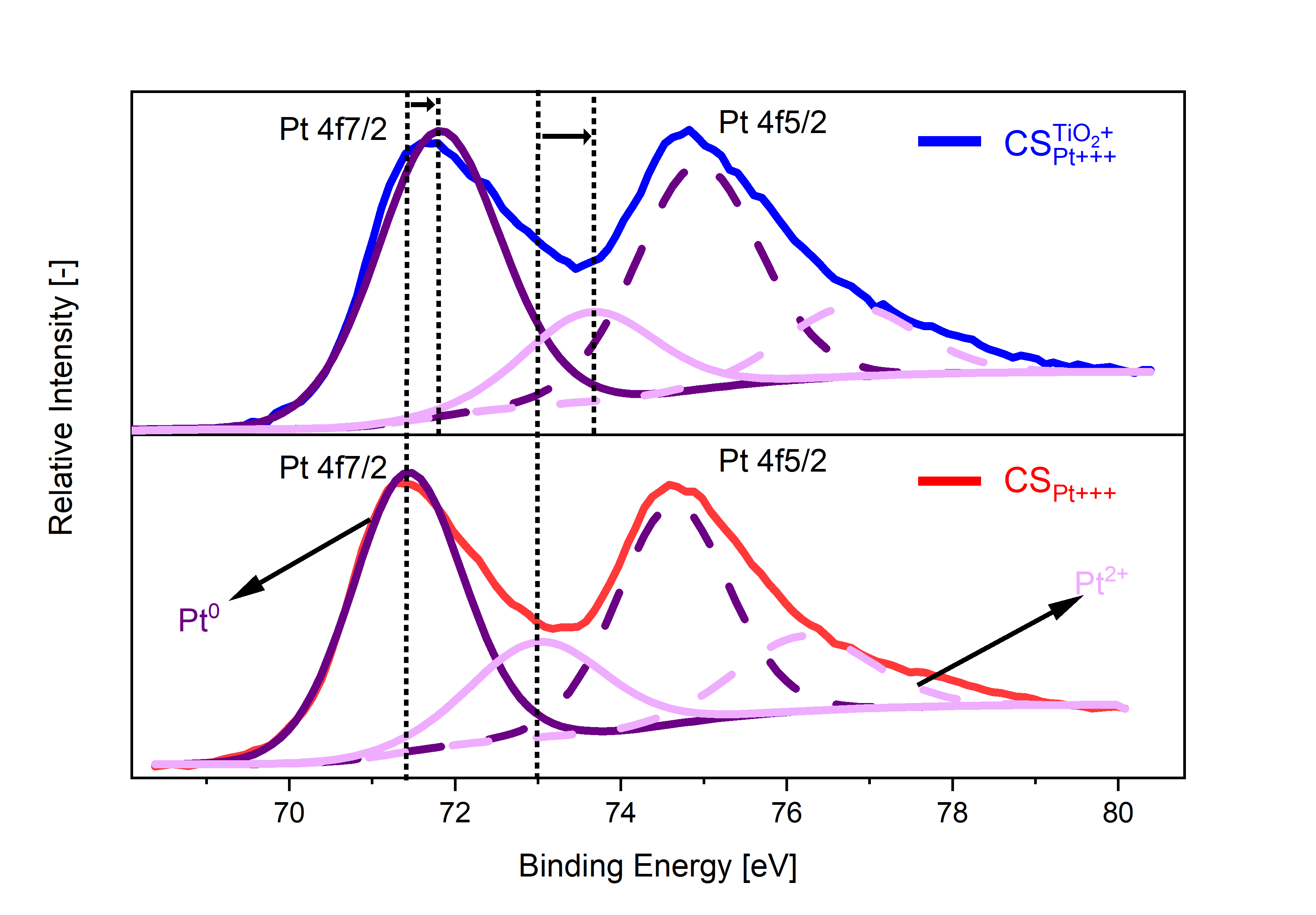


Figure S21 XPS analysis for Pt 4f Peak for $\mathbf{CS}_{\mathbf{Pt+++}}$ and $\mathbf{CS}_{\mathbf{Pt +++}}^{\mathbf{TiO2+}}$.


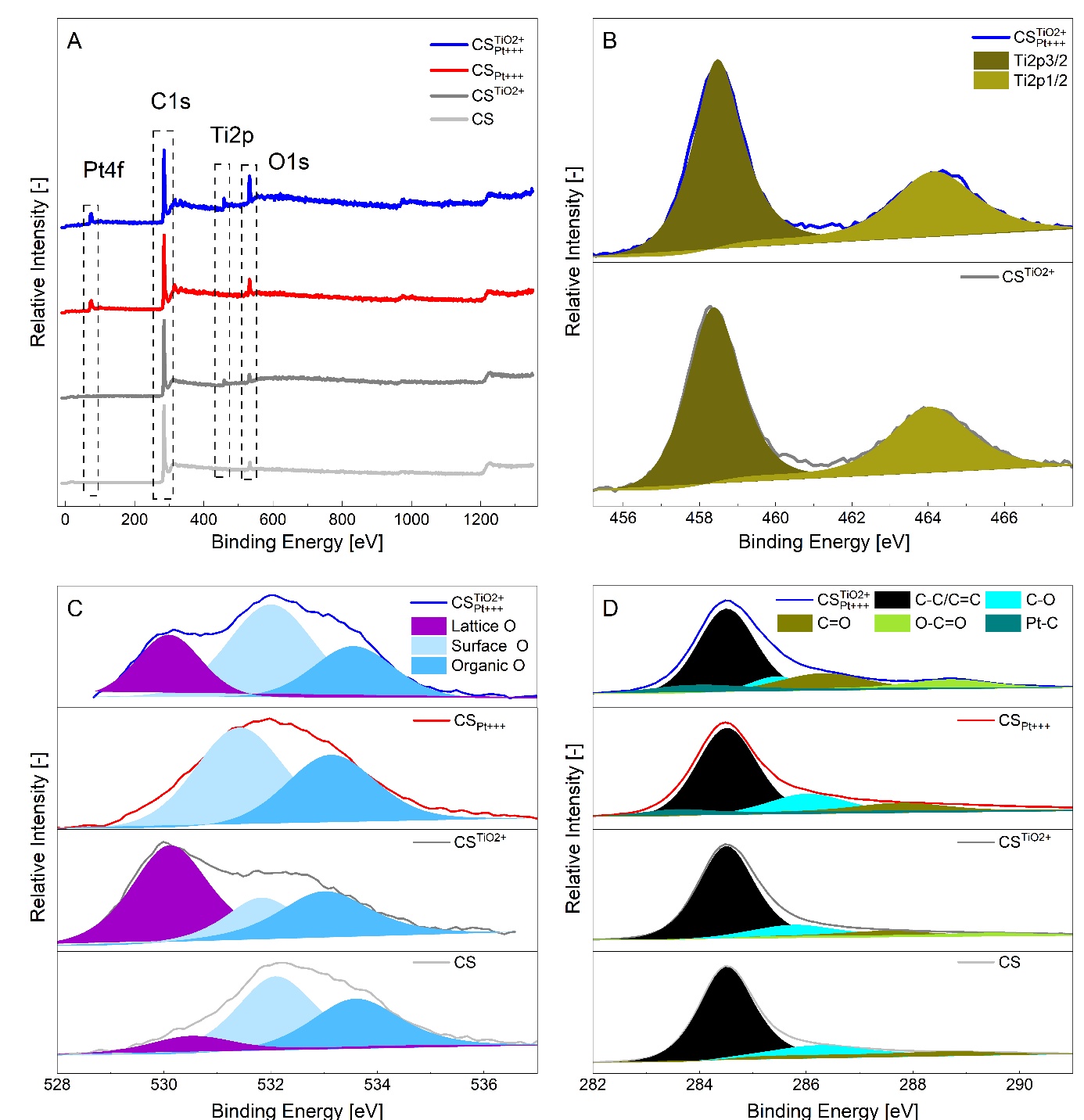


**Figure S22** XPS analysis of the: (A) Survey spectrum; and deconvolutions of (B) Ti 2p peak; (C) O 1s peak; and (D) C 1s peak.

Table S5 Binding energies functional groups in $\mathbf{C}\mathbf{S}^{\mathbf{TiO2+}}$, $\mathbf{C}\mathbf{S}_{\mathbf{Pt+++}}$, and $\mathbf{C}\mathbf{S}_{\mathbf{Pt+++}}^{\mathbf{TiO2+}}$, determined via XPS analysis. n. d. = not detected.

| Functional Group | Binding Energy [eV] | | | |
| --- | --- | --- | --- | --- |
|  | $\mathbf{CS}$ | $\mathbf{C}\mathbf{S}^{\mathbf{TiO2+}}$ | $\mathbf{CS}_{\mathbf{Pt+++}}$ | $\mathbf{CS}_{\mathbf{Pt +++}}^{\mathbf{TiO2+}}$ |
| –C | 284.5 | 284.5 | 284.5 | 284.5 |
| C–O | 286.24 | 285.8 | 286.0 | 285.4 |
| C=O | 288.73 | 287.5 | 287.8 | 286.3 |
| O–C=O | n. d. | 289.5 | n. d. | 288.7 |
| Pt–C bond | n. d. | n. d. | 283.6 | 283.9 |
| Lattice-O | 530.53 | 530.1 | N.D. | 530.1 |
| Surface-O | 532.09 | 531.8 | 531.4 | 532.0 |
| Organic-O | 533.58 | 533.0 | 533.1 | 533.6 |
| Pt⁰ | n. d. | n. d. | 71.4 | 71.8 |
| Pt²⁺ | n. d. | n. d. | 73.0 | 73.7 |
| Ti⁴⁺ | n. d. | 458.4 | n. d. | 458.5 |

Table S6 The atomic percentages of functional groups in $\mathbf{C}\mathbf{S}^{\mathbf{TiO2+}}$, $\mathbf{C}\mathbf{S}_{\mathbf{Pt+++}}$, and $\mathbf{C}\mathbf{S}_{\mathbf{Pt+++}}^{\mathbf{TiO2+}}$ , determined via XPS analysis. n. d. = not detected.

| Element | Functional Group | Atomic % | | | |
| --- | --- | --- | --- | --- | --- |
|  |  | $\mathbf{CS}$ | $\mathbf{C}\mathbf{S}^{\mathbf{TiO2+}}$ | $\mathbf{CS}_{\mathbf{Pt+++}}$ | $\mathbf{CS}_{\mathbf{Pt +++}}^{\mathbf{TiO2+}}$ |
| C | **Total** | 95.3 | 92.9 | 89.8 | 81.1 |
|  | **C–C** | 78.3 | 77.2 | 67.9 | 59.6 |
|  | **C–O** | 13.2 | 13.7 | 17.0 | 7.5 |
|  | **C=O** | 3.8 | 5.7 | 8.8 | 15.4 |
|  | **O–C=O** | n. d. | 3.3 | n. d. | 8.6 |
|  | **Pt–C bond** | n. d. | n. d. | 6.4 | 8.9 |
| O | **Total** | 4.7 | 5.7 | 9.1 | 15.5 |
|  | **Lattice-O** | 13.3 | 53.8 | n. d. | 24.9 |
|  | **Surface-O** | 50.0 | 19.3 | 59.8 | 49.4 |
|  | **Organic-O** | 36.7 | 27.0 | 40.2 | 24.9 |
| Pt | **Total** | n. d. | 0 | 1.1 | 1.0 |
|  | **Pt⁰** | n. d. | n. d. | 70.9 | 75.0 |
|  | **Pt²⁺** | n. d. | n. d. | 29.1 | 25.0 |
| Ti | **Total** | n. d. | 1.4 | 0.0 | 2.4 |
|  | **Ti⁴⁺** | n. d. | 100.0 | n. d. | 100.0 |


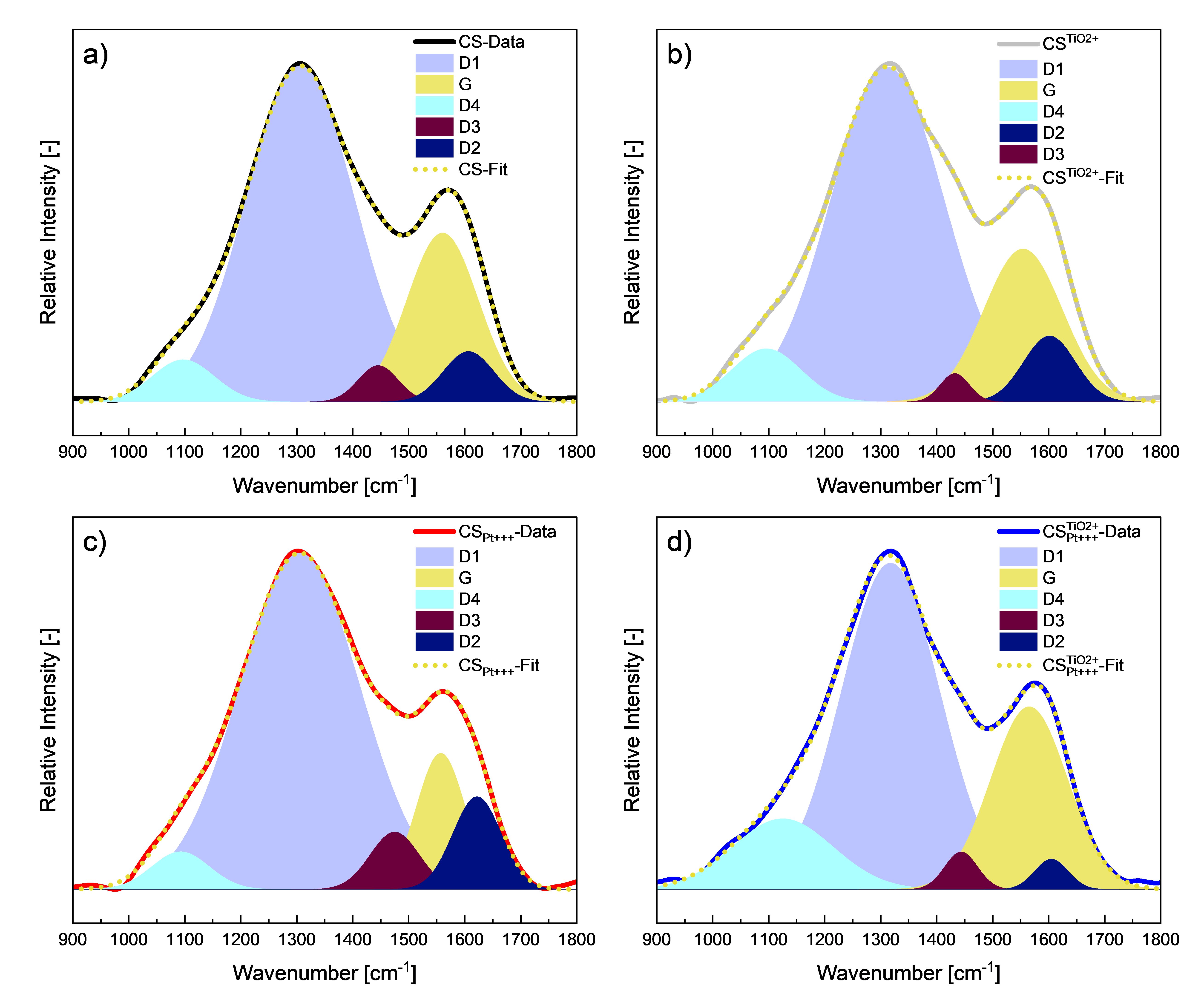


Figure S23 Raman analysis for (a) CS, (b) $\mathbf{C}\mathbf{S}^{\mathbf{TiO2+}}$, (c) $\mathbf{C}\mathbf{S}_{\mathbf{Pt+++}}$, and (d) $\mathbf{C}\mathbf{S}_{\mathbf{Pt+++}}^{\mathbf{TiO2+}}$.

Table S7: Deconvoluted Raman band positions of $\mathbf{CS}$, $\mathbf{C}\mathbf{S}^{\mathbf{TiO2+}}$, $\mathbf{C}\mathbf{S}_{\mathbf{Pt+++}}$, and $\mathbf{C}\mathbf{S}_{\mathbf{Pt+++}}^{\mathbf{TiO2+}}$ samples and their corresponding structural assignments.^[S4-S7]^

| Raman Band | Structural Assignment | Raman band position (cm^-1^) | | | |
| --- | --- | --- | --- | --- | --- |
|  |  | $\mathbf{CS}$ | $\mathbf{C}\mathbf{S}^{\mathbf{TiO2+}}$ | $\mathbf{CS}_{\mathbf{Pt+++}}$ | $\mathbf{CS}_{\mathbf{Pt +++}}^{\mathbf{TiO2+}}$ |
| G | Graphitic sp² carbon | 1560 | 1554 | 1557 | 1565 |
| D1 | Disordered sp² carbon | 1306 | 1312 | 1306 | 1318 |
| D2 | Surface graphene layers | 1607 | 1602 | 1622 | 1605 |
| D3 | Amorphous carbon | 1446 | 1432 | 1475 | 1444 |
| D4 | Polyenic/ionic impurities | 1097 | 1096 | 1093 | 1125 |


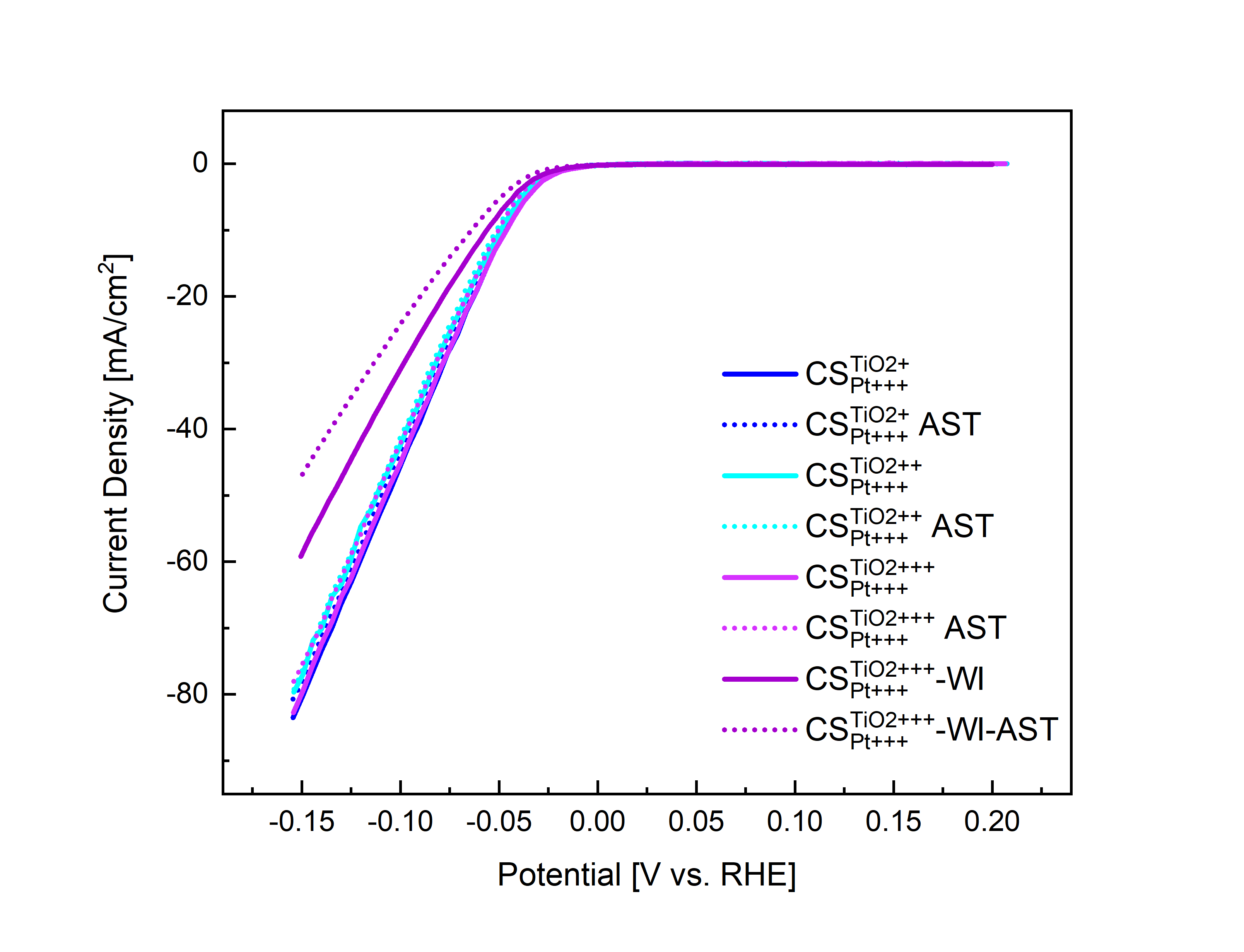


Figure S24 LSV test before and after ASTs (1000 cycles at 100 mV s^-1^) for all samples which were not shown in the main text.


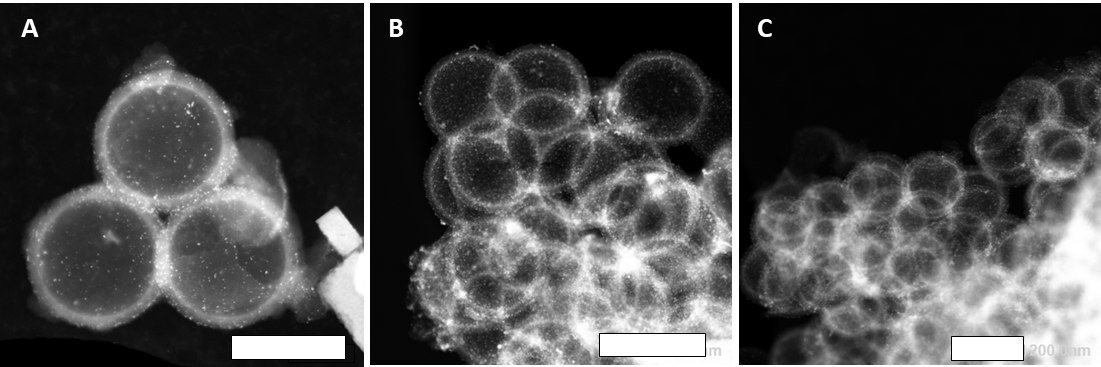
**Figure S25** STEM HAADF images of postmortem samples A) $\mathrm{CS}_{Pt+++}$ after AST, B) $\mathrm{CS}_{Pt+++}^{TiO2+}$ after AST, C) $\mathrm{CS}_{Pt+++}^{TiO2+}$ after the durability test. Scale bars correspond to 200 nm.


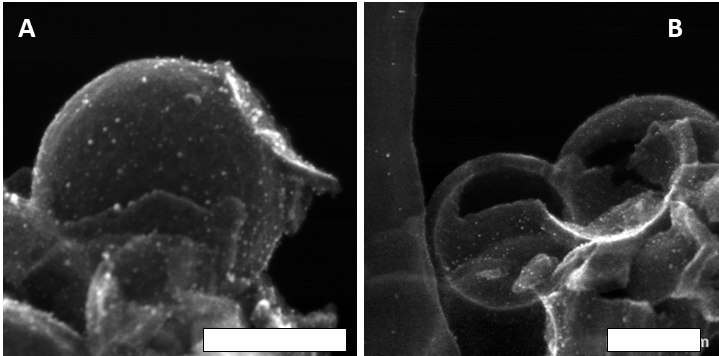


Figure S26 STEM HAADF images of individual broken spheres in postmortem samples. A) $\mathbf{CS}_{\mathbf{Pt+++}}^{\mathbf{TiO2+}}$ after the durability test, B) $\mathbf{CS}_{\mathbf{Pt+++}}^{\mathbf{TiO2+}}$ after AST. Scale bars correspond to 100 nm.


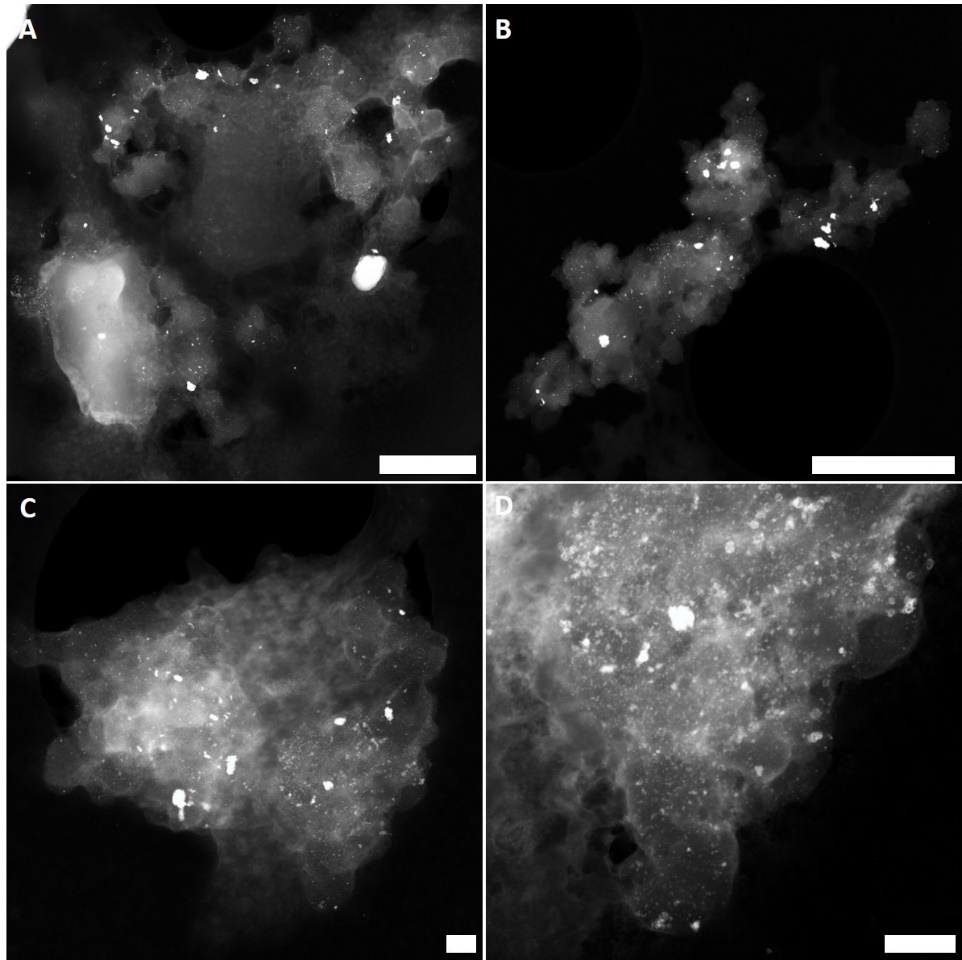


Figure S27 STEM HAADF images of the commercial electrocatalyst C_Pt+++_ after AST (post- mortem), different magnifications. Scale bars represent 1000 nm in images A), B) and 100 nm in images C), D).

Table S8 Literature values for overpotential at 10 mA/cm^2^ and potential shift upon stability test.

| Work | Electrocatalyst | Overpotential @ 10mA/cm^2^ [mV] | Overpotential @ 10mA/cm^2^ [mV]* | Difference in overpotential [mV]** | Stability test of # cycles  [-] | Potential shift [mV] |
| --- | --- | --- | --- | --- | --- | --- |
| [S8]  *Small* 2024 | Pt-WC/CNT | 25.0 | 31.0 | **6.0** | 1000 | 9 mV @ 50 mA/cm^2^ |
| [S9]  *Small* 2023 | Pt-ACs/S-TiO₂-NP | 17.0 | 26.0 | **9.0** | 10 000 | 7 mV @ 10 mA/cm^2^ |
| [S10]  *Small* 2024 | Pt@TiVCO | 37.0 | ≈46 | **~ 9** | 10 000 | 4 mV @ 10 mA/cm^2^ |
| [S11]  *Small* 2024 | Pt@N-CTs-1.04 | 44.5 | 33.9 | **−10.6** | - | - |
| [S12]  *Small* 2024 | MPNCCPt- pCVD-250-250-T | 9.0 | 17.0 | **8.0** | - | - |
| [S13]  2024 | Pt/TE-UTPNBs | 28.0 | ≈20 | **−8** | - | - |
| [S14]  *Small* 2023 | PZ@VC-N (2 wt%) | 7.0 | 12.0 | **5.0** | - | - |
| [S15]  *Small* 2023 | Pt/CNTs-N + α-MoC1 - x | 17.0 | 17.0 | **0.0** | - | - |
| [S16]  *Adv. Energy Mater.* 2023 | Pt@VNC | 5.0 | 14.0 | **9.0** | - | - |
| [S17]  *Adv. Funct. Mater.* 2022 | 0.5 wt% Pt–TiVC | 24.9 | 33.3 | **8.4** | 5000 | ≈0 mV @ 10 mA/cm^2^ |
| [S18]  *ACS Nano* 2023 | PtM@PC800 | 3.4 | 18.0 | **14.0** | - | - |
| [S19]  *Angew. Chem. Int. Ed.* 2023 | Pt/TiO₂-OV | 18.0 | 35.0 | **17.0** | 10000 CV | 4 mV @ 10 mA/cm^2^ |
| [S20]  *Small* 2022 | PANI@Pt/S-TiN NTs/Ti | 12.0 | 8.0 | **-4.0** | 3000 CV | ≈0 mV @ 10 mA/cm^2^ |
| This work | Pt-TiO_2_-Pt 10 wt% | 46.0 | 77.0 | **31.0** | 1000 CV | 1 mV @ 10 mA/cm^2^ |

* Values of individual Pt/C commercial electrocatalysts used in respective works.

** Difference between self-developed electrocatalysts and commercial electrocatalysts used in respective works.


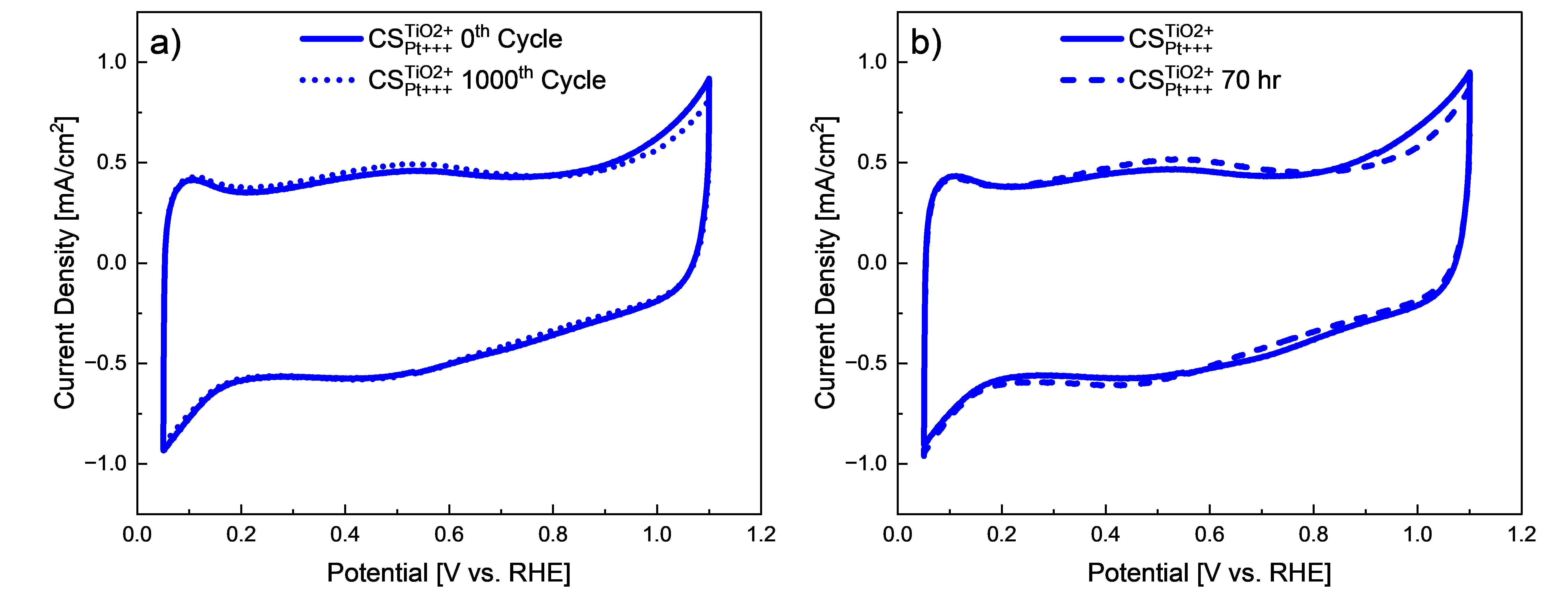


Figure S28 CV test of $\mathbf{CS}_{\mathbf{Pt+++}}^{\mathbf{TiO2+}}$ (a) after AST and (b) after the 70h durability test.


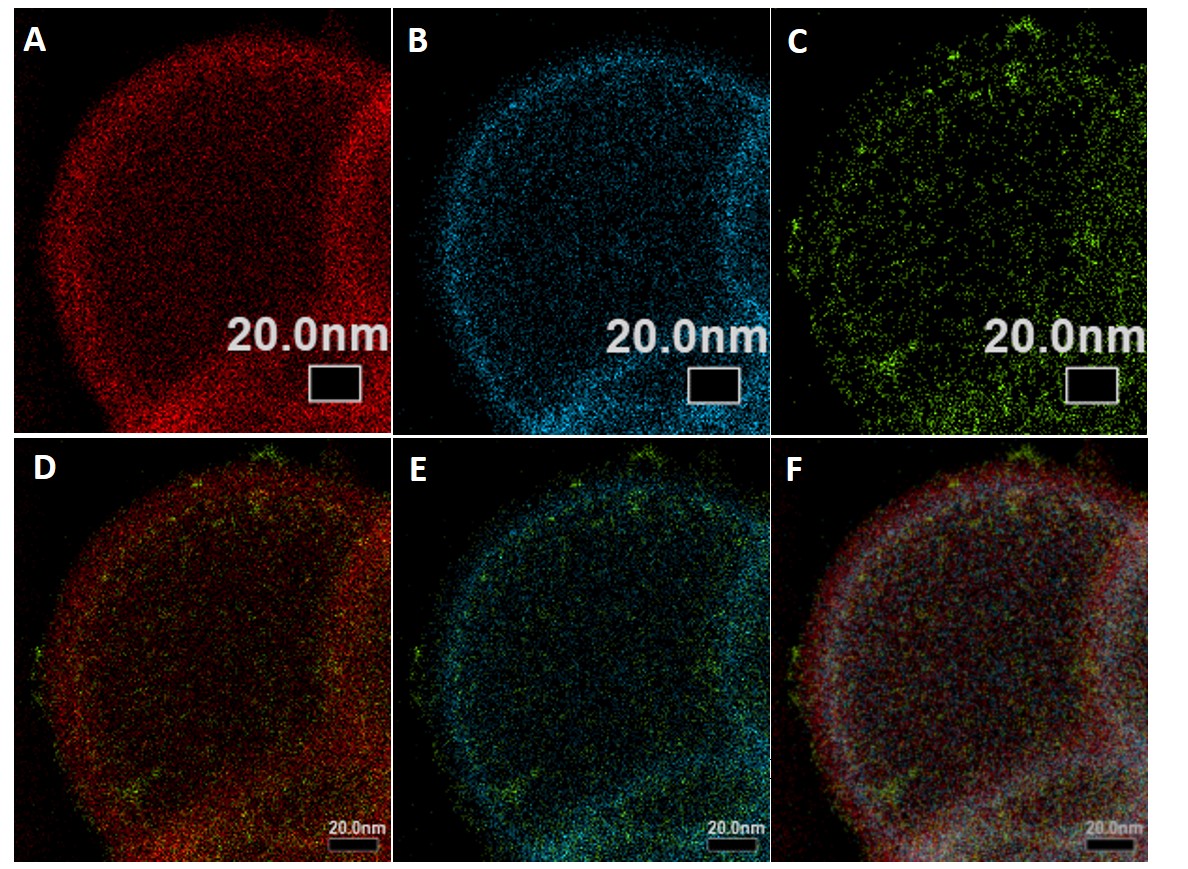


Figure S29 EDX overlapped elemental maps of $\mathbf{CS}_{\mathbf{Pt+++}}^{\mathbf{TiO2+}}$ after the 70h durability test. Top: single element images of C (A), Ti (B) and Pt (C). Bottom: overlapped elemental maps of C + Pt (D), TiO_2_ + Pt (E) and C + TiO_2_ + Pt (F).

# Alternative catalyst use – propane dehydrogenation

To evaluate the potential of Pt-NPs on spherogel-based carbon systems for other applications, we have tested the capability of the $\mathrm{CS}_{Pt+++}$-sample for catalysis of selective propane dehydrogenation. In each catalytic test, 22 mg of $\mathrm{CS}_{Pt+++}$-catalyst was fixed with quartz wool in a homebuilt 8 mm (inner diameter) quartz tube flow reactor setup under atmospheric pressure and a total flow rate of 165 mL min^-1^ (C_3_H_8_ : He = 1 : 99 mL min^-1^). The reactor was heated with a tubular furnace (Linn High Therm) at 6 °C min^-1^ to 800 °C followed by an isothermal period at 800 °C for 5 min. An external S-type thermocouple (calibrated by an independent experiment with flowing helium) was placed in close contact to the reactor tube to ensure correct temperature reading. The output gas was directly detected by a quadrupole mass spectrometer (Balzers QME 125). For quantitative analysis, the relevant gases were detected and calibrated via their respective molecular ions and fragmentations: C_3_H_8_ (m/z = 29), C_3_H_6_ (m/z = 41), C_2_H_4_ (m/z = 26), CH_4_ (m/z = 16), He (m/z = 4) and H_2_ (m/z=2). Simultaneous recording of these mass signals allowed to disentangle the superimposed intensity contributions of different components. To display the catalytic activity as a function of temperature, we use the following equation (**Eq. S16**) to calculate the propane conversion:

| $Propane conversion \left[ \% \right]=100\cdot(1-\frac{\left[ C_{3}H_{8} \right]_{\mathrm{out}}}{\left[ C_{3}H_{8} \right]_{\mathrm{in}}})$ | **Eq. S16** |
| --- | --- |

$\left[ C_{3}H_{8} \right]_{out}$ and $\left[ C_{3}H_{8} \right]_{in}$ indicate the inlet and outlet concentration of propane, respectively.

$$C_{3}H_{6} selectivity=100\cdot(\frac{\mathrm{moles}C_{3}H_{6}\mathrm{produced}}{\mathrm{moles}C_{3}H_{8}\mathrm{reacted}})$$

$$C_{2}H_{4} selectivity=100\cdot(\frac{2\cdot moles C_{2}H_{4}\mathrm{produced}}{3\cdot moles C_{3}H_{8}\mathrm{reacted}})$$

$$CH_{4} selectivity=100\cdot(\frac{\mathrm{moles}CH_{4}\mathrm{produced}}{3\cdot moles C_{3}H_{8}\mathrm{reacted}})$$

The dehydrogenation of propane (DHP) to propylene (**Eq. S17**) is an important process to access propylene as a valuable feedstock for the petrochemical industry.

| $C_{3}H_{8}\rightleftharpoons C_{3}H_{6}+H_{2}$ | **Eq. S17** |
| --- | --- |

The main reaction (**Eq. S17**) appears simple, but the entire reaction is very complex due to additional cracking and coking reactions. Reaction (**Eq. S17**) is strongly endothermic (standard formation enthalpy${\Delta H}_{298}=+124.3 kJ/mol)$ and as such, the DHP process is usually carried out slightly below 650 °C. It is worth noting that all technological processes exhibit limited propane conversions between 32-55% with associated propylene selectivities between 87 and 91%. Supported Pt catalysts with and without promoter are commonly used in the commercial DHP process and have been scrutinized in literature.^[S21]^

Our results (**Figure S21**) indicate a reaction temperature window between 550 °C and 650 °C, with almost 100 % propylene selectivity at, however, quite low conversions below 10%. Above 650 °C, the propane conversion rapidly increases to ca. 50%, associated with a propylene selectivity decrease to 30%. Formation of ethylene is pronounced at ca. 56%, with a minor contribution of methane (ca. 14%). While this experiment verifies the potential of the materials to be used as selective catalysts, further investigation with higher Pt content and/or presence of TiO_2_ could be useful to evaluate their full potential in upcoming works.


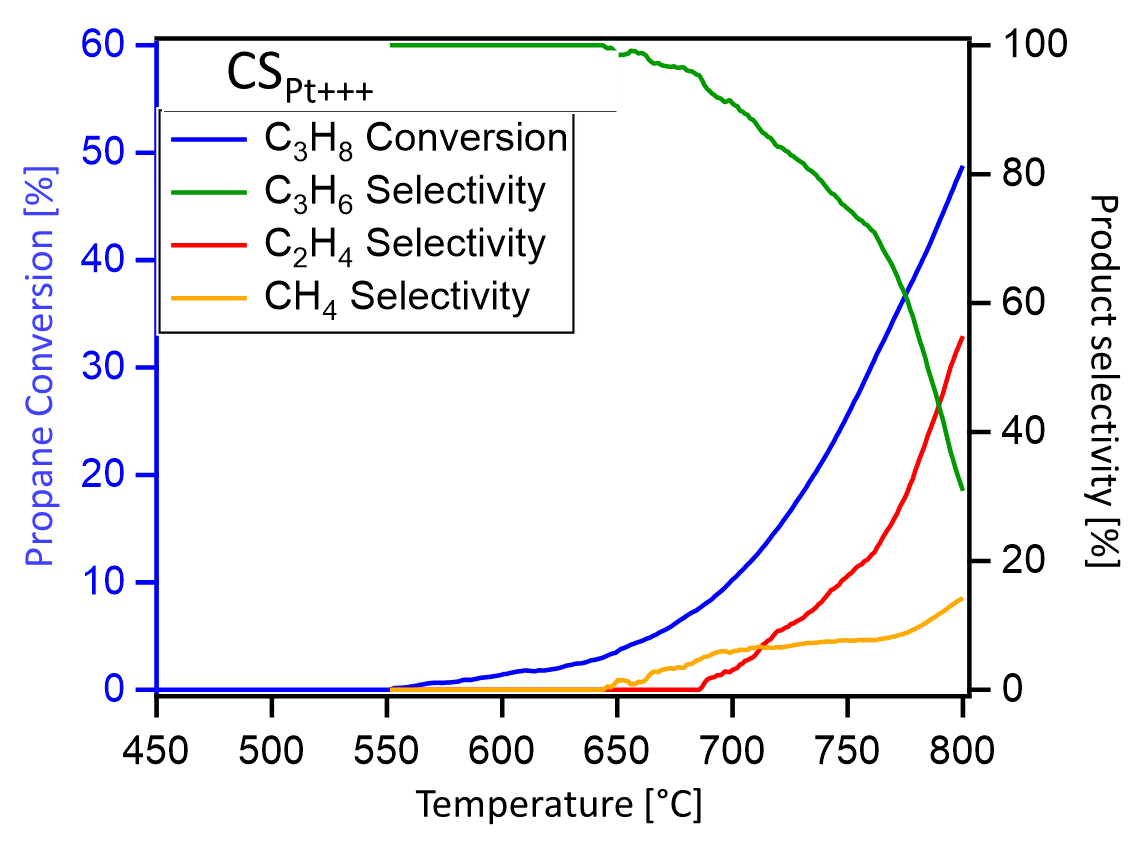


Figure S30 Propane conversion and product selectivity over temperature for CS_Pt+++_.

**References SI**

[S1] Pozio, A.; De Francesco, M.; Cemmi, A.; Cardellini, F.; Giorgi, L. Comparison of high surface Pt/C catalysts by cyclic voltammetry. J. Power Sources, **2002**, 105(1), 13 – 19. https://doi.org/10.1016/S0378-7753(01)00921-1

[S2] Trasatti, S.; Petrii, O. Real Surface Area Measurements in Electrochemistry, J. Electroanal. Chem., 1992, 327, 353 – 376. https://doi.org/10.1351/PAC199163050711

[S3] Moniri, S.; Van Cleve, T.; Linic, S. Pitfalls and best practices in measurements of the electrochemical surface area of platinum-based nanostructured electrocatalysts, J. Catal., 2017, 345, 1 – 10. https://doi.org/10.1016/j.jcat.2016.11.018

[S4] Sadezky, A.; Muckenhuber, H.; Grothe, H-; Niessner, R.; Pöschl, U. Raman microspectroscopy of soot and related carbonaceous materials: Spectral analysis and structural information, Carbon, 2005, 43(8), 1731-1742. https://doi.org/10.1016/j.carbon.2005.02.018.

[S5] Barım, Ş. B.; Bayrakçeken, A.; Bozbağ, S. E.; Zhang, L.; Kızılel, R.; Aindow, M.; Erkey, C. Control of average particle size of carbon aerogel supported platinum nanoparticles by supercritical deposition, Microporous Mesoporous Mater., 2017, 245, 94-103. https://doi.org/10.1016/j.micromeso.2017.01.037

[S6] Ferrari, A. C.; Robertson, J. Raman spectroscopy of amorphous, nanostructured, diamond-like carbon, and nanodiamond, Philos. Trans. R. Soc. A, 2004, 362(1824), 2477–2512. https://doi.org/10.1098/rsta.2004.1452

[S7] Ferrari, A. C.; Robertson, J. Interpretation of Raman spectra of disordered and amorphous carbon, Phys. Rev. B, 2000, 61(20), 14095–14107. https://doi.org/10.1103/PhysRevB.61.14095

[S8] Chen, X.; et al. Pt-WC/CNT for efficient hydrogen evolution. Small, 2024, 20, 2309675. https://doi.org/10.1002/smll.202309675

[S9] Wang, Y.; et al. Pt-ACs/S-TiO₂-NP hybrid as HER electrocatalyst. Small, 2023, 19, 2303495. https://doi.org/10.1002/smll.202303495

[S10] Zhang, L.; et al. Pt@TiVCO nanocomposites for HER. Small, 2024, 20, 2309791. https://doi.org/10.1002/smll.202309791

[S11] Pan, J.; et al. Pt@N-CTs-1.04 with superior HER stability. Small, 2024, 20, 2309823. https://doi.org/10.1002/smll.202309823

[S12] Küspert, J.; et al. MPNCCPt-pCVD-250-250-T. Small, 2024, 20, 2309067. https://doi.org/10.1002/smll.202309067

[S13] Wu, Y.; et al. Pt/TE-UTPNBs for hydrogen electrocatalysis. Adv. Mater., 2024, 36, 2312724. https://doi.org/10.1002/adma.202312724

[S14] Chakraborty, I.; et al. PZ@VC-N electrocatalysts. Small, 2023, 19, 2207146. https://doi.org/10.1002/smll.202207146

[S15] Cai, H.; et al. Pt/CNTs-N + α-MoC1-x for hydrogen evolution. Small, 2023, 19, 2210665. https://doi.org/10.1002/smll.202210665

[S16] Jin, S.; et al. Pt@VNC with enhanced durability. Adv. Energy Mater., 2023, 13, 2204213. https://doi.org/10.1002/aenm.202204213

[S17] Dong, C.; et al. 0.5 wt% Pt–TiVC HER catalyst. Adv. Funct. Mater., 2022, 33, 2205603. https://doi.org/10.1002/adfm.202205603

[S18] Li, K.; et al. PtM@PC800 nanostructures. ACS Nano, 2023, 17, 2923–2931. https://doi.org/10.1021/acsnano.2c11272

[S19] Wu, X.; et al. Pt/TiO₂-OV as efficient HER electrocatalyst. Angew. Chem. Int. Ed., 2023, 62, e202300406. https://doi.org/10.1002/anie.202300406

[S20] Yan, Y.; et al. PANI@Pt/S-TiN NTs/Ti nanostructures. Small, 2022, 18, 2205603. https://doi.org/10.1002/smll.202205603

[S21] Sun, M.; Zhai, S.; Weng, C.; Wang, H.; Yuan, Z.-Y. Pt-based catalysts for direct propane dehydrogenation: Mechanisms revelation, advanced design, and challenges. Mol. Catal., **2024**, 558, 114029. https://doi.org/10.1016/j.mcat.2024.114029
